# Supplementary material for: Family satisfaction with critical care in the UK: a multicentre cohort study
Source: BMJ Open. 2019 Aug 20;9(8):e028956. doi: 10.1136/bmjopen-2019-028956 (PMC6707657; doi:10.1136/bmjopen-2019-028956)
Supplement: Supplementary data [file bmjopen-2019-028956supp001.pdf]

Supplementary material

Family satisfaction with critical care in the United Kingdom: a multi-centre cohort study

Table S1 Characteristics and outcomes for all admission to ICUs participating in the FREE study and ICNARC Case Mix Programme

	**CMP**	**FREE study**
Total number of ICUs [N]	[209][a]	[19][a]
Total number of admissions [N]	[149,779]	[18,270]
Age *mean* (SD)	61.5 (18.0)	61.5 (18.0)
Sex *male* (%)	82,444 (55.0)	10,316 (56.5)
Ethnicity *n* (%)		
White	135,767 (90.6)	16,439 (90.0)
Asian	4,815 (3.2)	439 (2.4)
Black	3,250 (2.2)	327 (1.8)
Other	2,434 (1.6)	445 (2.4)
Not stated	3,513 (2.3)	620 (3.4)
Distance (km) from patient home to hospital *median* (IQR) [N]	25.0 (54.2) 8.7 (3.9 19.3) [128,169]	31.7 (64.5) 9.2 (4.2 20.8) [18,090]
APACHE II severe co-morbidities *n* (%)		
0	123,437 (82.4)	14,742 (80.7)
1	20,906 (14.0)	2,648 (14.5)
2	5,053 (3.4)	793 (4.3)
3 or more	383 (0.3)	87 (0.5)
Admission type *n* (%) [N]	[149,765]	[18,270]
Medical	87,940 (58.7)	10,039 (54.9)
Elective surgery	34,284 (22.9)	4,761 (26.1)
Emergency surgery	27,541 (18.4)	3,470 (19.0)
Surgical status of surgical admissions *n* (%) [N]	[61,825]	[8,231]
Planned surgery	28,267 (45.7)	3,985 (48.4)
Unplanned surgery	33,558 (54.3)	4,246 (51.6)
ICNARC Physiology Score *mean* (SD)	16.9 (9.3)	16.5 (9.2)
ICNARC predicted risk of death *median* (IQR) [N]	0.10 (0.03 0.33) [142,654]	0.09 (0.03 0.30) [17,261]
APACHE II Acute Physiology Score *mean* (SD)	11.4 (6.1)	11.3 (5.9)
APACHE II Score *mean* (SD)	15.7 (7.0)	15.6 (6.9)
APACHE II predicted risk of death *median* (IQR) [N]	0.12 (0.04 0.29) [132,197]	0.11 (0.04 0.28) [16,193]
Mechanical ventilation during first 24 hrs *n* (%) [N]	58,687 (39.4) [148,975]	7,008 (38.5) [18,187]

ICU mortality *n* (%) [N]	21,505 (14.4) [149,779]	2,560 (14.0) [18,270]
Acute hospital mortality *n* (%) [N]	29,945 (21.0) [142,670]	3,550 (20.6) [17,266]

[a] excludes one ICU for which no CMP data were available

Figure S1 Overview of patients, family members and questionnaires (distributed/returned)

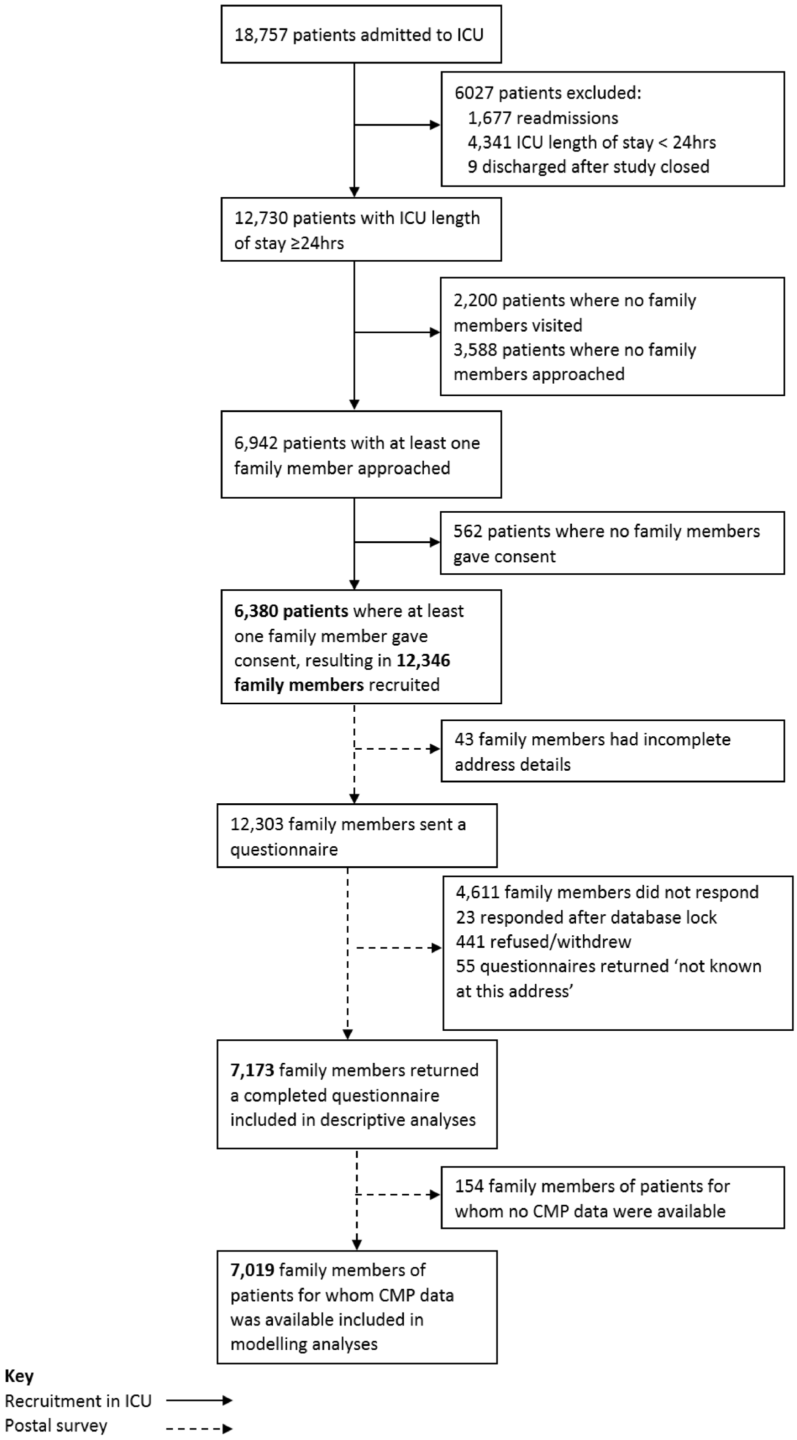

Table S2 Characteristics of all recruited family members and by response to questionnaire

	**All recruited family members**	**Those returning questionnaires**	**Did not respond**
Total number of family members, N	12 346	7173	4611
Age group, *n* (%) [N]	[12 068]	[7019]	[4500]
<30	1429 (11.8)	530 (7.6)	861 (19.1)
30-39	1590 (13.2)	721 (10.3)	827 (18.4)
40-49	2760 (22.9)	1465 (20.9)	1208 (26.9)
50-59	2646 (21.9)	1654 (23.6)	886 (19.7)
60-69	2131 (17.7)	1580 (22.5)	440 (9.8)
70-79	1211 (10.0)	862 (12.3)	220 (4.8)
80+	301 (2.5)	207 (2.9)	58 (1.3)
Sex, *n* (%) [N]	[12 145]	[7062]	[4529]
Female	7687 (63.3)	4689 (66.4)	2663 (58.8)
Male	4458 (36.7)	2373 (33.6)	1866 (41.2)
Ethnicity, *n* (%) [N]	[12 090]	[7033]	[4505]
White	11 379 (94.1)	6747 (95.9)	4111 (91.3)
Asian	355 (2.9)	142 (2.0)	196 (4.4)
Black	161 (1.3)	55 (0.8)	101 (2.2)
Other	195 (1.6)	89 (1.3)	97 (2.1)
Deprivation, *n* (%) [N]	[11 740]	[6832]	[4370]
1 [least deprived]	2113 (18.0)	1376 (20.1)	634 (14.5)
2	2406 (20.5)	1502 (22.0)	803 (18.4)
3	2415 (20.6)	1443 (21.1)	851 (19.5)
4	2545 (21.7)	1380 (20.2)	1045 (23.9)
5 [most deprived]	2261 (19.3)	1131 (16.6)	1037 (23.7)
Distance (km) from family member home to hospital, *median* (IQR) [N]	11.6 (5.1-30.7) [11 803]	12.3 (5.3-33.2) [6867]	10.7 (4.6-29.4) [4394]
Relationship, *n* (%) [N] "I am the patient's…"	[12 343]	[7173]	[4611]
Partner	3105 (25.2)	2151 (30.0)	786 (17.0)
Child	4186 (33.9)	2292 (32.0)	1780 (38.6)
Parent	1054 (8.5)	665 (9.3)	338 (7.3)
Sibling	1271 (10.3)	717 (10.0)	480 (10.4)
Other relative	1973 (16.0)	987 (13.8)	898 (19.5)
Other non-relative	754 (6.1)	361 (5.0)	329 (7.1)
Next-of-kin, *n* (%) [N]	[11 702]	[6770]	[4389]
No	7086 (60.6)	3747 (55.3)	3009 (68.6)
Yes	4616 (39.4)	3023 (44.7)	1380 (31.4)
Lives with patient, *n* (%) [N]	[12 343]	[7172]	[4609]
No	8255 (66.9)	4543 (63.3)	3357 (72.8)
Yes	4088 (33.1)	2629 (36.7)	1252 (27.2)
Education level, *n* (%) [N]	[10 293]	[5971]	[3888]
NVQ 1 or 2	3147 (30.6)	1731 (29.0)	1284 (33.0)
NVQ 3	2086 (20.3)	1149 (19.2)	870 (22.4)

NVQ 4 or 5	2936 (28.5)	1819 (30.5)	1032 (26.5)
Other	2124 (20.6)	1272 (21.3)	702 (18.1)
First language, *n* (%) [N]	[12 346]	[7 173]	[4611]
Not English	335 (2.7)	140 (2.0)	182 (3.9)
English	12 011 (97.3)	7 033 (98.0)	4429 (96.1)

Table S3 Univariable analyses of factors associated with overall family satisfaction score by ICU outcome – family member characteristics

**Variables**	**Family members of ICU survivors [N=6,147[a]]**			**Family members of ICU non-survivors [N=870]**		
	**Coef.**	**95% CI**	**p-value**	**Coef.**	**95% CI**	**p-value**
Age, years (vs < 30)			0.031			0.033
30-39	1.56	(-0.22, 3.33)		2.68	(-1.80, 7.17)	
40-49	0.42	(-0.10, 0.94)		1.61	(0.21, 3.01)	
50-59	2.12	(0.61, 3.64)		5.49	(1.49, 9.50)	
60-69	1.96	(0.39, 3.52)		6.01	(1.78, 10.25)	
70-79	1.98	(0.28, 3.68)		7.39	(2.58, 12.19)	
80+	-0.55	(-3.05, 1.95)		2.62	(-3.48, 8.73)	
Female (vs male)	0.40	(-0.34, 1.14)	0.29	0.44	(-1.59, 2.47)	0.67
White ethnicity (vs non-white)	3.60	(1.46, 5.75)	0.001	8.78	(1.85, 15.70)	0.013
Relationship (vs partner)			<0.001			0.28
Parent	0.00	(-1.39, 1.39)		0.08	(-5.73, 5.90)	
Child	-0.94	(-1.83, -0.05)		-1.274	(-3.69, 1.14)	
Sibling	-2.16	(-3.50, -0.82)		0.909	(-3.02, 4.84)	
Other-relative	-1.63	(-2.81, -0.44)		-0.619	(-3.60, 2.36)	
Other-non relative	-3.42	(-5.22, -1.62)		-6.134	(-11.69, -0.58)	
Next of kin	1.74	(1.05, 2.44)	<0.001	2.69	(0.78, 4.59)	0.006
Lives with patient	1.95	(1.20, 2.69)	<0.001	1.15	(-0.99, 3.29)	0.29
Education (vs NVQ 1 or 2)			<0.001			0.16
NVQ 3	-0.60	(-1.77, 0.57)		1.14	(-2.09, 4.37)	
NVQ 4 or 5	-2.43	(-3.49, -1.37)		-2.07	(-4.92, 0.77)	
Other	-0.18	(-1.35, 0.98)		-1.75	(-4.73, 1.24)	
Quintile of deprivation (vs 1, least deprived)			0.63			0.77
2	0.49	(-0.74, 1.72)		0.64	(-2.73, 4.01)	
3	0.96	(-0.29, 2.20)		0.84	(-2.59, 4.26)	
4	0.32	(-0.97, 1.60)		-1.07	(-4.59, 2.44)	
5 (most deprived)	0.67	(-0.70, 2.05)		0.79	(-3.10, 4.69)	
Distance from home to hospital (per 10 km)	-0.05	(-0.11, 0.01)	0.12	0.05	(-0.09, 0.18)	0.49
Previous experience of ICU as a family member	0.25	(-0.63, 1.14)	0.58	-0.68	(-3.22, 1.87)	0.60
Frequent visitor	2.52	(1.63, 3.41)	<0.001	2.91	(0.36, 5.47)	0.030

Coef., coefficient.

[a] Two family members returned questionnaires but did not complete any of the 24 FS-ICU items – responses were not imputed for these family members.

Table S4 Univariable analyses of factors associated with overall family satisfaction score by ICU outcome – patient characteristics

**Variables**	**Family members of ICU survivors [N=6,147[a]]**			**Family members of ICU non-survivors [N=870]**		
	**Coef.**	**95% CI**	**p-value**	**Coef.**	**95% CI**	**p-value**
Age (per 10 years)	-0.09	(-0.36, 0.17)	0.49	1.12	(0.11, 2.14)	0.030
Female (vs male)	0.67	(-0.25, 1.59)	0.16	2.04	(-0.66, 4.74)	0.14
White ethnicity (vs non-white)	2.39	(0.11, 4.68)	0.040	9.25	(2.38, 16.12)	0.008
Quintile of deprivation (vs 1, least deprived)			0.76			0.95
2	0.86	(-0.66, 2.38)		-1.28	(-5.85, 3.29)	
3	0.62	(-0.90, 2.13)		-0.68	(-5.12, 3.75)	
4	0.77	(-0.75, 2.28)		-1.62	(-6.03, 2.78)	
5 (most deprived)	1.00	(-0.57, 2.57)		-1.49	(-6.04, 3.06)	
Distance from home to hospital (per 10 km)	0.12	(0.00, 0.24)	0.047	0.18	(-0.05, 0.41)	0.12
Severe comorbidities						
Liver	3.18	(-0.01, 6.38)	0.050	1.25	(-4.67, 7.19)	0.68
Renal	-0.45	(-3.57, 2.66)	0.77	-8.87	(-18.35, 0.60)	0.067
Respiratory	0.01	(-2.84, 2.85)	1.00	-1.02	(-7.23, 5.19)	0.75
Cardiovascular	-0.14	(-3.23, 2.94)	0.93	1.40	(-6.46, 9.26)	0.73
Metastatic cancer	-2.81	(-5.78, 0.15)	0.063	3.26	(-6.38, 12.90)	0.51
Haematological malignancy	2.25	(-1.09, 5.61)	0.19	-7.88	(-14.62, -1.13)	0.022
Immunocompromise	-0.91	(-2.74, 0.90)	0.33	-3.90	(-8.55, 0.74)	0.10
Dependency (vs none)			0.30			0.85
Minor or major	-0.14	(-1.36, 1.08)		0.63	(-2.34, 3.60)	
Total	-3.63	(-8.21, 0.94)		2.73	(-10.21, 15.67)	
Surgical status (vs non-surgical)			0.005			0.78
Planned elective/scheduled	-2.17	(-3.51, -0.83)		-2.83	(-10.75, 5.10)	
Unplanned	-0.17	(-1.29, 0.96)		-0.06	(-3.89, 3.76)	
ICNARC Physiology Score (per point)	0.19	(0.13, 0.25)	<0.001	0.19	(0.02, 0.35)	0.026
ICU length of stay (per day)	0.02	(-0.03, 0.06)	0.44	-0.34	(-0.48, -0.20)	<0.001
Advanced respiratory support	3.62	(2.63, 4.61)	<0.001	1.96	(-1.84, 5.76)	0.31
Advanced cardiovascular support	2.06	(0.89, 3.22)	0.001	0.83	(-2.06, 3.72)	0.58
Renal support	1.52	(0.11, 2.93)	0.034	0.04	(-2.83, 2.91)	0.98
Neurological support	1.96	(0.39, 3.54)	0.014	2.95	(-0.42, 6.32)	0.086
Duration of adv. respiratory support (per day)	0.11	(0.05, 0.16)	<0.001	-0.16	(-0.32, 0.00)	0.051
Duration of adv. cardiovascular support (per day)	0.40	(0.15, 0.65)	0.002	0.11	(-0.33, 0.56)	0.62
Duration of renal support (per day)	0.16	(0.00, 0.32)	0.048	-0.15	(-0.43, 0.13)	0.28
Duration of neurological support (per day)	0.10	(-0.09, 0.29)	0.31	0.05	(-0.43, 0.53)	0.84
Death before acute hospital discharge	-0.49	(-1.52, 0.55)	0.36	N/A		

Coef., coefficient.

[a] Two family members returned questionnaires but did not complete any of the 24 FS-ICU items – responses were not imputed for these family members.

Table S5 Univariable analysis of factors associated with overall family satisfaction score by ICU outcome – ICU/hospital characteristics and contextual factors

**Variables**	**Family members of ICU survivors [N=6,147[a]]**			**Family members of ICU non-survivors [N=870]**		
	Coef.	95% CI	p-value	Coef.	95% CI	p-value
Hospital type (vs non-university)			0.51			0.62
University	0.06	(-3.63, 3.75)		-0.32	(-4.72, 4.07)	
University affiliated	1.93	(-1.56, 5.42)		1.68	(-2.29, 5.65)	
Number of ICU beds (per bed)	-0.05	(-0.23, 0.14)	0.63	0.02	(-0.22, 0.26)	0.85
Month of ICU admission (vs January)			0.95			0.85
February	-0.61	(-2.87, 1.65)		-0.03	(-6.90, 6.83)	
March	0.09	(-2.12, 2.30)		-0.06	(-6.73, 6.60)	
April	0.54	(-1.71, 2.79)		0.07	(-6.93, 7.07)	
May	-0.06	(-2.31, 2.18)		0.73	(-5.62, 7.08)	
June	-0.66	(-2.65, 1.34)		0.84	(-4.95, 6.64)	
July	0.85	(-1.41, 3.11)		3.91	(-2.71, 10.52)	
August	0.65	(-1.64, 2.93)		-0.70	(-6.87, 5.46)	
September	0.09	(-2.14, 2.31)		1.74	(-4.76, 8.25)	
October	0.44	(-1.76, 2.63)		1.15	(-5.69, 7.98)	
November	0.60	(-1.65, 2.85)		2.21	(-4.10, 8.53)	
December	0.69	(-1.57, 2.96)		5.16	(-1.13, 11.46)	
Questionnaire received while patient still in hospital	0.087	(-1.50, 1.67)	0.91	N/A		

Coef., coefficient.

[a] Two family members returned questionnaires but did not complete any of the 24 FS-ICU items – responses were not imputed for these family members.

Table S6 Sensitivity analyses –candidate determinants for the multivariable multilevel models for the family satisfaction in the intensive care unit

Candidate determinants	Justification inclusion/exclusion	Approach to modelling
**Family member level**		
Education level	It was not considered in the multivariable models due to higher than expected proportions of both "Not stated" (17%) and "Other" (21%) responses, suggesting a lack of comprehension of the categorisation used.	
Distance from home to hospital	No significant after adjusting for other variables in the model. It was dropped.	
Family member age, years	Controlling effect	Categorical (<30;30-39;40-49;50-59;60-69;70-79;80+)
Family member sex	Controlling effect	Categorical (male; female)
Family member ethnicity	Statistically significant in univariable	Categorical (white; non-white)
Next-of-kin/lives with patient	There was a strong multicollinearity between relationship to the patient and the other key variables of next-of-kin and lives with patient.	Categorical (lives with patient; Next-of-kin, does not live with patient; Not next-of-kin, does not live with patient)
Frequent visitor	Statistically significant in univariable	Binary (yes; no)
**Patient level**		
Patient ethnicity	It was not carried forward to the multivariable models due to collinearity with family member ethnicity.	
Patient age	Controlling effect	Continuous(linear)
Patient sex	Controlling effect	Categorical (male; female)
Dependency	Controlling effect	Categorical (none; minor or major; total)
Surgical status (vs non-surgical)	Controlling effect	Categorical (non-surgical; planned elective/scheduled; unplanned)
ICNARC Physiology Score	Statistically significant in univariable	Continuous(linear)
ICU length of stay (days)		Continuous(linear)
Organ support received in the ICU and duration (calendar days)	Once included in the multivariable model for	

of organ support among those receiving the support	survivors, only advanced respiratory support remained significant.	
Advanced respiratory support	It was found to be preferable to alternative variable of the duration of advanced respiratory support, which was correlated with ICU length of stay.	Binary (yes; no)
haematological malignancy	No significant after adjusting for other variables in the model. It was dropped.	
**ICU/hospital level**		
Hospital type	Controlling effect	Categorical (non-university; university; university affiliated)
Number of ICU beds	Controlling effect	Continuous(linear)

Table S7 Multivariable multilevel models for the satisfaction with care domain score

**Variables**	**Family members of ICU survivors [N=6,143[a]]**			**Family members of ICU non-survivors [N=869[a]]**		
	Coef.	95% CI	p-value	Coef.	95% CI	p-value
Fixed effects – family member level						
Constant	71.45	(66.67, 76.22)		55.29	(41.76, 68.82)	
Family member age, years (vs <30)			0.001			0.16
30-39	2.60	(0.81, 4.38)		2.50	(-1.97, 6.97)	
40-49	2.73	(1.16, 4.31)		4.31	(0.09, 8.54)	
50-59	2.91	(1.36, 4.44)		4.99	(0.93, 9.04)	
60-69	2.67	(1.08, 4.26)		4.89	(0.54, 9.23)	
70-79	2.66	(0.90, 4.41)		5.91	(0.88, 10.94)	
80+	-0.17	(-2.76, 2.41)		1.85	(-4.51, 8.21)	
Family member sex – female (vs male)	0.42	(-0.35, 1.20)	0.29	0.22	(-1.81, 2.25)	0.83
Family member ethnicity – white (vs non-white)	3.87	(1.77, 5.97)	<0.001	6.99	(0.19, 13.81)	0.044
Next-of-kin/lives with patient (vs lives with patient)			<0.001			0.15
Next-of-kin, does not live with patient	-1.14	(-2.26, -0.02)		0.95	(-2.39, 4.29)	
Not next-of-kin, does not live with patient	-2.44	(-3.32, -1.55)		-1.58	(-4.11, 0.94)	
Frequent visitor	2.49	(1.52, 3.46)	<0.001	1.49	(-1.27, 4.25)	0.29
Fixed effects – patient level						
Patient age (per 10 years)	0.03	(-0.25, 0.31)	0.83	1.21	(0.16, 2.26)	0.024
Patient sex – female (vs male)	0.06	(-0.85, 0.98)	0.87	1.85	(-0.79, 4.5)	0.17
Dependency (vs none)			0.006			0.68
Minor or major	-0.74	(-1.96, 0.46)		-0.94	(-3.98, 2.09)	
Total	-6.77	(-11.18, -2.36)		3.62	(-8.71, 15.95)	
Surgical status (vs non-surgical)			0.68			0.47
Planned elective/scheduled	-0.62	(-2.04, 0.78)		-4.85	(-12.71, 2.99)	
Unplanned	-0.15	(-1.27, 0.95)		-0.57	(-4.29, 3.13)	
ICNARC Physiology Score (per point)	0.14	(0.07, 0.21)	<0.001	0.14	(-0.03, 0.30)	0.10
ICU length of stay (per day)	-0.02	(-0.06, 0.02)	0.39	-0.30	(-0.45, -0.15)	<0.001
Advanced respiratory support	2.74	(1.66, 3.82)	<0.001			
Fixed effects – ICU/hospital level						
Hospital type (vs non-university)			0.51			0.58

University	0.94	(-3.58, 5.47)		-1.48	(-7.8 , 4.84)	
University affiliated	1.92	(-1.34, 5.19)		1.79	(-2.75, 6.34)	
Number of ICU beds (per bed)	-0.01	(-0.24, 0.23)	0.96	0.24	(-0.12, 0.59)	0.19
Random effects – SD (SE)						
Between ICUs	2.98	(0.60)		3.25	(1.11)	
Within ICUs between patients	9.76	(0.28)		10.47	(0.66)	
Within patients between family members	11.96	(0.19)		11.92	(0.42)	

Coef, coefficient; SE, standard error.

[a] Five patients were missing age group on both the questionnaire and web portal – due to the very small amount of missing data in this key variable, these missing values were not imputed.

Table S8 Multivariable multilevel models for the satisfaction with information domain score

Variables	Family members of ICU survivors [N=6,143[a]]			Family members of ICU non-survivors [N=869[a]]		
	Coef.	95% CI	p-value	Coef.	95% CI	p-value
Fixed effects – family member level						
Constant	66.07	(59.78, 72.21)		55.86	(39.34, 72.38)	
Family member age, years (vs <30)			0.63			0.28
30-39	0.28	(-2.22, 2.79)		1.23	(-4.92, 7.39)	
40-49	0.00	(-2.21, 2.21)		1.88	(-3.92, 7.68)	
50-59	0.55	(-1.62, 2.72)		2.88	(-2.70, 8.48)	
60-69	-0.1	(-2.35, 2.14)		4.24	(-1.71, 10.2)	
70-79	-0.41	(-2.89, 2.08)		6.43	(-0.45, 13.31)	
80+	-2.67	(-6.35, 1.01)		-1.96	(-10.71, 6.79)	
Family member sex – female (vs male)	0.20	(-0.89, 1.30)	0.72	1.01	(-1.81, 3.82)	0.49
Family member ethnicity – white (vs non-white)	4.73	(1.78, 7.68)	0.002	9.34	(0.47, 18.21)	0.039
Next-of-kin/lives with patient (vs lives with patient)			<0.001			0.38
Next-of-kin, does not live with patient	-2.39	(-3.97, 0.81)		1.43	(-3.09, 5.95)	
Not next-of-kin, does not live with patient	-2.57	(-3.83, 1.31)		-1.21	(-4.69, 2.28)	
Frequent visitor	2.11	(0.74, 3.48)	0.002	0.44	(-3.33, 4.22)	0.82
Fixed effects – patient level						
Patient age (per 10 years)	-0.22	(-0.61, 0.18)	0.28	0.92	(-0.43, 2.27)	0.18
Patient sex – female (vs male)	0.32	(-0.98, 1.62)	0.63	1.93	(-1.48, 5.35)	0.27
Dependency (vs none)			0.61			0.51
Minor or major	-0.49	(-2.2, 1.2)		-0.28	(-4.11, 3.53)	
Total	-2.69	(-8.92, 3.52)		9.15	(-6.57, 24.87)	
Surgical status (vs non-surgical)			0.88			0.84
Planned elective/scheduled	-0.32	(-2.32, 1.66)		-0.88	(-10.97, 9.21)	
Unplanned	0.23	(-1.33, 1.80)		-1.4	(-6.16, 3.36)	
ICNARC Physiology Score (per point)	0.23	(0.13, 0.33)	<0.001	0.15	(-0.04, 0.36)	0.13
ICU length of stay (per day)	-0.05	(-0.11, 0.01)	0.14	-0.43	(-0.62, -0.24)	<0.001
Advanced respiratory support	3.34	(1.83, 4.85)	<0.001	--		
Fixed effects – ICU/hospital level						
Hospital type (vs non-university)			0.45			0.58

University	1.69	(-3.71, 7.08)		0.35	(-6.42, 7.13)	
University affiliated	2.48	(-1.42, 6.40)		2.53	(-2.32, 7.39)	
Number of ICU beds (per bed)	-0.03	(-0.31, 0.24)	0.81	0.21	(-0.17, 0.61)	0.27
Random effects – SD (SE)						
Between ICUs	3.48	(0.73)		2.81	(1.37)	
Within ICUs between patients	13.64	(0.41)		12.38	(0.97)	
Within patients between family members	16.88	(0.27)		17.02	(0.60)	

Coef, coefficient; SE, standard error.

[a] Five patients were missing age group on both the questionnaire and web portal – due to the very small amount of missing data in this key variable, these missing values were not imputed.

Table S9 Multivariable multilevel models for the satisfaction with the decision-making process domain score

**Variables**	**Family members of ICU survivors [N=6,143[a]]**			**Family members of ICU non-survivors [N=869[a]]**		
	Coef.	95% CI	p-value	Coef.	95% CI	p-value
Fixed effects – family member level						
Constant	61.65	(55.17, 68.14)		39.62	(20.14, 59.09)	
Family member age, years (vs <30)			0.061			0.40
30-39	1.66	(-1.63, 4.95)		1.37	(-5.35, 8.10)	
40-49	0.02	(-2.76, 2.82)		2.73	(-3.47, 8.95)	
50-59	0.52	(-2.21, 3.25)		3.34	(-2.61, 9.31)	
60-69	-1.43	(-4.48, 1.61)		3.35	(-3.05, 9.77)	
70-79	-1.09	(-4.32, 2.13)		6.25	(-1.36, 13.88)	
80+	-3.87	(-8.43, 0.69)		-3.13	(-12.88, 6.61)	
Family member sex – female (vs male)	-0.18	(-1.42, 1.04)	0.77	1.66	(-1.37, 4.71)	0.28
Family member ethnicity – white (vs non-white)	0.81	(-2.67, 4.30)	0.65	6.46	(-4.24, 17.15)	0.24
Next-of-kin/lives with patient (vs lives with patient)			0.10			0.86
Next-of-kin, does not live with patient	-0.93	(-2.93, 1.05)		1.39	(-3.49, 6.28)	
Not next-of-kin, does not live with patient	-1.65	(-3.22, 0.07)		0.48	(-3.49 , 4.46)	
Frequent visitor	5.31	(3.38, 7.23)	<0.001	3.84	(-0.21, 7.91)	0.063
Fixed effects – patient level						
Patient age (per 10 years)	0.26	(-0.20, 0.73)	0.27	2.19	(0.61, 3.78)	0.007
Patient sex – female (vs male)	0.79	(-0.84, 2.43)	0.34	1.29	(-2.67, 5.26)	0.52
Dependency (vs none)			0.44			0.47
Minor or major	1.34	(-0.74, 3.43)		2.91	(-1.48, 7.29)	
Total	0.11	(-7.42, 7.64)		4.27	(-17.36, 25.91)	
Surgical status (vs non-surgical)			0.25			0.68
Planned elective/scheduled	-1.83	(-4.35, 0.68)		-1.09	(-12.59, 10.41)	
Unplanned	-1.35	(-3.41, 0.71)		2.35	(-3.20, 7.91)	
ICNARC Physiology Score (per point)	0.12	(0.01, 0.24)	0.040	0.19	(-0.04, 0.44)	0.12
ICU length of stay (per day)	0.03	(-0.04, 0.11)	0.39	-0.17	(-0.39, 0.03)	0.11
Advanced respiratory support	3.03	(1.08, 4.97)	0.002	--		
Fixed effects – ICU/hospital level						

Hospital type (vs non-university)			0.50			0.55
University	-0.41	(-4.27, 3.46)		-4.44	(-12.41, 3.53)	
University affiliated	1.51	(-1.37, 4.39)		-0.86	(-6.56, 4.83)	
Number of ICU beds (per bed)	0.02	(-0.19, 0.23)	0.85	0.47	(0.02, 0.93)	0.042
Random effects – SD (SE)						
Between ICUs	2.06	(0.66)		3.33	(1.50)	
Within ICUs between patients	17.24	(0.50)		15.84	(1.06)	
Within patients between family members	17.02	(0.40)		16.81	(0.66)	

Coef, coefficient; SE, standard error.

[a] Five patients were missing age group on both the questionnaire and web portal – due to the very small amount of missing data in this key variable, these missing values were not imputed.

Figure S2 Variation across ICUs in the mean: satisfaction with care domain score (A) before and (B) after adjustment; satisfaction with information domain score (C) before and (D) after adjustment; and satisfaction with the decision-making process domain score (E) before and (F) after adjustment

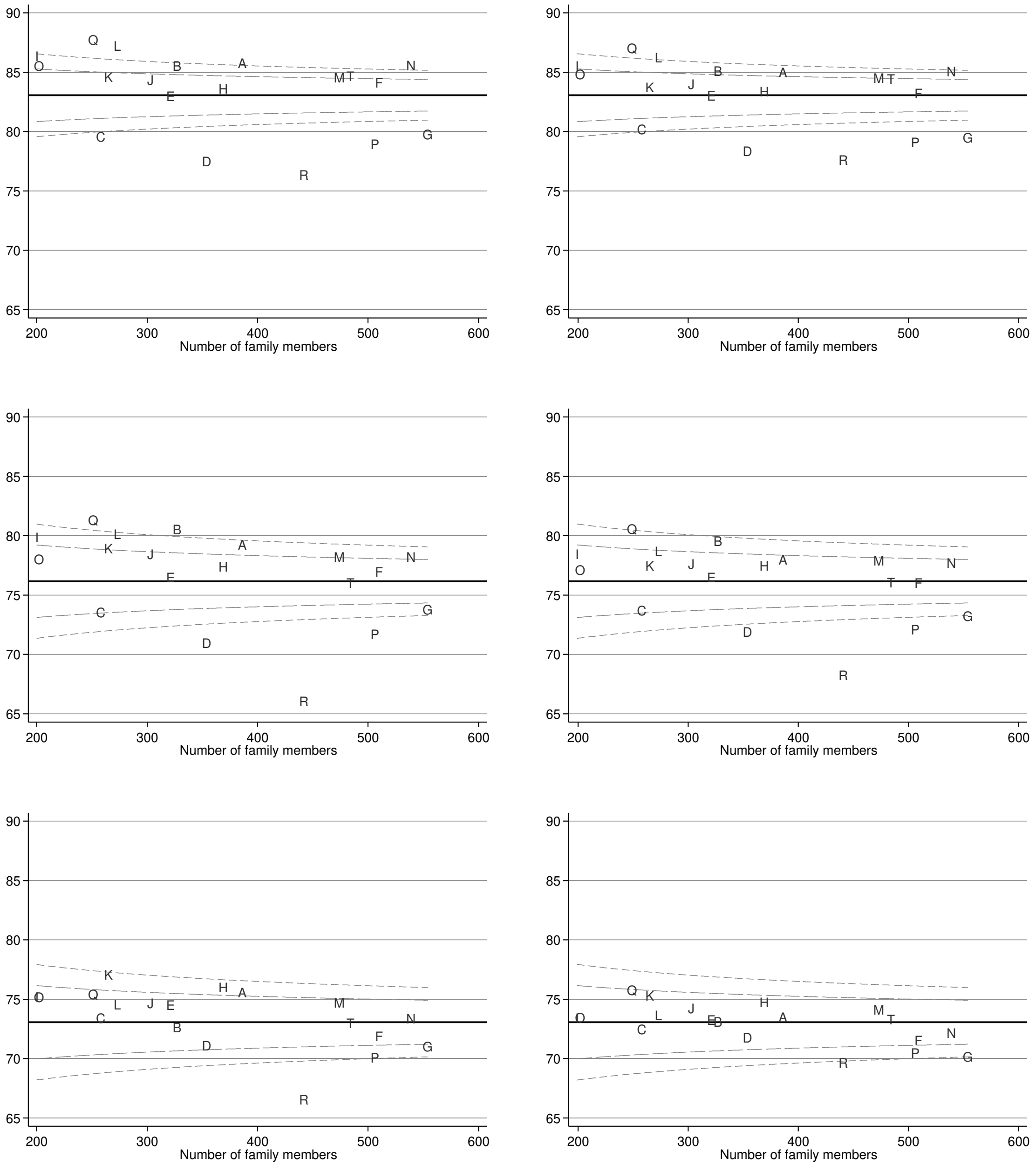

Table S10 Sensitivity analyses – alternative approach to handling missing data (family members of ICU survivors)

Variables	Complete case [N=2,351]			Traditional approach [N=5,756]		
	Coef.	SE	p-value	Coef.	SE	p-value
Constant	72.60	3.18		70.35	2.49	
Family member age, years (vs <30)			0.61			0.20
30-39	0.13	1.40		1.47	0.97	
40-49	0.85	1.22		1.41	0.86	
50-59	0.66	1.20		1.58	0.84	
60-69	0.65	1.30		1.47	0.88	
70-79	0.77	1.47		1.69	0.98	
80+	-3.06	2.26		-1.22	1.50	
Family member sex – female (vs male)	0.94	0.60	0.12	0.21	0.43	0.63
Family member ethnicity – white (vs non-white)	7.58	1.58	<0.001	3.99	1.16	0.001
Next-of-kin/lives with patient (vs lives with patient)			0.071			0.002
Next-of-kin, does not live with patient	-1.69	0.85		-1.36	0.61	
Not next-of-kin, does not live with patient	-1.42	0.72		-1.70	0.50	
Frequent visitor	1.18	0.82	0.15	2.21	0.55	<0.001
Patient age (per 10 years)	-0.09	0.22	0.67	-0.07	0.15	0.64
Patient sex – female (vs male)	-1.20	0.73	0.10	0.13	0.52	0.79
Dependency (vs none)			0.70			0.45
Minor or major	-0.44	0.92		-0.19	0.68	
Total	-2.19	2.98		-3.14	2.51	
Surgical status (vs non-surgical)			0.056			0.47
Planned elective/scheduled	-3.11	1.30		-0.93	0.80	
Unplanned	-0.44	0.88		0.02	0.62	
ICNARC Physiology Score (per point)	0.08	0.05	0.14	0.15	0.04	<0.001
ICU length of stay (per day)	-0.04	0.03	0.28	-0.04	0.03	0.17
Advanced respiratory support	1.39	0.87	0.11	2.40	0.60	<0.001
Hospital type (vs non-university)			0.42			0.34
University	0.56	2.36		1.45	2.22	

University affiliated	2.24	1.72		2.34	1.61	
Number of ICU beds (per bed)	0.07	0.12	0.59	-0.02	0.11	0.83

Coef., coefficient; SE, standard error.

Table S11 Sensitivity analyses – alternative approaches to handling missing data (family members of ICU non-survivors)

Variables	Complete case [N=547]			Traditional approach [N=851]		
	Coef.	SE	p-value	Coef.	SE	p-value
Constant	54.46	7.72		56.28	6.80	
Family member age, years (vs <30)			0.17			0.086
30-39	4.38	3.01		3.14	2.44	
40-49	7.51	2.75		4.87	2.31	
50-59	6.19	2.62		4.50	2.22	
60-69	7.41	2.85		5.94	2.37	
70-79	6.99	3.69		7.07	2.82	
80+	7.52	4.41		0.32	3.61	
Family member sex – female (vs male)	-0.02	1.43	0.99	0.40	1.11	0.72
Family member ethnicity – white (vs non-white)	9.64	4.21	0.022	7.47	3.58	0.037
Next-of-kin/lives with patient (vs lives with patient)			0.97			0.38
Next-of-kin, does not live with patient	0.13	2.20		1.27	1.82	
Not next-of-kin, does not live with patient	-0.32	1.81		-0.82	1.40	
Frequent visitor	1.32	1.96	0.50	0.99	1.51	0.51
Patient age (per 10 years)	0.69	0.66	0.29	1.09	0.55	0.048
Patient sex – female (vs male)	1.56	1.69	0.36	2.02	1.41	0.15
Dependency (vs none)			0.47			0.66
Minor or major	-0.61	1.86		-0.32	1.58	
Total	8.53	7.42		5.59	6.45	
Surgical status (vs non-surgical)			0.84			0.51
Planned elective/scheduled	-0.33	5.61		-4.86	4.22	
Unplanned	-1.38	2.33		-0.44	1.95	
ICNARC Physiology Score (per point)	0.24	0.10	0.022	0.18	0.09	0.041
ICU length of stay (per day)	-0.27	0.09	0.003	-0.33	0.08	<0.001
Hospital type (vs non-university)			0.83			0.77
University	-1.15	3.20		-0.11	3.01	
University affiliated	0.84	2.29		1.49	2.17	

Number of ICU beds (per bed)	0.25	0.19	0.17	0.21	0.17	0.23

Coef., coefficient; SE, standard error.
