# Supplementary material for: Family satisfaction with critical care in the UK: a multicentre cohort study
Source: BMJ Open. 2019 Aug 20;9(8):e028956. doi: 10.1136/bmjopen-2019-028956 (PMC6707657; doi:10.1136/bmjopen-2019-028956)
Supplement: Author's manuscript [file bmjopen-2019-028956.draft_revisions.pdf]

BMJ Open is committed to open peer review. As part of this commitment we make the peer review history of every article we publish publicly available.

When an article is published we post the peer reviewers' comments and the authors' responses online. We also post the versions of the paper that were used during peer review. These are the versions that the peer review comments apply to.

The versions of the paper that follow are the versions that were submitted during the peer review process. They are not the versions of record or the final published versions. They should not be cited or distributed as the published version of this manuscript.

BMJ Open is an open access journal and the full, final, typeset and author-corrected version of record of the manuscript is available on our site with no access controls, subscription charges or pay-per-view fees (http://bmjopen.bmj.com).

If you have any questions on BMJ Open's open peer review process please email info.bmjopen@bmj.com

BMJ Open

Family satisfaction with critical care in the United Kingdom: a multi-centre cohort study

Journal:	*BMJ Open*
Manuscript ID	bmjopen-2019-028956
Article Type:	Research
Date Submitted by the Author:	11-Jan-2019
Complete List of Authors:	Ferrando, Paloma; Intensive Care National Audit and Research Centre,<br>Gould, Doug; Intensive Care National Audit and Research Centre<br>Walmsley, Emma; Intensive Care National Audit and Research Centre<br>Richards-Belle, Alvin; Intensive Care National Audit and Research Centre<br>Canter, Ruth; Intensive Care National Audit and Research Centre<br>Saunders, Steven; Intensive Care National Audit and Research Centre<br>Harrison, David; Intensive Care National Audit and Research Centre<br>Harvey, Sheila; London School of Hygiene & Tropical Medicine, Global Health and Development<br>Heyland, Daren; Kingston General Hospital, Clinical Evaluation Research Unit; Queens University, Department of Medicine<br>Hinton, Lisa; University of Oxford, Health Experiences Research Group, Nuffield Department of Primary Care Health Sciencs<br>McColl, Elaine; Newcastle Clinical Trials Unit<br>Richardson, Annette; Freeman Hospital, Perioperative and Critical Care<br>Richardson, Michael<br>Wright, Stephen; Freeman Hospital, Perioperative and Critical Care<br>Rowan, Kathryn; Intensive Care National Audit and Research Centre
Keywords:	Adult intensive & critical care < ANAESTHETICS, Quality in health care < HEALTH SERVICES ADMINISTRATION & MANAGEMENT, Family satisfaction

SCHOLARONE™
Manuscripts

Family satisfaction with critical care in the United Kingdom: a multi-centre cohort study

Authors

Paloma Ferrando-Vivas, *statistician*[1], Doug W Gould, *senior researcher*[1], Emma Walmsley, *study co-ordinator*[1], Alvin Richards-Belle, *trial manager*[1], Ruth R Canter, *research assistant*[1], Steven Saunders, *research administrator*[1], David A Harrison, *head statistician*[1], Sheila E Harvey, *Associate Professor*[2], Daren K Heyland, *director of the Kingston General Hospital Clinical Evaluation Research Unit and professor of medicine and epidemiology*[3], Lisa Hinton, *senior qualitative researcher*[4], Elaine McColl, *director of Newcastle CTU and professor of health services research*[5], Annette Richardson, *nurse consultant in critical care*[6], Michael Richardson, *patient public representative*[7], Stephen E Wright, *consultant in anaesthesia and intensive care*[6], Kathryn M Rowan, *director of scientific & strategic development*[1].

[1]Intensive Care National Audit & Research Centre (ICNARC), London, United Kingdom, WC1V 6AZ

[2]London School of Hygiene & Tropical Medicine, Global Health and Development, London, United Kingdom

[3]Kingston General Hospital, Clinical Evaluation Research Unit, Kingston, Canada and Queen's University, Department of Medicine, School of Medicine, Canada.

[4] University of Oxford, Health Experiences Research Group, Nuffield Department of Primary Care Health Sciences, Oxford, United Kingdom.

[5]Newcastle Clinical Trials Unit, Newcastle University, Newcastle upon Tyne, United Kingdom.

[6]Freeman Hospital, Perioperative and Critical Care, Newcastle Upon Tyne Hospitals NHS Foundation Trust, Newcastle upon Tyne, United Kingdom.

[7]Newcastle Upon Tyne, United Kingdom.

Corresponding author:

Kathryn M. Rowan (ORCid iD: 0000 0001 8217 5602), Director of Scientific & Strategic Development, Intensive Care National Audit & Research Centre, Napier House, 24 High Holborn, London WC1V 6AZ

Tel: 020 7831 6878

Fax: 020 7831 6879

e-mail: Kathy.rowan@icnarc.org

For peer review only - http://bmjopen.bmj.com/site/about/guidelines.xhtml

Abstract

Objective: To assess family satisfaction with intensive care units (ICUs) in the United Kingdom using the Family Satisfaction in the Intensive Care Unit 24-item questionnaire (FS-ICU-24), and to investigate how characteristics of patients and their family members impact on family satisfaction.

Design: Prospective cohort study nested within a national clinic audit database.

Setting: Stratified, random sample of 20 adult general ICUs participating in the Intensive Care Audit & Research Centre (ICNARC) Case Mix Programme.

Participants: Family members of patients staying at least 24 hours in ICU were recruited between May 2013 and June 2014

Interventions: Consenting family members were sent a postal questionnaire three weeks after the patient died or was discharged from ICU. Up to four family members were recruited per patient.

Main outcome measures: Family satisfaction measured using the UK FS-ICU-24 questionnaire.

Main Results: 12,346 family members of 6380 patients were recruited and 7173 (58%) family members of 4615 patients returned a completed questionnaire. Overall and domain specific family satisfaction scores were high (mean overall family satisfaction 80, satisfaction with care 83, satisfaction with information 76, and satisfaction with decision-making 73 out of 100) but varied significantly across adult general ICUs studied and by whether the patient survived ICU. For family members of ICU survivors, characteristics of both family member (age, ethnicity, relationship to patient (next-of-kin and/or lived with patient) and visit frequency) and the patient (acute severity of illness and receipt of invasive mechanical ventilation) were significant determinants of family satisfaction, whereas, for family members of ICU non-survivors, only patient characteristics (age, acute severity of illness, and duration of stay) were significant.

Conclusions: Overall family satisfaction in UK adult general ICUs was high but varied significantly. Adjustment for differences in family member/patient characteristics is important to avoid falsely identifying ICUs as outliers.

Study registration: ISRCTN 47363549

For peer review only

For peer review only - http://bmjopen.bmj.com/site/about/guidelines.xhtml

Keywords: critical care; intensive care units; personal satisfaction; family; quality of care; communication

Strengths and limitations of this study

- This is the largest study assessing family satisfaction with ICU care.
- Unbiased selection and stratification of participating units ensured geographical spread (north, south, east, and west England, Wales and Northern Ireland), hospital type (university or non-university) and ICUs of different sizes (large or small – based on number of beds) that recruited for one year to avoid bias from seasonal variation.
- Nesting our study within the Case Mix Programme national clinical audit was efficient and allowed for linkage of family members' to patient data.
- The same mode and timing of delivery of the FS-ICU-24 was employed for family members of ICU survivors and non-survivors, avoiding potential sampling bias and allowing for meaningful comparisons between these groups.
- Despite our very large sample size, we achieved a modest response rate (58%), which was in line with previous published studies.

For peer review only

For peer review only - http://bmjopen.bmj.com/site/about/guidelines.xhtml

Introduction

Humanity of health care, often measured as patient experience, is increasingly seen as one of the three pillars of quality, alongside effectiveness and equity. Eliciting the views and experiences of patients is now seen as essential in delivering a high quality service (1). However, given that approximately 20% of patients admitted to intensive care units (ICUs) die and survivors are often unable to recall their experiences, measuring patient experience in ICU has particular challenges. For this reason, measures of family experience have been developed to help understand the humanity of ICU care.

The most widely validated measure of family experience is the Family Satisfaction in the Intensive Care Unit questionnaire (FS-ICU). This describes satisfaction, overall and in two domains – *satisfaction with care* and *satisfaction with decision making* (2-4). The overall aim of the Family-Reported Experiences Evaluation (FREE) study was to inform the potential routine use of the FS-ICU-24 questionnaire for quality improvement in adult general ICUs in the UK.

This paper reports the results of a large, prospective, multicentre, cohort study describing family satisfaction with ICU care in the UK, investigates how characteristics of patients and their family members impact on family satisfaction, and explores if family satisfaction, varies across ICUs, before and after adjustment for family member and patient characteristics identified as being associated with family satisfaction.

Methods

This large, prospective, multicentre cohort study was nested in the Intensive Care National Audit & Research Centre (ICNARC) Case Mix Programme (CMP) – the national clinical audit of adult general ICUs in England, Wales and Northern Ireland. A stratified sample of 20 ICUs were selected to ensure geographical spread (north, south, east, and west England, Wales and Northern Ireland), hospital type (university or non-university) and ICUs of different sizes (large or small – based on number of beds) and recruited for one year to avoid bias from seasonal variation. The study was reviewed and approved by the National Research Ethics Service Committee South Central - Berkshire B (reference 13/SC/0037) and was registered prospectively (ISRCTN47363549).

Patient and Public Involvement

Engagement with patient and their family members was vital to ensuring the successful delivery of the FREE study. A former critical care patient and a family member of a former critical care patient

For peer review only - http://bmjopen.bmj.com/site/about/guidelines.xhtml

were co-investigators on the FREE study and contributed to all aspects of the study including: design; conduct; management; analysis; interpretation of results; and dissemination as members of the study management group. Additionally, the study steering committee included patient and family members.

Recruitment and follow-up

Recruitment and follow-up of family members have been described in detail elsewhere (5). Briefly, a ‘family member’ was defined as any person with close familial, social or emotional relationship to the patient and was not restricted solely to next-of-kin. Up to four family members of patients who spent ≥24 hours in ICU were eligible to participate if they met the following criteria: aged ≥18 years; had physically visited the patient’s bedside at least once after the first 24 hours; had a UK postal address; and had not already been recruited into the study.

Patients were followed-up to ICU discharge. Approximately three weeks after the patient had either been discharged from or died in the ICU, a questionnaire pack was mailed to their recruited and consented family member(s) direct from the ICNARC Clinical Trials Unit. Data from completed questionnaires were entered centrally onto a secure database. Quality checking of entered data was conducted and, for a 20% random sample, accuracy was verified. All fields in the database with missing data were verified against the paper questionnaires.

Statistical analysis

Item responses were rescaled and, where relevant, reversed, according to the developer’s rules, so that each response was on a scale from 0 (least satisfied) to 100 (most satisfied) (4). Recent work from our group (6) established the construct validity of the FS-ICU 24-item questionnaire (FS-ICU-24) was improved by using three domains (splitting the *satisfaction with decision making* domain into two – *satisfaction with information* and *satisfaction with decision making process*). Overall family satisfaction score and three domain scores were calculated by averaging the item responses for the relevant items.

Family member and patient characteristics were described by mean and standard deviation (SD), median and quartiles, or number and percentage stratified by the patient outcome (alive/dead). Variation in family satisfaction was analysed across the following factors: patient; family member; ICU/hospital (hospital teaching status and number of beds in the ICU); and other contextual. These factors were then explored using univariable and multivariable multilevel linear regression models (7) with a primary outcome of the overall family satisfaction score. In secondary analyses, separate models were fitted for the three individual domains of family satisfaction. Separate models were

For peer review only - http://bmjopen.bmj.com/site/about/guidelines.xhtml

fitted for family members of ICU survivors and non-survivors. All analyses were conducted in Stata/SE Version 13.0 (StataCorp, College Station, TX).

Variation in family satisfaction across ICUs was assessed graphically using funnel plots, which plot the average family satisfaction score for each critical care unit against the number of family members returning questionnaires. Control limits placed at 2 and 3 SDs around the overall mean indicate the regions of the funnel within which we would expect 95% and 99.8% of points to lie if all variation was due to chance (8).

Due to the natural structure of the data and the planned analysis multilevel multiple imputation (MLMI) was used to complete non- and partial responses for outcomes and family member characteristics. Data were imputed using REALCOM-Impute, an MLwiN 2.15 macro that generates imputations for hierarchical data (9). To test whether our findings were influenced by using imputed data, we also conducted sensitivity analyses using a traditional approach to scoring the FS-ICU-24 by including only responders with ≥60% of items completed.

Results

Of the 210 adult, general ICUs participating in the CMP, 142 (67.6%) expressed an interest in participating and the 20 ICUs were selected using stratified, random sampling. The characteristics and outcomes of all admissions to the study ICUs were similar to admissions to all ICUs in the CMP during the same period (Supplementary Table S1).

Between 28 May 2013 and 30 June 2014, 18,757 patients were admitted to the 20 ICUs, of which 12,730 patients stayed at least 24 hours in the ICU. From these, 12,346 family members of 6380 patients were recruited. Fully or partially completed questionnaires were returned by 7173 family members of 4615 patients. Family members of patients for whom no CMP data were available were not included, so finally, 7019 were included in the final analysis (Supplementary Figure S1).

Response rates varied by family member characteristics, including; age, gender, ethnicity, level of deprivation (based on residential postcode), level of education, and relationship with the patient. Family members documented in ICU records as next-of-kin were more likely to complete the questionnaire than those who were not, whilst family members for whom English was their first language were more likely to complete the questionnaire than those for whom it was not (Table S2).

A detailed description of the inclusion process, response rates and responders' characteristics has been reported in Family Reported Experiences Evaluation (FREE) study (5). Comparisons of family

For peer review only - http://bmjopen.bmj.com/site/about/guidelines.xhtml

member and patient characteristics for ICU survivors and non-survivors are presented in Table 1 and Table 2, respectively.

Table 1 Family member characteristics stratified by the patient's ICU outcome

**Family member characteristics**	**All Family members [N=7,019]**	**Family members of ICU survivors [N=6,149]**	**Family members of ICU non-survivors [N=870]**
Age, mean (SD)	54 (15.1)	54 (15.0)	52 (15.2)
Age group, n (%)			
<30	507 (7.5)	439 (7.4)	68 (8.0)
30-39	701 (10.3)	595 (10.0)	106 (12.5)
40-49	1,423 (21.0)	1,245 (21.0)	178 (21.0)
50-59	1,614 (23.8)	1,406 (23.7)	208 (24.6)
60-69	1,507 (22.2)	1,334 (22.5)	173 (20.4)
70-79	827 (12.2)	747 (12.6)	80 (9.5)
80+	204 (3.0)	171 (2.9)	33 (3.9)
Sex, n (%)			
Male	2,327 (33.5)	2,052 (33.7)	275 (31.9)
Female	4,622 (66.5)	4,034 (66.3)	588 (68.1)
Ethnicity, n (%)			
White	6,555 (94.0)	5,738 (93.9)	817 (94.6)
Asian	138 (2.0)	114 (1.9)	24 (2.8)
Black	54 (0.8)	50 (0.8)	4 (0.5)
Mixed ethnicity or other ethnic group	88 (1.3)	84 (1.4)	4 (0.5)
Not stated	139 (2.0)	124 (2.0)	15 (1.7)
Relationship to patient, n (%) ("I am the patient's…")			
Partner	2,096 (29.9)	1,891 (30.8)	205 (23.6)
Child	654 (9.3)	1,893 (30.8)	346 (39.8)
Parent	2,239 (31.9)	622 (10.1)	32 (3.7)
Sibling	704 (10.0)	624 (10.1)	80 (9.2)
Other relative	969 (13.8)	799 (13.0)	170 (19.5)
Other non-relative	356 (5.1)	319 (5.2)	37 (4.3)
Next-of-kin, n (%)	3,520 (50.2)	3,153 (51.4)	367 (42.3)
Lives with patient, n (%)	2,559 (36.5)	2,311 (37.6)	248 (28.5)
Highest level of education, n (%)			
NVQ level 1 or 2	1,683 (28.9)	1,465 (28.9)	218 (29.1)
NVQ level 3	1,123 (19.3)	989 (19.5)	134 (17.9)
NVQ level 4 or 5	1,769 (30.4)	1,537 (30.3)	232 (31.0)
Other	1,244 (21.4)	1,080 (21.3)	164 (21.9)
Quintile of deprivation, n (%)			
1 (least deprived)	1,190 (17.1)	1,164 (19.9)	159 (19.4)
2	1,405 (20.2)	1,281 (21.9)	181 (22.1)
3	1,488 (21.4)	1,238 (21.1)	181 (22.1)

For peer review only

For peer review only - http://bmjopen.bmj.com/site/about/guidelines.xhtml

4	1,488 (21.4)	1,189 (20.3)	169 (20.7)
5 (most deprived)	1,391 (20.0)	989 (16.9)	128 (15.6)
Distance (km) from home to hospital, median (IQR)	12.4 (5.4 33.6) [6,714]	12 (6, 34)	12 (5, 33)
Previous experience of ICU as a family member, n (%)	1,841 (26.6)	1,641 (27.1)	200 (23.3)
Frequent visitor, n (%)	5,403 (78.9)	4,713 (78.6)	690 (81.2)

Table 2 Patient characteristics stratified by ICU outcome

Patient characteristics	All patients [N=4,506]	ICU survivors [N=4,007]	ICU non-survivors [N=499]
Age, mean (SD)	63 (17.0)	63 (17.3)	68 (13.2)
Age group, n (%)			
<30	254 (5.6)	246 (6.1)	8 (1.6)
30-39	232 (5.1)	223 (5.6)	9 (1.8)
40-49	412 (9.1)	384 (9.6)	28 (5.6)
50-59	643 (14.3)	586 (14.6)	57 (11.4)
60-69	1,100 (24.4)	966 (24.1)	134 (26.9)
70-79	1,159 (25.7)	1,003 (25.0)	156 (31.3)
80+	706 (15.7)	599 (14.9)	107 (21.4)
Sex, n (%)			
Male	2,561 (56.8)	2,264 (56.5)	297 (59.5)
Female	1,945 (43.2)	1,743 (43.5)	202 (40.5)
Ethnicity, n (%)			
White	4,176 (92.7)	3,706 (92.5)	470 (94.2)
Asian or Asian British	81 (1.8)	69 (1.7)	12 (2.4)
Black or black British	42 (0.9)	39 (1.0)	3 (0.6)
Mixed ethnicity or other ethnic group	79 (1.8)	74 (1.8)	5 (1.0)
Not stated	128 (2.8)	119 (3.0)	9 (1.8)
Quintile of deprivation, n (%)			
1 (least deprived)		690 (17.4)	84 (17)
2		812 (20.4)	93 (18.8)
3		822 (20.7)	106 (21.4)
4		841 (21.2)	109 (22)
5 (most deprived)		809 (20.4)	103 (20.8)
Distance (km) from home to hospital, median (IQR)	33.1 (67.8) 9.3 (4.3 19.9) [4,475]	10 (4, 20)	8 (4, 16)
APACHE II severe co-morbidities, n (%)			
Liver	3,647 (80.9)	94 (2.3)	30 (6.0)
Renal	650 (14.4)	97 (2.4)	11 (2.2)
Respiratory	191 (4.2)	119 (3.0)	27 (5.4)
Cardiovascular	18 (0.4)	100 (2.5)	17 (3.4)

For peer review only

For peer review only - http://bmjopen.bmj.com/site/about/guidelines.xhtml

Metastatic cancer	3,647 (80.9)	110 (2.7)	11 (2.2)
Haematological malignancy	650 (14.4)	81 (2.0)	22 (4.4)
Immunocompromise	191 (4.2)	318 (7.9)	51 (10.2)
Prior dependency, n (%)			
Able to live without assistance	3,267 (72.5)	2,944 (73.5)	323 (64.7)
Minor or major assistance	1,171 (26.0)	1,004 (25.1)	167 (33.5)
Total assistance	47 (1.0)	42 (1.0)	5 (1.0)
Unknown	21 (0.5)	17 (0.4)	4 (0.8)
Surgical status *n* (%)			
Non-surgical	2,808 (62.3)	2,396 (59.8)	412 (82.6)
Planned admission following elective or scheduled surgery	702 (15.6)	686 (17.1)	16 (3.2)
Unplanned admission following surgery of any urgency	996 (22.1)	925 (23.1)	71 (14.2)
ICNARC Physiology Score, mean (SD)	18 (8.3)	18 (7.9)	26 (8.1)
APACHE II Score, mean (SD)	17 (6.3)	16 (6.1)	21 (6.2)
ICU length of stay (days), median (IQR)	4.9 (2.9 9.1)	4.8 (2.8, 9.0)	6.0 (3.6, 10.6)
Organ support received in the ICU, n (%)			
Advanced respiratory support	1,966 (43.6)	2,124 (53.0)	416 (83.4)
Advanced cardiovascular support	3,181 (70.6)	1,037 (25.9)	288 (57.7)
Renal support	3,815 (84.7)	510 (12.7)	181 (36.3)
Neurological support	3,889 (86.3)	503 (12.6)	114 (22.8)
Duration (calendar days) of organ support among those receiving the support, median (IQR)			
Advanced respiratory support	5.0 (2.0 9.0)	4 (2, 9)	6 (4, 10)
Advanced cardiovascular support	3.0 (2.0 4.0)	2 (2, 4)	3 (2, 5)
Renal support	4.0 (3.0 8.0)	4 (3, 8)	4 (3, 8)
Neurological support	3.0 (2.0 7.0)	3 (2, 7)	3 (2, 5)
Death before acute hospital discharge, n (%)	852 (19.2)	353 (8.9)	N/A

Both overall and individual domain scores revealed generally high satisfaction (Table 3), however a long tail was present indicating some questionnaires were returned with very low scores (Figure 1). Family members of ICU non-survivors had higher scores for overall satisfaction and satisfaction with the decision-making process domain than family members of ICU survivors.

For peer review only - http://bmjopen.bmj.com/site/about/guidelines.xhtml

Table 3 Overall family satisfaction score for all family members and for family members by patient outcome

Summary measures	All family members [N=7,017[a]]	Family members of ICU survivors [N=6,147[a]]	Family members of ICU non-survivors [N=870]
Overall family satisfaction score			
**Median [IQR]**	83.3 [70.4, 93.0]	82.7 [69.9, 92.7]	87.1 [74.4, 94.8]
**Mean (SD)**	79.7 (16.7)	79.3 (16.5)	82.0 (17.5)
**[95% CI]**	[79.2 - 80.1]	[78.9 - 79.8]	[80.9 - 83.2]
Satisfaction with care domain score			
**Median [IQR]**	87.5 [74.3, 96.4]	87.5 [73.6, 96.4]	88.1 [76.8, 96.4]
**Mean (SD)**	83.1 (16.0)	83.0 (15.9)	83.8 (16.9)
**[95% CI]**	[82.7 - 83.4]	[82.6 - 83.4]	[82.7 - 84.9]
Satisfaction with information domain score			
**Median [IQR]**	79.2 [66.7, 95.8]	79.2 [62.5, 95.8]	83.3 [70.8, 100.0]
**Mean (SD)**	76.2 (22.0)	75.7 (22.0)	79.6 (22.9)
**[95% CI]**	[75.7 - 76.7]	[75.1 - 76.2]	[78.1 - 81.0]
Satisfaction with the decision-making process domain score			
**Median [IQR]**	75.6 [59.3, 93.1]	75.0 [57.5, 88.8]	87.5 [68.8, 100.0]
**Mean (SD)**	73.1 (22.3)	72.1 (22.0)	79.6 (22.9)
**[95% CI]**	[72.5 - 73.6]	[71.6 - 72.7]	[78.1 - 81.1]

[a] Two family members returned questionnaires but did not complete any of the 24 FS-ICU items – responses were not imputed for these family members.

For peer review only

Univariable analyses of the association between family satisfaction and family characteristics, patient characteristics, ICU/hospital characteristics and contextual factors are shown in the Supplementary Appendix (Table S3-S5). Family member level and patient level variables that were statistically significant along with the a priori key family member/patient variables (age, sex), were carried forward to the multivariable multilevel modelling process (5). There was no evidence of differences in family satisfaction according to hospital teaching status or the number of beds in the ICU, however, these variables were retained in the multilevel multivariable models due to their controlling effect on the other coefficients in the models. A summary of the candidate considered in the models and a justification of their inclusion/exclusion is detailed in Table S6.

For peer review only - http://bmjopen.bmj.com/site/about/guidelines.xhtml

Results of the multivariable multilevel models for overall family satisfaction are shown in Table 4. Among family members of ICU survivors, there was evidence of an independent association with overall family satisfaction for: family member age group; family member ethnicity; next-of-kin/lives with patient; frequency of visits; ICNARC Physiology Score; and receipt of advanced respiratory support. Among family members of non-survivors, only the following patient factors were significant: patient age; ICNARC Physiology Score; and ICU length of stay. A priori-specified interaction terms and random slopes did not improve the fit of the models and so these terms were not retained.

Table 4 Multivariable multilevel models for overall family satisfaction score

**Variables**	**Family members of ICU survivors [N=6,143[a]]**			**Family members of ICU non-survivors [N=869[a]]**		
	Coef.	95% CI	p-value	Coef.	95% CI	p-value
Fixed effects – family member level						
Constant	68.30	(63.42, 73.17)	<0.001	55.70	(42.26, 69.14)	<0.001
Family member age, years (vs <30)			0.041			0.18
30-39	1.97	(0.11, 3.82)		2.01	(-2.64, 6.66)	
40-49	1.65	(0.02, 3.29)		3.37	(-1.01, 7.75)	
50-59	1.96	(0.35, 3.56)		4.12	(-0.09, 8.33)	
60-69	1.35	(-0.31, 3.01)		4.26	(-0.25, 8.79)	
70-79	1.32	(-0.52, 3.17)		5.92	(0.69, 11.14)	
80+	-1.34	(-4.06, 1.37)		-0.18	(-6.80, 6.43)	
Family member sex – female (vs male)	0.32	(-0.48, 1.12)	0.44	0.66	(-1.45, 2.77)	0.54
Family member ethnicity – white (vs non-white)	3.59	(1.38, 5.80)	0.001	7.12	(-0.00, 14.25)	0.050
Next-of-kin/lives with patient (vs lives with patient)			<0.001			0.26
Next-of-kin, does not live with patient	-1.39	(-2.56, -0.22)		1.08	(-2.39, 4.55)	
Not next-of-kin, does not live with patient	-2.33	(-3.26, -1.41)		-1.24	(-3.88, 1.40)	
Frequent visitor	2.83	(1.82, 3.84)	<0.001	1.53	(-1.34, 4.39)	0.30
Fixed effects – patient level						
Patient age (per 10 years)	0.01	(-0.28, 0.31)	0.93	1.18	(0.09, 2.27)	0.033
Patient sex – female (vs male)	0.26	(-0.73, 1.25)	0.61	1.92	(-0.85, 4.70)	0.17
Dependency (vs none)			0.15			0.74
Minor or major	-0.30	(-1.60, 1.00)		-0.22	(-3.36, 2.92)	
Total	-4.62	(-9.32, 0.07)		4.98	(-8.10, 18.07)	

For peer review only - http://bmjopen.bmj.com/site/about/guidelines.xhtml

Surgical status (vs non-surgical)			0.63			0.82
Planned elective/scheduled	-0.74	(-2.24, 0.77)		-2.61	(-10.77, 5.54)	
Unplanned	-0.26	(-1.46, 0.94)		-0.08	(-3.95, 3.80)	
ICNARC Physiology Score (per point)	0.16	(0.09, 0.24)	<0.001	0.17	(0.00, 0.34)	0.045
ICU length of stay (per day)	-0.02	(-0.07, 0.03)	0.44	-0.30	(-0.46, -0.15)	<0.001
Advanced respiratory support	2.96	(1.80, 4.11)	<0.001	---		
Fixed effects – ICU/hospital level						
Hospital type (vs non-university)			0.49			0.55
University	0.86	(-3.61, 5.32)		-1.51	(-7.51, 4.50)	
University affiliated	1.97	(-1.26, 5.20)		1.77	(-2.55, 6.09)	
Number of ICU beds (per bed)	-0.00	(-0.23, 0.23)	0.97	0.26	(-0.08, 0.61)	0.13
Random effects – SD (SE)						
Between ICUs	2.91	(0.60)		2.81	(1.10)	
Within ICUs between patients	10.94	(0.29)		11.16	(0.69)	
Within patients between family members	11.98	(0.21)		12.26	(0.44)	
Variance partition – percentage						
Between ICUs	3%			2%		
Between patients	44%			44%		

Coef, coefficient; SE, standard error.

[a]Five patients were missing age group on both the questionnaire and web portal – due to the very small amount of missing data in this key variable, these missing values were not imputed.

Variances at both the patient and ICU/hospital levels were statistically significant but the variance partition coefficients (VPCs) at the ICU/hospital level were small in both the null and final multilevel models (4% and 3% for ICU survivors and 2% and 2% for ICU non-survivors, respectively), which means differences in overall family satisfaction scores were mainly at the patient and family member levels. Variance at the patient level represented 44% of the total variance in overall family satisfaction in the final models for family members of both ICU survivors and ICU non-survivors.

Full results of the multivariable multilevel models for the domain scores are reported in the Supplementary Appendix (Table S7-S9).

Figure 2 shows the funnel plots for the overall family satisfaction score, before and after adjustment for family member and patient characteristics from the multivariable multilevel models. Adjusting for family member and patient characteristics reduced the variability across ICUs, resulting in fewer

For peer review only

For peer review only - http://bmjopen.bmj.com/site/about/guidelines.xhtml

ICUs outside the funnel plot control limits. Funnel plots for the individual domain scores before and after adjustment for can be found in the Supplementary Appendix (Figure S2).

Sensitivity analyses

For the multivariable multilevel modelling the direction and order of magnitude of coefficients that were significant in the models estimated using imputed data were similar to those estimated using the traditional approach to scoring partially completed questionnaires (Supplementary Appendix, Table S10 and Table S11). On average, the multiple imputation approach tended to identify larger numbers of potential outliers due to the larger sample sizes and therefore narrower funnels.

Discussion

Overall and domain specific family satisfaction measured with the UK FS-ICU-24 was high. However, we found that it varies significantly across adult general ICUs and that family members of patients who died in the ICU had higher levels of satisfaction. For family members of ICU survivors, characteristics of both family member and the patient were significant determinants of family satisfaction, whereas, for family members of ICU non-survivors, only patient characteristics were significant. Adjustment for these family member and patient characteristics reduced the variation in family satisfaction across ICUs, resulting in fewer ICUs being identified as outliers.

The overall satisfaction score was comparable with other published studies employing similar methods to administer the FS-ICU-24 (10-13). Our findings are also consistent with a study by Wall et al (14) which identified that families of ICU non-survivors were more satisfied than families of ICU survivors. Similarly, Stricker et al (15) found that increasing acute severity of illness of the patient (evaluated using the SAPS II score) was associated with increasing satisfaction on the overall family satisfaction score, however, lower satisfaction was associated with ICU-level characteristics of a written admission/discharge policy and a higher patient:nurse ratio. Other considered patient characteristics were found not to be significant.

Our work has several important strengths. To our knowledge, this is the largest study assessing family satisfaction with ICU care. Nesting our study within the national clinical audit programme was efficient and novel and allowed for unbiased selection and stratification of participating units and linkage of family members' to patient data. One important strength is that the same mode and timing of delivery of the FS-ICU-24 was employed for family members of ICU survivors and non-survivors, avoiding potential sampling bias and allowing for meaningful comparisons between these groups. Finally, the large sample size of family members allowed for robust multilevel multivariable

For peer review only - http://bmjopen.bmj.com/site/about/guidelines.xhtml

modelling of factors associated with overall family satisfaction to inform important adjustment of any future assessment using this questionnaire. Despite our very large sample size, we achieved a modest response rate (58%), however this was similar to other studies with smaller sample sizes (10, 14).

In conclusion, this large, prospective, multicentre cohort study indicated that overall family satisfaction with adult general ICU care in the UK was high. However, adjustment for differences in family member/patient characteristics are important to avoid falsely identifying ICUs as outliers.

For peer review only

Acknowledgements

We wish to thank the NIHR Health Services and Delivery Research programme for funding this project. We also wish to thank all the patients, family members and staff from all the units that participated the study.

Research staff at sites

C Smalley and R Jacob (Arrowe Park Hospital); S Chau, S A Pearson, K Ellis and R Watmough (Barnsley Hospital); M Faulkner, L Evans and H Robertson (Countess of Chester Hospital); P Wakefield, R Abrahams, N Summers and H Wooldridge (Darent Valley Hospital); H McMillan, S Tyson, K Tantam, S Olver, C Brown and C Tippett (Derriford Hospital); S Moreton, S Jones, A Deeney, J Gibbins and A Oglesby (Dorset County Hospital); C Randell, M Allsop, K Harris, C Scott and C Boyd (Freeman Hospital); E Coughlan, A Jefferies and K Wylie (Manchester Royal Infirmary); C Plowright, C Pegg, L Cooper and T Hatton (Medway Maritime Hospital); P Doble, P Richards, D Bayford and K Adams (Musgrove Park Hospital); J Spimpolo, M Burt and R Pillai (Northampton General Hospital); K A Simeson and S Buckley (Pinderfields Hospital); A Jackson, M Nadolski and H Baker (Royal Devon & Exeter Hospital, Wonford); N Mason, U Gunter and L Roberts (Royal Gwent Hospital); T Evans, E Cooke, M Ogden and P Dark (Salford Royal Hospital); M Cody, F Hogg and D McCahery (South West Acute Hospital); D Dawson, J Mellinghoff, S Prudden, N Poonuth and C Ryan (St George's Hospital); G Mandersloot, A Smith (The Royal London Hospital); S Hagan, L Humphries and E Murphy (Ulster Hospital); E Walker, H Payne and X Zhao (Watford General Hospital); C Edmondson, S Anglesea and H Williams (Wrexham Maelor Hospital).

Study Steering Committee

Dr Kathleen Daly (independent chair); Andrina Colquoun (independent); Dr Maureen Dalziel; Kirsty Everingham (independent); Doreen Henry (independent); Joan Pearson (independent); Catherine Plowright; Dr Laura Price (independent); Professor Kathryn Rowan; Professor Mervyn Singer (independent); and Dr Stephen Wright.

Funding: This project was funded by the National Institute for Health Research (NIHR) Health Services and Delivery Research (HS&DR) Programme (11/2003/56). The funder had no involvement in study design; in collection, analysis and interpretation of data; in the writing of the report; or in the decision to submit the article for publication. The views and opinions expressed therein are those of the authors and do not necessarily reflect those of the HS&DR Programme, NIHR, NHS or the Department of Health.

For peer review only - http://bmjopen.bmj.com/site/about/guidelines.xhtml

Competing interests: Kathryn M Rowan is a member of the NIHR HS&DR Board. Elaine McColl was an editor for the NIHR Journals Library between 2013 and 2016 and received a fee for this work. The other authors declare no conflicts of interest. All authors have completed the Unified Competing Interest form (available on request from the corresponding author).

Data sharing: data can be obtained from the corresponding author on request

Authors contributions: KMR as Chief Investigator conceived the idea and designed the study with DAH, SHE, DKH, LH, EMc, MR, and SEW. EW co-ordinated the study and contributed to data acquisition with ARB, RRC, SHE, and SEW. PVP, DWG, DAH, SHE, DKH, LH, EMc, MR, SEW, and KMR were involved in the analysis and interpretation of the results. All authors were involved in the drafting, editing and have approved the final manuscript.

For peer review only

For peer review only - http://bmjopen.bmj.com/site/about/guidelines.xhtml

References

1. Black N, Jenkinson C. Measuring patients' experiences and outcomes. BMJ. 2009;339:b2495.
2. Heyland DK, Tranmer JE. Measuring family satisfaction with care in the intensive care unit: the development of a questionnaire and preliminary results. J Crit Care. 2001;16(4):142-9.
3. Rothen HU, Stricker KH, Heyland DK. Family satisfaction with critical care: measurements and messages. Curr Opin Crit Care. 2010;16(6):623-31.
4. Wall RJ, Engelberg RA, Downey L, Heyland DK, Curtis JR. Refinement, scoring, and validation of the Family Satisfaction in the Intensive Care Unit (FS-ICU) survey. Crit Care Med. 2007;35(1):271-9.
5. Wright SE, Walmsley E, Harvey SE, Robinson E, Ferrando-Vivas P, Harrison DA, et al. Family-Reported Experiences Evaluation (FREE) study: a mixed-methods study to evaluate families' satisfaction with adult critical care services in the NHS. Health Serv Deliv Res. 2015;3(45).
6. DA H, P F-V, SE W, E M, DK H, KM R. Psychometric assessment of the Family Satisfaction in the Intensive Care Unit questionnaire in the United Kingdom. J Crit Care. 2017;38:346-50.
7. Snijders TAB, Bosker RJ. Multilevel Analysis: An Introduction to Basic and Advanced Multilevel Modeling. Thousand Oaks, CA: SAGE Publications; 1999.
8. Spiegelhalter DJ. Funnel plots for comparing institutional performance. Stat Med. 2005;24(8):1185-202.
9. Carpenter JR, Goldstein H, Kenward MG. REALCOM-IMPUTE software for multilevel multiple imputation with mixed response types. Journal of Statistical Software. 2011;45:5.
10. Dodek PM, Wong H, Heyland DK, Cook DJ, Rocker GM, Kutsogiannis DJ, et al. The relationship between organizational culture and family satisfaction in critical care. Crit Care Med. 2012;40(5):1506-12.
11. Khalaila R. Patients' family satisfaction with needs met at the medical intensive care unit. Journal of Advanced Nursing. 2013(69):1172-82.
12. Schwarzkopf D, Behrend S, Skupin H, Westermann I, Riedemann NC, Pfeifer R, et al. Family satisfaction in the intensive care unit: a quantitative and qualitative analysis. Intensive Care Med. 2013;39(6):1071-9.
13. Tastan S, Iyigun E, Ayhan H, Kilickaya O, Yilmaz AA, Kurt E. Validity and reliability of Turkish version of family satisfaction in the intensive care unit. Int J Nurs Pract. 2014;20(3):320-6.
14. Wall RJ, Curtis JR, Cooke CR, Engelberg RA. Family satisfaction in the ICU: differences between families of survivors and nonsurvivors. Chest. 2007;132(5):1425-33.
15. Stricker KH, Kimberger O, Schmidlin K, Zwahlen M, Mohr U, Rothen HU. Family satisfaction in the intensive care unit: what makes the difference? Intensive Care Med. 2009;35(12):2051-9.

For peer review only

For peer review only - http://bmjopen.bmj.com/site/about/guidelines.xhtml

Figure legends

Figure 1 Distribution of overall family satisfaction score

Figure 2 Variation across ICUs in the mean overall family satisfaction score (A) before and (B) after adjustment for patient and family member characteristics

For peer review only

For peer review only - http://bmjopen.bmj.com/site/about/guidelines.xhtml

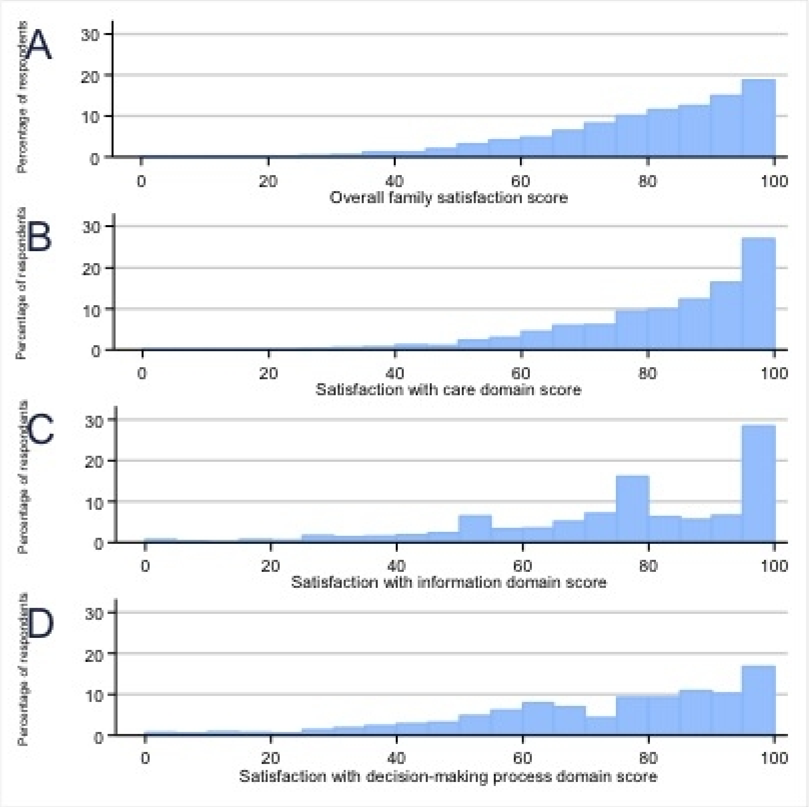

Figure 1 Distribution of overall family satisfaction score

145x145mm (72 x 72 DPI)

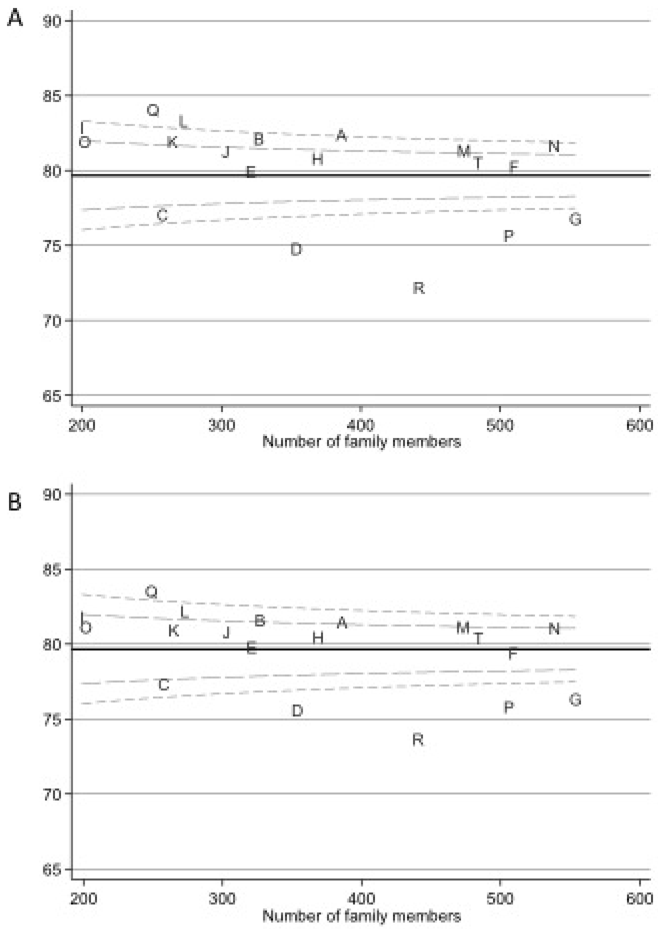

Figure 2 Variation across ICUs in the mean overall family satisfaction score (A) before and (B) after adjustment for patient and family member characteristics

120x172mm (72 x 72 DPI)

Supplementary material

Family satisfaction with critical care in the United Kingdom: a multi-centre cohort study

For peer review only

Table S1 Characteristics and outcomes for all admission to ICUs participating in the FREE study and ICNARC Case Mix Programme

	**CMP**	**FREE study**
Total number of ICUs [N]	[209][a]	[19][a]
Total number of admissions [N]	[149,779]	[18,270]
Age *mean* (SD)	61.5 (18.0)	61.5 (18.0)
Sex *male* (%)	82,444 (55.0)	10,316 (56.5)
Ethnicity *n* (%)		
White	135,767 (90.6)	16,439 (90.0)
Asian	4,815 (3.2)	439 (2.4)
Black	3,250 (2.2)	327 (1.8)
Other	2,434 (1.6)	445 (2.4)
Not stated	3,513 (2.3)	620 (3.4)
Distance (km) from patient home to hospital *median* (IQR) [N]	25.0 (54.2) 8.7 (3.9 19.3) [128,169]	31.7 (64.5) 9.2 (4.2 20.8) [18,090]
APACHE II severe co-morbidities *n* (%)		
0	123,437 (82.4)	14,742 (80.7)
1	20,906 (14.0)	2,648 (14.5)
2	5,053 (3.4)	793 (4.3)
3 or more	383 (0.3)	87 (0.5)
Admission type *n* (%) [N]	[149,765]	[18,270]
Medical	87,940 (58.7)	10,039 (54.9)
Elective surgery	34,284 (22.9)	4,761 (26.1)
Emergency surgery	27,541 (18.4)	3,470 (19.0)
Surgical status of surgical admissions *n* (%) [N]	[61,825]	[8,231]
Planned surgery	28,267 (45.7)	3,985 (48.4)
Unplanned surgery	33,558 (54.3)	4,246 (51.6)
ICNARC Physiology Score *mean* (SD)	16.9 (9.3)	16.5 (9.2)
ICNARC predicted risk of death *median* (IQR) [N]	0.10 (0.03 0.33) [142,654]	0.09 (0.03 0.30) [17,261]
APACHE II Acute Physiology Score *mean* (SD)	11.4 (6.1)	11.3 (5.9)
APACHE II Score *mean* (SD)	15.7 (7.0)	15.6 (6.9)
APACHE II predicted risk of death *median* (IQR) [N]	0.12 (0.04 0.29) [132,197]	0.11 (0.04 0.28) [16,193]
Mechanical ventilation during first 24 hrs *n* (%) [N]	58,687 (39.4) [148,975]	7,008 (38.5) [18,187]

ICU mortality *n* (%) [N]	21,505 (14.4) [149,779]	2,560 (14.0) [18,270]
Acute hospital mortality *n* (%) [N]	29,945 (21.0) [142,670]	3,550 (20.6) [17,266]

[a] excludes one ICU for which no CMP data were available

For peer review only

Figure S1 Overview of patients, family members and questionnaires (distributed/returned)

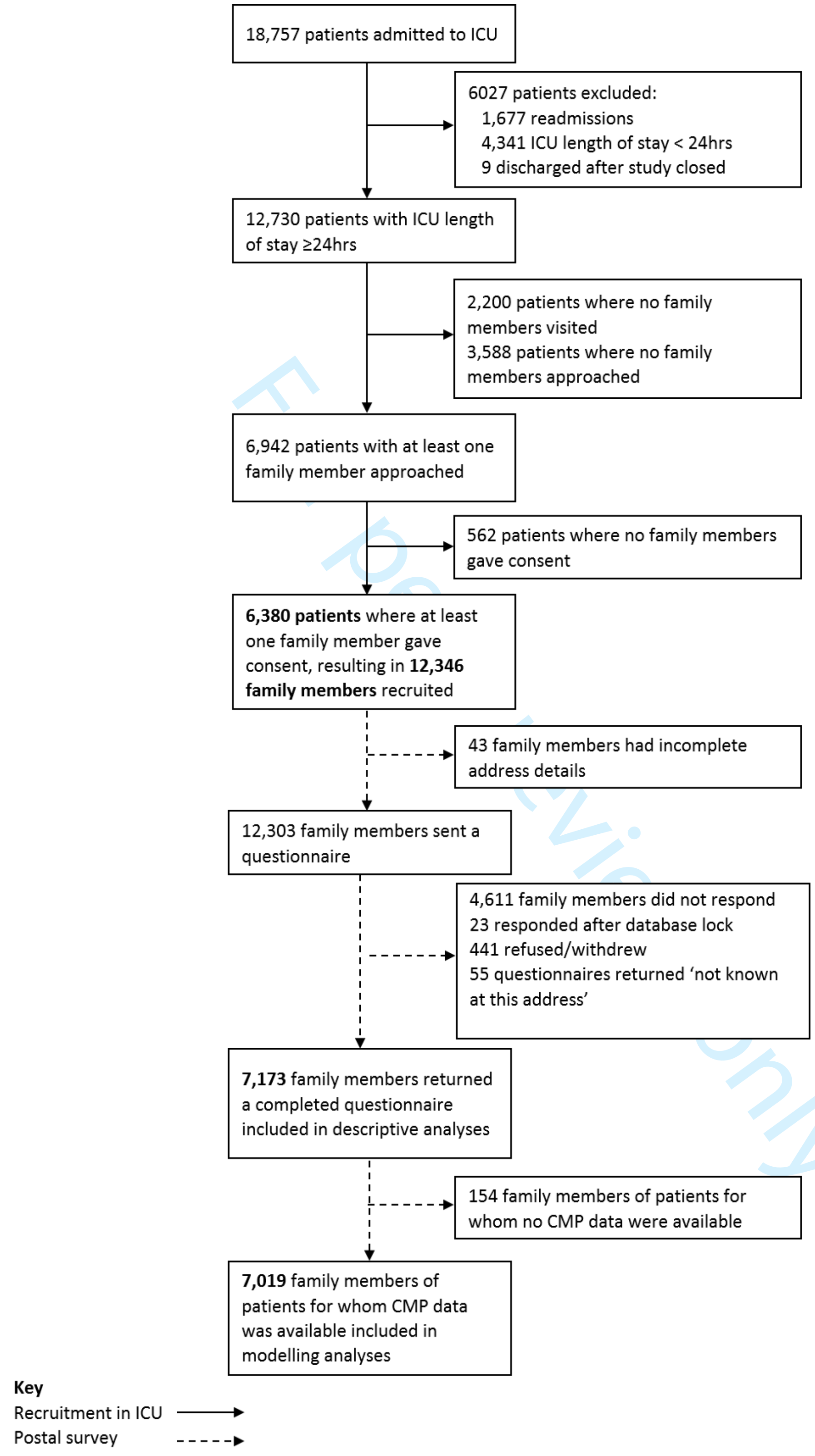

Table S2 Characteristics of all recruited family members and by response to questionnaire

	**All recruited family members**	**Those returning questionnaires**	**Did not respond**
Total number of family members, N	12 346	7173	4611
Age group, *n* (%) [N]	[12 068]	[7019]	[4500]
<30	1429 (11.8)	530 (7.6)	861 (19.1)
30-39	1590 (13.2)	721 (10.3)	827 (18.4)
40-49	2760 (22.9)	1465 (20.9)	1208 (26.9)
50-59	2646 (21.9)	1654 (23.6)	886 (19.7)
60-69	2131 (17.7)	1580 (22.5)	440 (9.8)
70-79	1211 (10.0)	862 (12.3)	220 (4.8)
80+	301 (2.5)	207 (2.9)	58 (1.3)
Sex, *n* (%) [N]	[12 145]	[7062]	[4529]
Female	7687 (63.3)	4689 (66.4)	2663 (58.8)
Male	4458 (36.7)	2373 (33.6)	1866 (41.2)
Ethnicity, *n* (%) [N]	[12 090]	[7033]	[4505]
White	11 379 (94.1)	6747 (95.9)	4111 (91.3)
Asian	355 (2.9)	142 (2.0)	196 (4.4)
Black	161 (1.3)	55 (0.8)	101 (2.2)
Other	195 (1.6)	89 (1.3)	97 (2.1)
Deprivation, *n* (%) [N]	[11 740]	[6832]	[4370]
1 [least deprived]	2113 (18.0)	1376 (20.1)	634 (14.5)
2	2406 (20.5)	1502 (22.0)	803 (18.4)
3	2415 (20.6)	1443 (21.1)	851 (19.5)
4	2545 (21.7)	1380 (20.2)	1045 (23.9)
5 [most deprived]	2261 (19.3)	1131 (16.6)	1037 (23.7)
Distance (km) from family member home to hospital, *median* (IQR) [N]	11.6 (5.1-30.7) [11 803]	12.3 (5.3-33.2) [6867]	10.7 (4.6-29.4) [4394]
Relationship, *n* (%) [N] "I am the patient's…"	[12 343]	[7173]	[4611]
Partner	3105 (25.2)	2151 (30.0)	786 (17.0)
Child	4186 (33.9)	2292 (32.0)	1780 (38.6)
Parent	1054 (8.5)	665 (9.3)	338 (7.3)
Sibling	1271 (10.3)	717 (10.0)	480 (10.4)
Other relative	1973 (16.0)	987 (13.8)	898 (19.5)
Other non-relative	754 (6.1)	361 (5.0)	329 (7.1)
Next-of-kin, *n* (%) [N]	[11 702]	[6770]	[4389]
No	7086 (60.6)	3747 (55.3)	3009 (68.6)
Yes	4616 (39.4)	3023 (44.7)	1380 (31.4)
Lives with patient, *n* (%) [N]	[12 343]	[7172]	[4609]
No	8255 (66.9)	4543 (63.3)	3357 (72.8)
Yes	4088 (33.1)	2629 (36.7)	1252 (27.2)
Education level, *n* (%) [N]	[10 293]	[5971]	[3888]
NVQ 1 or 2	3147 (30.6)	1731 (29.0)	1284 (33.0)
NVQ 3	2086 (20.3)	1149 (19.2)	870 (22.4)

For peer review only

NVQ 4 or 5	2936 (28.5)	1819 (30.5)	1032 (26.5)
Other	2124 (20.6)	1272 (21.3)	702 (18.1)
First language, *n* (%) [N]	[12 346]	[7 173]	[4611]
Not English	335 (2.7)	140 (2.0)	182 (3.9)
English	12 011 (97.3)	7 033 (98.0)	4429 (96.1)

For peer review only

Table S3 Univariable analyses of factors associated with overall family satisfaction score by ICU outcome – family member characteristics

Variables	Family members of ICU survivors [N=6,147[a]]			Family members of ICU non-survivors [N=870]		
	Coef.	95% CI	p-value	Coef.	95% CI	p-value
Age, years (vs < 30)			0.031			0.033
30-39	1.56	(-0.22, 3.33)		2.68	(-1.80, 7.17)	
40-49	0.42	(-0.10, 0.94)		1.61	(0.21, 3.01)	
50-59	2.12	(0.61, 3.64)		5.49	(1.49, 9.50)	
60-69	1.96	(0.39, 3.52)		6.01	(1.78, 10.25)	
70-79	1.98	(0.28, 3.68)		7.39	(2.58, 12.19)	
80+	-0.55	(-3.05, 1.95)		2.62	(-3.48, 8.73)	
Female (vs male)	0.40	(-0.34, 1.14)	0.29	0.44	(-1.59, 2.47)	0.67
White ethnicity (vs non-white)	3.60	(1.46, 5.75)	0.001	8.78	(1.85, 15.70)	0.013
Relationship (vs partner)			<0.001			0.28
Parent	0.00	(-1.39, 1.39)		0.08	(-5.73, 5.90)	
Child	-0.94	(-1.83, -0.05)		-1.274	(-3.69, 1.14)	
Sibling	-2.16	(-3.50, -0.82)		0.909	(-3.02, 4.84)	
Other-relative	-1.63	(-2.81, -0.44)		-0.619	(-3.60, 2.36)	
Other-non relative	-3.42	(-5.22, -1.62)		-6.134	(-11.69, -0.58)	
Next of kin	1.74	(1.05, 2.44)	<0.001	2.69	(0.78, 4.59)	0.006
Lives with patient	1.95	(1.20, 2.69)	<0.001	1.15	(-0.99, 3.29)	0.29
Education (vs NVQ 1 or 2)			<0.001			0.16
NVQ 3	-0.60	(-1.77, 0.57)		1.14	(-2.09, 4.37)	
NVQ 4 or 5	-2.43	(-3.49, -1.37)		-2.07	(-4.92, 0.77)	
Other	-0.18	(-1.35, 0.98)		-1.75	(-4.73, 1.24)	
Quintile of deprivation (vs 1, least deprived)			0.63			0.77
2	0.49	(-0.74, 1.72)		0.64	(-2.73, 4.01)	
3	0.96	(-0.29, 2.20)		0.84	(-2.59, 4.26)	
4	0.32	(-0.97, 1.60)		-1.07	(-4.59, 2.44)	
5 (most deprived)	0.67	(-0.70, 2.05)		0.79	(-3.10, 4.69)	
Distance from home to hospital (per 10 km)	-0.05	(-0.11, 0.01)	0.12	0.05	(-0.09, 0.18)	0.49
Previous experience of ICU as a family member	0.25	(-0.63, 1.14)	0.58	-0.68	(-3.22, 1.87)	0.60
Frequent visitor	2.52	(1.63, 3.41)	<0.001	2.91	(0.36, 5.47)	0.030

Coef., coefficient.

[a] Two family members returned questionnaires but did not complete any of the 24 FS-ICU items – responses were not imputed for these family members.

For peer review only

Table S4 Univariable analyses of factors associated with overall family satisfaction score by ICU outcome – patient characteristics

Variables	Family members of ICU survivors [N=6,147[a]]			Family members of ICU non-survivors [N=870]		
	Coef.	95% CI	p-value	Coef.	95% CI	p-value
Age (per 10 years)	-0.09	(-0.36, 0.17)	0.49	1.12	(0.11, 2.14)	0.030
Female (vs male)	0.67	(-0.25, 1.59)	0.16	2.04	(-0.66, 4.74)	0.14
White ethnicity (vs non-white)	2.39	(0.11, 4.68)	0.040	9.25	(2.38, 16.12)	0.008
Quintile of deprivation (vs 1, least deprived)			0.76			0.95
2	0.86	(-0.66, 2.38)		-1.28	(-5.85, 3.29)	
3	0.62	(-0.90, 2.13)		-0.68	(-5.12, 3.75)	
4	0.77	(-0.75, 2.28)		-1.62	(-6.03, 2.78)	
5 (most deprived)	1.00	(-0.57, 2.57)		-1.49	(-6.04, 3.06)	
Distance from home to hospital (per 10 km)	0.12	(0.00, 0.24)	0.047	0.18	(-0.05, 0.41)	0.12
Severe comorbidities						
Liver	3.18	(-0.01, 6.38)	0.050	1.25	(-4.67, 7.19)	0.68
Renal	-0.45	(-3.57, 2.66)	0.77	-8.87	(-18.35, 0.60)	0.067
Respiratory	0.01	(-2.84, 2.85)	1.00	-1.02	(-7.23, 5.19)	0.75
Cardiovascular	-0.14	(-3.23, 2.94)	0.93	1.40	(-6.46, 9.26)	0.73
Metastatic cancer	-2.81	(-5.78, 0.15)	0.063	3.26	(-6.38, 12.90)	0.51
Haematological malignancy	2.25	(-1.09, 5.61)	0.19	-7.88	(-14.62, -1.13)	0.022
Immunocompromise	-0.91	(-2.74, 0.90)	0.33	-3.90	(-8.55, 0.74)	0.10
Dependency (vs none)			0.30			0.85
Minor or major	-0.14	(-1.36, 1.08)		0.63	(-2.34, 3.60)	
Total	-3.63	(-8.21, 0.94)		2.73	(-10.21, 15.67)	
Surgical status (vs non-surgical)			0.005			0.78
Planned elective/scheduled	-2.17	(-3.51, -0.83)		-2.83	(-10.75, 5.10)	
Unplanned	-0.17	(-1.29, 0.96)		-0.06	(-3.89, 3.76)	
ICNARC Physiology Score (per point)	0.19	(0.13, 0.25)	<0.001	0.19	(0.02, 0.35)	0.026
ICU length of stay (per day)	0.02	(-0.03, 0.06)	0.44	-0.34	(-0.48, -0.20)	<0.001
Advanced respiratory support	3.62	(2.63, 4.61)	<0.001	1.96	(-1.84, 5.76)	0.31
Advanced cardiovascular support	2.06	(0.89, 3.22)	0.001	0.83	(-2.06, 3.72)	0.58
Renal support	1.52	(0.11, 2.93)	0.034	0.04	(-2.83, 2.91)	0.98
Neurological support	1.96	(0.39, 3.54)	0.014	2.95	(-0.42, 6.32)	0.086
Duration of adv. respiratory support (per day)	0.11	(0.05, 0.16)	<0.001	-0.16	(-0.32, 0.00)	0.051
Duration of adv. cardiovascular support (per day)	0.40	(0.15, 0.65)	0.002	0.11	(-0.33, 0.56)	0.62
Duration of renal support (per day)	0.16	(0.00, 0.32)	0.048	-0.15	(-0.43, 0.13)	0.28
Duration of neurological support (per day)	0.10	(-0.09, 0.29)	0.31	0.05	(-0.43, 0.53)	0.84
Death before acute hospital discharge	-0.49	(-1.52, 0.55)	0.36	N/A		

Coef., coefficient.

For peer review only

[a] Two family members returned questionnaires but did not complete any of the 24 FS-ICU items – responses were not imputed for these family members.

For peer review only

Table S5 Univariable analysis of factors associated with overall family satisfaction score by ICU outcome – ICU/hospital characteristics and contextual factors

**Variables**	**Family members of ICU survivors [N=6,147[a]]**			**Family members of ICU non-survivors [N=870]**		
	Coef.	95% CI	p-value	Coef.	95% CI	p-value
Hospital type (vs non-university)			0.51			0.62
University	0.06	(-3.63, 3.75)		-0.32	(-4.72, 4.07)	
University affiliated	1.93	(-1.56, 5.42)		1.68	(-2.29, 5.65)	
Number of ICU beds (per bed)	-0.05	(-0.23, 0.14)	0.63	0.02	(-0.22, 0.26)	0.85
Month of ICU admission (vs January)			0.95			0.85
February	-0.61	(-2.87, 1.65)		-0.03	(-6.90, 6.83)	
March	0.09	(-2.12, 2.30)		-0.06	(-6.73, 6.60)	
April	0.54	(-1.71, 2.79)		0.07	(-6.93, 7.07)	
May	-0.06	(-2.31, 2.18)		0.73	(-5.62, 7.08)	
June	-0.66	(-2.65, 1.34)		0.84	(-4.95, 6.64)	
July	0.85	(-1.41, 3.11)		3.91	(-2.71, 10.52)	
August	0.65	(-1.64, 2.93)		-0.70	(-6.87, 5.46)	
September	0.09	(-2.14, 2.31)		1.74	(-4.76, 8.25)	
October	0.44	(-1.76, 2.63)		1.15	(-5.69, 7.98)	
November	0.60	(-1.65, 2.85)		2.21	(-4.10, 8.53)	
December	0.69	(-1.57, 2.96)		5.16	(-1.13, 11.46)	
Questionnaire received while patient still in hospital	0.087	(-1.50, 1.67)	0.91	N/A		

Coef., coefficient.

[a] Two family members returned questionnaires but did not complete any of the 24 FS-ICU items – responses were not imputed for these family members.

For peer review only

Table S6 Sensitivity analyses –candidate determinants for the multivariable multilevel models for the family satisfaction in the intensive care unit

Candidate determinants	Justification inclusion/exclusion	Approach to modelling
**Family member level**		
Education level	It was not considered in the multivariable models due to higher than expected proportions of both "Not stated" (17%) and "Other" (21%) responses, suggesting a lack of comprehension of the categorisation used.	
Distance from home to hospital	No significant after adjusting for other variables in the model. It was dropped.	
Family member age, years	Controlling effect	Categorical (<30;30-39;40-49;50-59;60-69;70-79;80+)
Family member sex	Controlling effect	Categorical (male; female)
Family member ethnicity	Statistically significant in univariable	Categorical (white; non-white)
Next-of-kin/lives with patient	There was a strong multicollinearity between relationship to the patient and the other key variables of next-of-kin and lives with patient.	Categorical (lives with patient; Next-of-kin, does not live with patient; Not next-of-kin, does not live with patient)
Frequent visitor	Statistically significant in univariable	Binary (yes; no)
**Patient level**		
Patient ethnicity	It was not carried forward to the multivariable models due to collinearity with family member ethnicity.	
Patient age	Controlling effect	Continuous(linear)
Patient sex	Controlling effect	Categorical (male; female)
Dependency	Controlling effect	Categorical (none; minor or major; total)
Surgical status (vs non-surgical)	Controlling effect	Categorical (non-surgical; planned elective/scheduled; unplanned)
ICNARC Physiology Score	Statistically significant in univariable	Continuous(linear)
ICU length of stay (days)		Continuous(linear)
Organ support received in the ICU and duration (calendar days)	Once included in the multivariable model for	

For peer review only

of organ support among those receiving the support	survivors, only advanced respiratory support remained significant.	
Advanced respiratory support	It was found to be preferable to alternative variable of the duration of advanced respiratory support, which was correlated with ICU length of stay.	Binary (yes; no)
haematological malignancy	No significant after adjusting for other variables in the model. It was dropped.	
**ICU/hospital level**		
Hospital type	Controlling effect	Categorical (non-university; university; university affiliated)
Number of ICU beds	Controlling effect	Continuous(linear)

For peer review only

Table S7 Multivariable multilevel models for the satisfaction with care domain score

Variables	Family members of ICU survivors [N=6,143[a]]			Family members of ICU non-survivors [N=869[a]]		
	Coef.	95% CI	p-value	Coef.	95% CI	p-value
Fixed effects – family member level						
Constant	71.45	(66.67, 76.22)	<0.001	55.29	(41.76, 68.82)	<0.001
Family member age, years (vs <30)			0.001			0.16
30-39	2.60	(0.81, 4.38)		2.50	(-1.97, 6.97)	
40-49	2.73	(1.16, 4.31)		4.31	(0.09, 8.54)	
50-59	2.91	(1.36, 4.44)		4.99	(0.93, 9.04)	
60-69	2.67	(1.08, 4.26)		4.89	(0.54, 9.23)	
70-79	2.66	(0.90, 4.41)		5.91	(0.88, 10.94)	
80+	-0.17	(-2.76, 2.41)		1.85	(-4.51, 8.21)	
Family member sex – female (vs male)	0.42	(-0.35, 1.20)	0.29	0.22	(-1.81, 2.25)	0.83
Family member ethnicity – white (vs non-white)	3.87	(1.77, 5.97)	<0.001	6.99	(0.19, 13.81)	0.044
Next-of-kin/lives with patient (vs lives with patient)			<0.001			0.15
Next-of-kin, does not live with patient	-1.14	(-2.26, -0.02)		0.95	(-2.39, 4.29)	
Not next-of-kin, does not live with patient	-2.44	(-3.32, -1.55)		-1.58	(-4.11, 0.94)	
Frequent visitor	2.49	(1.52, 3.46)	<0.001	1.49	(-1.27, 4.25)	0.29
Fixed effects – patient level						
Patient age (per 10 years)	0.03	(-0.25, 0.31)	0.83	1.21	(0.16, 2.26)	0.024
Patient sex – female (vs male)	0.06	(-0.85, 0.98)	0.87	1.85	(-0.79, 4.5)	0.17
Dependency (vs none)			0.006			0.68
Minor or major	-0.74	(-1.96, 0.46)		-0.94	(-3.98, 2.09)	
Total	-6.77	(-11.18, -2.36)		3.62	(-8.71, 15.95)	
Surgical status (vs non-surgical)			0.68			0.47
Planned elective/scheduled	-0.62	(-2.04, 0.78)		-4.85	(-12.71, 2.99)	
Unplanned	-0.15	(-1.27, 0.95)		-0.57	(-4.29, 3.13)	
ICNARC Physiology Score (per point)	0.14	(0.07, 0.21)	<0.001	0.14	(-0.03, 0.30)	0.10
ICU length of stay (per day)	-0.02	(-0.06, 0.02)	0.39	-0.30	(-0.45, -0.15)	<0.001
Advanced respiratory support	2.74	(1.66, 3.82)	<0.001			
Fixed effects – ICU/hospital level						
Hospital type (vs non-university)			0.51			0.58

For peer review only

University	0.94	(-3.58, 5.47)		-1.48	(-7.8 , 4.84)	
University affiliated	1.92	(-1.34, 5.19)		1.79	(-2.75, 6.34)	
Number of ICU beds (per bed)	-0.01	(-0.24, 0.23)	0.96	0.24	(-0.12, 0.59)	0.19
Random effects – SD (SE)						
Between ICUs	2.98	(0.60)		3.25	(1.11)	
Within ICUs between patients	9.76	(0.28)		10.47	(0.66)	
Within patients between family members	11.96	(0.19)		11.92	(0.42)	

Coef, coefficient; SE, standard error.

[a] Five patients were missing age group on both the questionnaire and web portal – due to the very small amount of missing data in this key variable, these missing values were not imputed.

For peer review only

Table S8 Multivariable multilevel models for the satisfaction with information domain score

**Variables**	**Family members of ICU survivors [N=6,143[a]]**			**Family members of ICU non-survivors [N=869[a]]**		
	Coef.	95% CI	p-value	Coef.	95% CI	p-value
Fixed effects – family member level						
Constant	66.07	(59.78, 72.21)	<0.001	55.86	(39.34, 72.38)	<0.001
Family member age, years (vs <30)			0.63			0.28
30-39	0.28	(-2.22, 2.79)		1.23	(-4.92, 7.39)	
40-49	0.00	(-2.21, 2.21)		1.88	(-3.92, 7.68)	
50-59	0.55	(-1.62, 2.72)		2.88	(-2.70, 8.48)	
60-69	-0.1	(-2.35, 2.14)		4.24	(-1.71, 10.2)	
70-79	-0.41	(-2.89, 2.08)		6.43	(-0.45, 13.31)	
80+	-2.67	(-6.35, 1.01)		-1.96	(-10.71, 6.79)	
Family member sex – female (vs male)	0.20	(-0.89, 1.30)	0.72	1.01	(-1.81, 3.82)	0.49
Family member ethnicity – white (vs non-white)	4.73	(1.78, 7.68)	0.002	9.34	(0.47, 18.21)	0.039
Next-of-kin/lives with patient (vs lives with patient)			<0.001			0.38
Next-of-kin, does not live with patient	-2.39	(-3.97, 0.81)		1.43	(-3.09, 5.95)	
Not next-of-kin, does not live with patient	-2.57	(-3.83, 1.31)		-1.21	(-4.69, 2.28)	
Frequent visitor	2.11	(0.74, 3.48)	0.002	0.44	(-3.33, 4.22)	0.82
Fixed effects – patient level						
Patient age (per 10 years)	-0.22	(-0.61, 0.18)	0.28	0.92	(-0.43, 2.27)	0.18
Patient sex – female (vs male)	0.32	(-0.98, 1.62)	0.63	1.93	(-1.48, 5.35)	0.27
Dependency (vs none)			0.61			0.51
Minor or major	-0.49	(-2.2, 1.2)		-0.28	(-4.11, 3.53)	
Total	-2.69	(-8.92, 3.52)		9.15	(-6.57, 24.87)	
Surgical status (vs non-surgical)			0.88			0.84
Planned elective/scheduled	-0.32	(-2.32, 1.66)		-0.88	(-10.97, 9.21)	
Unplanned	0.23	(-1.33, 1.80)		-1.4	(-6.16, 3.36)	
ICNARC Physiology Score (per point)	0.23	(0.13, 0.33)	<0.001	0.15	(-0.04, 0.36)	0.13
ICU length of stay (per day)	-0.05	(-0.11, 0.01)	0.14	-0.43	(-0.62, -0.24)	<0.001
Advanced respiratory support	3.34	(1.83, 4.85)	<0.001	--		
Fixed effects – ICU/hospital level						
Hospital type (vs non-university)			0.45			0.58

For peer review only

University	1.69	(-3.71, 7.08)		0.35	(-6.42, 7.13)	
University affiliated	2.48	(-1.42, 6.40)		2.53	(-2.32, 7.39)	
Number of ICU beds (per bed)	-0.03	(-0.31, 0.24)	0.81	0.21	(-0.17, 0.61)	0.27
Random effects – SD (SE)						
Between ICUs	3.48	(0.73)		2.81	(1.37)	
Within ICUs between patients	13.64	(0.41)		12.38	(0.97)	
Within patients between family members	16.88	(0.27)		17.02	(0.60)	

Coef, coefficient; SE, standard error.

[a] Five patients were missing age group on both the questionnaire and web portal – due to the very small amount of missing data in this key variable, these missing values were not imputed.

For peer review only

Table S9 Multivariable multilevel models for the satisfaction with the decision-making process domain score

Variables	Family members of ICU survivors [N=6,143[a]]			Family members of ICU non-survivors [N=869[a]]		
	Coef.	95% CI	p-value	Coef.	95% CI	p-value
Fixed effects – family member level						
Constant	61.65	(55.17, 68.14)	<0.001	39.62	(20.14, 59.09)	<0.001
Family member age, years (vs <30)			0.061			0.40
30-39	1.66	(-1.63, 4.95)		1.37	(-5.35, 8.10)	
40-49	0.02	(-2.76, 2.82)		2.73	(-3.47, 8.95)	
50-59	0.52	(-2.21, 3.25)		3.34	(-2.61, 9.31)	
60-69	-1.43	(-4.48, 1.61)		3.35	(-3.05, 9.77)	
70-79	-1.09	(-4.32, 2.13)		6.25	(-1.36, 13.88)	
80+	-3.87	(-8.43, 0.69)		-3.13	(-12.88, 6.61)	
Family member sex – female (vs male)	-0.18	(-1.42, 1.04)	0.77	1.66	(-1.37, 4.71)	0.28
Family member ethnicity – white (vs non-white)	0.81	(-2.67, 4.30)	0.65	6.46	(-4.24, 17.15)	0.24
Next-of-kin/lives with patient (vs lives with patient)			0.10			0.86
Next-of-kin, does not live with patient	-0.93	(-2.93, 1.05)		1.39	(-3.49, 6.28)	
Not next-of-kin, does not live with patient	-1.65	(-3.22, 0.07)		0.48	(-3.49 , 4.46)	
Frequent visitor	5.31	(3.38, 7.23)	<0.001	3.84	(-0.21, 7.91)	0.063
Fixed effects – patient level						
Patient age (per 10 years)	0.26	(-0.20, 0.73)	0.27	2.19	(0.61, 3.78)	0.007
Patient sex – female (vs male)	0.79	(-0.84, 2.43)	0.34	1.29	(-2.67, 5.26)	0.52
Dependency (vs none)			0.44			0.47
Minor or major	1.34	(-0.74, 3.43)		2.91	(-1.48, 7.29)	
Total	0.11	(-7.42, 7.64)		4.27	(-17.36, 25.91)	
Surgical status (vs non-surgical)			0.25			0.68
Planned elective/scheduled	-1.83	(-4.35, 0.68)		-1.09	(-12.59, 10.41)	
Unplanned	-1.35	(-3.41, 0.71)		2.35	(-3.20, 7.91)	
ICNARC Physiology Score (per point)	0.12	(0.01, 0.24)	0.040	0.19	(-0.04, 0.44)	0.12
ICU length of stay (per day)	0.03	(-0.04, 0.11)	0.39	-0.17	(-0.39, 0.03)	0.11
Advanced respiratory support	3.03	(1.08, 4.97)	0.002	--		
Fixed effects – ICU/hospital level						

For peer review only

Hospital type (vs non-university)			0.50			0.55
University	-0.41	(-4.27, 3.46)		-4.44	(-12.41, 3.53)	
University affiliated	1.51	(-1.37, 4.39)		-0.86	(-6.56, 4.83)	
Number of ICU beds (per bed)	0.02	(-0.19, 0.23)	0.85	0.47	(0.02, 0.93)	0.042
Random effects – SD (SE)						
Between ICUs	2.06	(0.66)		3.33	(1.50)	
Within ICUs between patients	17.24	(0.50)		15.84	(1.06)	
Within patients between family members	17.02	(0.40)		16.81	(0.66)	

Coef, coefficient; SE, standard error.

[a] Five patients were missing age group on both the questionnaire and web portal – due to the very small amount of missing data in this key variable, these missing values were not imputed.

For peer review only

Figure S2 Variation across ICUs in the mean: satisfaction with care domain score (A) before and (B) after adjustment; satisfaction with information domain score (C) before and (D) after adjustment; and satisfaction with the decision-making process domain score (E) before and (F) after adjustment

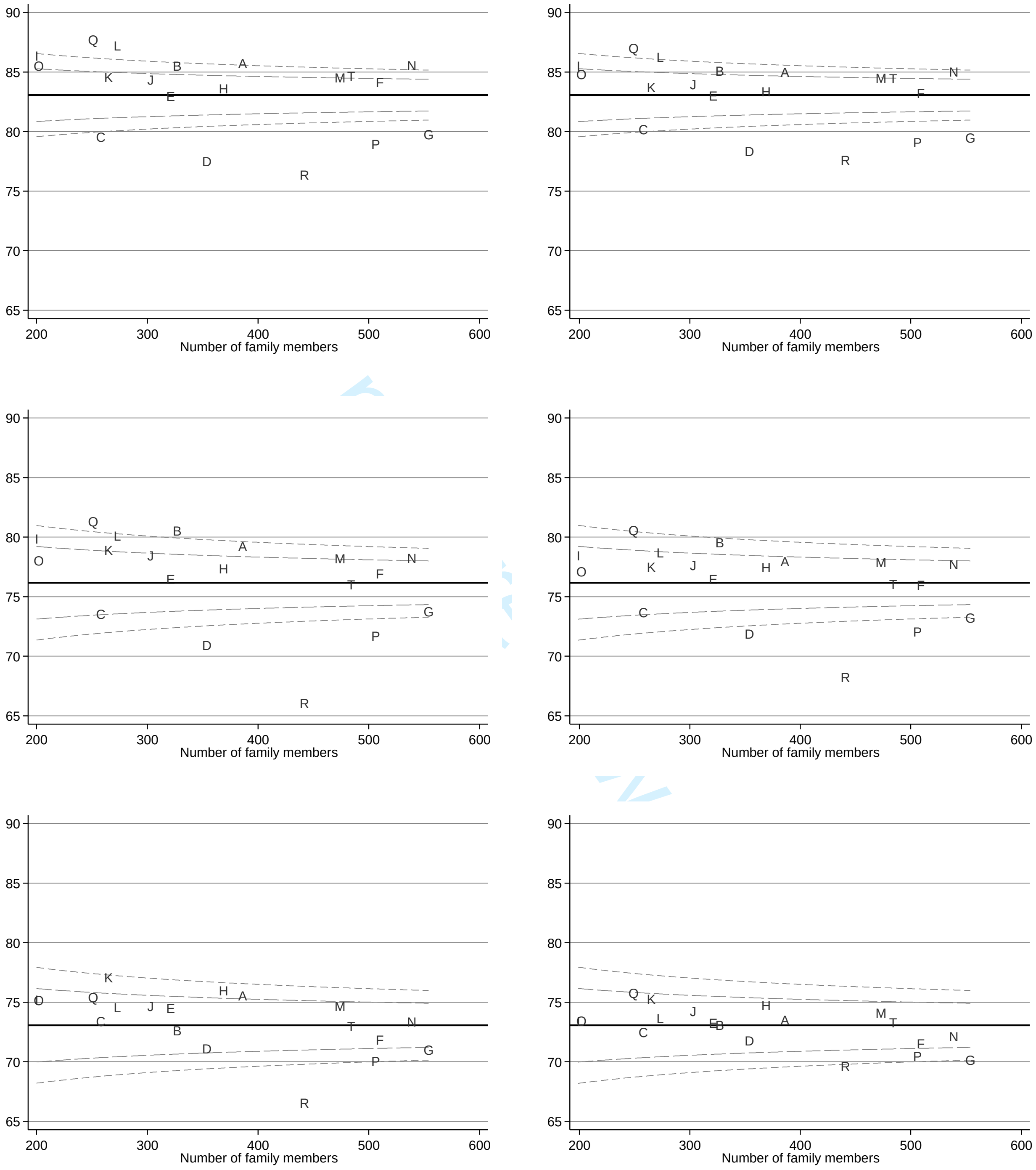

Table S10 Sensitivity analyses – alternative approach to handling missing data (family members of ICU survivors)

Variables	Complete case [N=2,351]			Traditional approach [N=5,756]		
	Coef.	SE	p-value	Coef.	SE	p-value
Constant	72.60	3.18	<0.001	70.35	2.49	<0.001
Family member age, years (vs <30)			0.61			0.20
30-39	0.13	1.40		1.47	0.97	
40-49	0.85	1.22		1.41	0.86	
50-59	0.66	1.20		1.58	0.84	
60-69	0.65	1.30		1.47	0.88	
70-79	0.77	1.47		1.69	0.98	
80+	-3.06	2.26		-1.22	1.50	
Family member sex – female (vs male)	0.94	0.60	0.12	0.21	0.43	0.63
Family member ethnicity – white (vs non-white)	7.58	1.58	<0.001	3.99	1.16	0.001
Next-of-kin/lives with patient (vs lives with patient)			0.071			0.002
Next-of-kin, does not live with patient	-1.69	0.85		-1.36	0.61	
Not next-of-kin, does not live with patient	-1.42	0.72		-1.70	0.50	
Frequent visitor	1.18	0.82	0.15	2.21	0.55	<0.001
Patient age (per 10 years)	-0.09	0.22	0.67	-0.07	0.15	0.64
Patient sex – female (vs male)	-1.20	0.73	0.10	0.13	0.52	0.79
Dependency (vs none)			0.70			0.45
Minor or major	-0.44	0.92		-0.19	0.68	
Total	-2.19	2.98		-3.14	2.51	
Surgical status (vs non-surgical)			0.056			0.47
Planned elective/scheduled	-3.11	1.30		-0.93	0.80	
Unplanned	-0.44	0.88		0.02	0.62	
ICNARC Physiology Score (per point)	0.08	0.05	0.14	0.15	0.04	<0.001
ICU length of stay (per day)	-0.04	0.03	0.28	-0.04	0.03	0.17
Advanced respiratory support	1.39	0.87	0.11	2.40	0.60	<0.001
Hospital type (vs non-university)			0.42			0.34
University	0.56	2.36		1.45	2.22	

For peer review only

University affiliated	2.24	1.72		2.34	1.61	
Number of ICU beds (per bed)	0.07	0.12	0.59	-0.02	0.11	0.83

Coef., coefficient; SE, standard error.

For peer review only

Table S11 Sensitivity analyses – alternative approaches to handling missing data (family members of ICU non-survivors)

**Variables**	**Complete case [N=547]**			**Traditional approach [N=851]**		
	Coef.	SE	p-value	Coef.	SE	p-value
Constant	54.46	7.72	<0.001	56.28	6.80	<0.001
Family member age, years (vs <30)			0.17			0.086
30-39	4.38	3.01		3.14	2.44	
40-49	7.51	2.75		4.87	2.31	
50-59	6.19	2.62		4.50	2.22	
60-69	7.41	2.85		5.94	2.37	
70-79	6.99	3.69		7.07	2.82	
80+	7.52	4.41		0.32	3.61	
Family member sex – female (vs male)	-0.02	1.43	0.99	0.40	1.11	0.72
Family member ethnicity – white (vs non-white)	9.64	4.21	0.022	7.47	3.58	0.037
Next-of-kin/lives with patient (vs lives with patient)			0.97			0.38
Next-of-kin, does not live with patient	0.13	2.20		1.27	1.82	
Not next-of-kin, does not live with patient	-0.32	1.81		-0.82	1.40	
Frequent visitor	1.32	1.96	0.50	0.99	1.51	0.51
Patient age (per 10 years)	0.69	0.66	0.29	1.09	0.55	0.048
Patient sex – female (vs male)	1.56	1.69	0.36	2.02	1.41	0.15
Dependency (vs none)			0.47			0.66
Minor or major	-0.61	1.86		-0.32	1.58	
Total	8.53	7.42		5.59	6.45	
Surgical status (vs non-surgical)			0.84			0.51
Planned elective/scheduled	-0.33	5.61		-4.86	4.22	
Unplanned	-1.38	2.33		-0.44	1.95	
ICNARC Physiology Score (per point)	0.24	0.10	0.022	0.18	0.09	0.041
ICU length of stay (per day)	-0.27	0.09	0.003	-0.33	0.08	<0.001
Hospital type (vs non-university)			0.83			0.77
University	-1.15	3.20		-0.11	3.01	
University affiliated	0.84	2.29		1.49	2.17	

For peer review only

Number of ICU beds (per bed)	0.25	0.19	0.17	0.21	0.17	0.23

Coef., coefficient; SE, standard error.

For peer review only

STROBE Statement—checklist of items that should be included in reports of observational studies

	Item No	Recommendation	Page No
**Title and abstract**	1	(*a*) Indicate the study's design with a commonly used term in the title or the abstract	1
		(*b*) Provide in the abstract an informative and balanced summary of what was done and what was found	3
**Introduction**			
Background/rationale	2	Explain the scientific background and rationale for the investigation being reported	5
Objectives	3	State specific objectives, including any prespecified hypotheses	5
**Methods**			
Study design	4	Present key elements of study design early in the paper	5
Setting	5	Describe the setting, locations, and relevant dates, including periods of recruitment, exposure, follow-up, and data collection	5
Participants	6	(*a*) *Cohort study*—Give the eligibility criteria, and the sources and methods of selection of participants. Describe methods of follow-up<br>*Case-control study*—Give the eligibility criteria, and the sources and methods of case ascertainment and control selection. Give the rationale for the choice of cases and controls<br>*Cross-sectional study*—Give the eligibility criteria, and the sources and methods of selection of participants	6
		(*b*) *Cohort study*—For matched studies, give matching criteria and number of exposed and unexposed<br>*Case-control study*—For matched studies, give matching criteria and the number of controls per case	N/A
Variables	7	Clearly define all outcomes, exposures, predictors, potential confounders, and effect modifiers. Give diagnostic criteria, if applicable	6-7
Data sources/ measurement	8*	For each variable of interest, give sources of data and details of methods of assessment (measurement). Describe comparability of assessment methods if there is more than one group	
Bias	9	Describe any efforts to address potential sources of bias	5
Study size	10	Explain how the study size was arrived at	N/A
Quantitative variables	11	Explain how quantitative variables were handled in the analyses. If applicable, describe which groupings were chosen and why	6-7
Statistical methods	12	(*a*) Describe all statistical methods, including those used to control for confounding	6-7
		(*b*) Describe any methods used to examine subgroups and interactions	N/A
		(*c*) Explain how missing data were addressed	6-7
		(*d*) *Cohort study*—If applicable, explain how loss to follow-up was addressed<br>*Case-control study*—If applicable, explain how matching of cases and controls was addressed<br>*Cross-sectional study*—If applicable, describe analytical methods taking account of sampling strategy	
		(*e*) Describe any sensitivity analyses	7

Continued on next page

For peer review only - http://bmjopen.bmj.com/site/about/guidelines.xhtml

**Results**			
Participants	13*	(a) Report numbers of individuals at each stage of study—eg numbers potentially eligible, examined for eligibility, confirmed eligible, included in the study, completing follow-up, and analysed	7
		(b) Give reasons for non-participation at each stage	
		(c) Consider use of a flow diagram	Supplementary materials Figure S1
Descriptive data	14*	(a) Give characteristics of study participants (eg demographic, clinical, social) and information on exposures and potential confounders	Page 7-8 & Tables 1 & 2
		(b) Indicate number of participants with missing data for each variable of interest	Supplementary materials Tables S10 & S11
		(c) *Cohort study*—Summarise follow-up time (eg, average and total amount)	N/A
Outcome data	15*	*Cohort study*—Report numbers of outcome events or summary measures over time	8 & Table 3
		Case-control study—Report numbers in each exposure category, or summary measures of exposure	
		Cross-sectional study—Report numbers of outcome events or summary measures	
Main results	16	(*a*) Give unadjusted estimates and, if applicable, confounder-adjusted estimates and their precision (eg, 95% confidence interval). Make clear which confounders were adjusted for and why they were included	8 & 9, Table 4 & Supplement Tables S7-9 &
		(*b*) Report category boundaries when continuous variables were categorized	
		(*c*) If relevant, consider translating estimates of relative risk into absolute risk for a meaningful time period	
Other analyses	17	Report other analyses done—eg analyses of subgroups and interactions, and sensitivity analyses	9 & supplement
**Discussion**			
Key results	18	Summarise key results with reference to study objectives	9-10
Limitations	19	Discuss limitations of the study, taking into account sources of potential bias or imprecision. Discuss both direction and magnitude of any potential bias	9-10
Interpretation	20	Give a cautious overall interpretation of results considering objectives, limitations, multiplicity of analyses, results from similar studies, and other relevant evidence	9-10
Generalisability	21	Discuss the generalisability (external validity) of the study results	9-10
**Other information**			
Funding	22	Give the source of funding and the role of the funders for the present study and, if applicable, for the original study on which the present article is based	2 & 11

*Give information separately for cases and controls in case-control studies and, if applicable, for exposed and unexposed groups in cohort and cross-sectional studies.

Note: An Explanation and Elaboration article discusses each checklist item and gives methodological background and published examples of transparent reporting. The STROBE checklist is best used in conjunction with this article (freely

For peer review only - http://bmjopen.bmj.com/site/about/guidelines.xhtml

available on the Web sites of PLoS Medicine at http://www.plosmedicine.org/, Annals of Internal Medicine at http://www.annals.org/, and Epidemiology at http://www.epidem.com/). Information on the STROBE Initiative is available at www.strobe-statement.org.

For peer review only

BMJ Open

Family satisfaction with critical care in the United Kingdom: a multi-centre cohort study

Journal:	*BMJ Open*
Manuscript ID	bmjopen-2019-028956.R1
Article Type:	Research
Date Submitted by the Author:	28-May-2019
Complete List of Authors:	Ferrando, Paloma; Intensive Care National Audit and Research Centre,<br>Gould, Doug; Intensive Care National Audit and Research Centre<br>Walmsley, Emma; Intensive Care National Audit and Research Centre<br>Richards-Belle, Alvin; Intensive Care National Audit and Research Centre<br>Canter, Ruth; Intensive Care National Audit and Research Centre<br>Saunders, Steven; Intensive Care National Audit and Research Centre<br>Harrison, David; Intensive Care National Audit and Research Centre<br>Harvey, Sheila; London School of Hygiene & Tropical Medicine, Global Health and Development<br>Heyland, Daren; Kingston General Hospital, Clinical Evaluation Research Unit; Queens University, Department of Medicine<br>Hinton, Lisa; University of Oxford, Health Experiences Research Group, Nuffield Department of Primary Care Health Sciencs<br>McColl, Elaine; Newcastle Clinical Trials Unit<br>Richardson, Annette; Freeman Hospital, Perioperative and Critical Care<br>Richardson, Michael<br>Wright, Stephen; Freeman Hospital, Perioperative and Critical Care<br>Rowan, Kathryn; Intensive Care National Audit and Research Centre
**Primary Subject Heading**:	Intensive care
Secondary Subject Heading:	Patient-centred medicine
Keywords:	Adult intensive & critical care < ANAESTHETICS, Quality in health care < HEALTH SERVICES ADMINISTRATION & MANAGEMENT, Family satisfaction

SCHOLARONE™
Manuscripts

Family satisfaction with critical care in the United Kingdom: a multi-centre cohort study

Authors

Paloma Ferrando-Vivas, *statistician*[1], Doug W Gould, *senior researcher*[1], Emma Walmsley, *study co-ordinator*[1], Alvin Richards-Belle, *trial manager*[1], Ruth R Canter, *research assistant*[1], Steven Saunders, *research administrator*[1], David A Harrison, *head statistician*[1], Sheila E Harvey, *Associate Professor*[2], Daren K Heyland, *director of the Kingston General Hospital Clinical Evaluation Research Unit and professor of medicine and epidemiology*[3], Lisa Hinton, *senior qualitative researcher*[4], Elaine McColl, *director of Newcastle CTU and professor of health services research*[5], Annette Richardson, *nurse consultant in critical care*[6], Michael Richardson, *patient public representative*[7], Stephen E Wright, *consultant in anaesthesia and intensive care*[6], Kathryn M Rowan, *director of scientific & strategic development*[1].

[1]Intensive Care National Audit & Research Centre (ICNARC), London, United Kingdom, WC1V 6AZ

[2]London School of Hygiene & Tropical Medicine, Global Health and Development, London, United Kingdom

[3]Kingston General Hospital, Clinical Evaluation Research Unit, Kingston, Canada and Queen's University, Department of Medicine, School of Medicine, Canada.

[4] University of Oxford, Health Experiences Research Group, Nuffield Department of Primary Care Health Sciences, Oxford, United Kingdom.

[5]Newcastle Clinical Trials Unit, Newcastle University, Newcastle upon Tyne, United Kingdom.

[6]Freeman Hospital, Perioperative and Critical Care, Newcastle Upon Tyne Hospitals NHS Foundation Trust, Newcastle upon Tyne, United Kingdom.

[7]Newcastle Upon Tyne, United Kingdom.

Corresponding author:

Kathryn M. Rowan (ORCid iD: 0000 0001 8217 5602), Director of Scientific & Strategic Development, Intensive Care National Audit & Research Centre, Napier House, 24 High Holborn, London WC1V 6AZ

Tel: 020 7831 6878

Fax: 020 7831 6879

e-mail: Kathy.rowan@icnarc.org

For peer review only

For peer review only - http://bmjopen.bmj.com/site/about/guidelines.xhtml

Abstract

Objective: To assess family satisfaction with intensive care units (ICUs) in the United Kingdom using the Family Satisfaction in the Intensive Care Unit 24-item questionnaire (FS-ICU-24), and to investigate how characteristics of patients and their family members impact on family satisfaction.

Design: Prospective cohort study nested within a national clinic audit database.

Setting: Stratified, random sample of 20 adult general ICUs participating in the Intensive Care Audit & Research Centre (ICNARC) Case Mix Programme.

Participants: Family members of patients staying at least 24 hours in ICU were recruited between May 2013 and June 2014.

Interventions: Consenting family members were sent a postal questionnaire three weeks after the patient died or was discharged from ICU. Up to four family members were recruited per patient.

Main outcome measures: Family satisfaction measured using the UK FS-ICU-24 questionnaire.

Main Results: 12,346 family members of 6,380 patients were recruited and 7,173 (58%) family members of 4,615 patients returned a completed questionnaire. Overall and domain specific family satisfaction scores were high (mean overall family satisfaction 80, satisfaction with care 83, satisfaction with information 76, and satisfaction with decision-making 73 out of 100) but varied significantly across adult general ICUs studied and by whether the patient survived ICU. For family members of ICU survivors, characteristics of both family member (age, ethnicity, relationship to patient (next-of-kin and/or lived with patient) and visit frequency) and the patient (acute severity of illness and receipt of invasive mechanical ventilation) were significant determinants of family satisfaction, whereas, for family members of ICU non-survivors, only patient characteristics (age, acute severity of illness, and duration of stay) were significant.

Conclusions: Overall family satisfaction in UK adult general ICUs was high but varied significantly. Adjustment for differences in family member/patient characteristics is important to avoid falsely identifying ICUs as outliers.

Study registration: ISRCTN 47363549

For peer review only

For peer review only - http://bmjopen.bmj.com/site/about/guidelines.xhtml

Keywords: critical care; intensive care units; personal satisfaction; family; quality of care; communication

Strengths and limitations of this study

- This is the largest study assessing family satisfaction with ICU care.
- Unbiased selection and stratification of participating units ensured geographical spread (north, south, east, and west England, Wales and Northern Ireland), hospital type (university or non-university) and ICUs of different sizes (large or small – based on number of beds) that recruited for one year to avoid bias from seasonal variation.
- Nesting our study within the Case Mix Programme national clinical audit was efficient and allowed for linkage of family members' to patient data.
- The same mode and timing of delivery of the FS-ICU-24 was employed for family members of ICU survivors and non-survivors, avoiding potential sampling bias and allowing for meaningful comparisons between these groups.
- Despite our very large sample size, we achieved a modest response rate (58%), which was in line with previous published studies.

For peer review only - http://bmjopen.bmj.com/site/about/guidelines.xhtml

Introduction

Humanity of health care, often measured as patient experience, is increasingly seen as one of the three pillars of quality, alongside effectiveness and equity. Eliciting the views and experiences of patients is now seen as essential in delivering a high quality service (1). However, given that approximately 20% of patients admitted to intensive care units (ICUs) die and survivors are often unable to recall their experiences, measuring patient experience in ICU has particular challenges. For this reason, measures of family experience have been developed to help understand the humanity of ICU care.

The most widely validated measure of family experience is the Family Satisfaction in the Intensive Care Unit questionnaire (FS-ICU). This describes satisfaction, overall and in two domains – *satisfaction with care* and *satisfaction with decision making* (2-4). The overall aim of the Family-Reported Experiences Evaluation (FREE) study was to inform the potential routine use of the FS-ICU-24 questionnaire for quality improvement in adult general ICUs in the UK.

This paper reports the results of a large, prospective, multicentre, cohort study describing family satisfaction with ICU care in the UK, investigates how characteristics of patients and their family members impact on family satisfaction, and explores if family satisfaction, varies across ICUs, before and after adjustment for family member and patient characteristics identified as being associated with family satisfaction.

Methods

This large, prospective, multicentre cohort study was nested in the Intensive Care National Audit & Research Centre (ICNARC) Case Mix Programme (CMP) – the national clinical audit of adult general ICUs in England, Wales and Northern Ireland. A stratified sample of 20 ICUs were selected to ensure geographical spread (north, south, east, and west England, Wales and Northern Ireland), hospital type (university or non-university) and ICUs of different sizes (large or small – based on number of beds) and recruited for one year to avoid bias from seasonal variation. In accordance with care standards for UK ICUs at the time of data collection, nurse/patient ratios were 1:1 and 1:2 for Level 3 (Intensive Care) and Level 2 (High Dependency) patients, respectively. The study was reviewed and approved by the National Research Ethics Service Committee South Central - Berkshire B (reference 13/SC/0037) and was registered prospectively (ISRCTN47363549).

For peer review only - http://bmjopen.bmj.com/site/about/guidelines.xhtml

Patient and Public Involvement

Engagement with patient and their family members was vital to ensuring the successful delivery of the FREE study. A former critical care patient and a family member of a former critical care patient were co-investigators on the FREE study and contributed to all aspects of the study including: design; conduct; management; analysis; interpretation of results; and dissemination as members of the study management group. Additionally, the study steering committee included patient and family members.

Recruitment and follow-up

Recruitment and follow-up of family members have been described in detail elsewhere (5). Briefly, a 'family member' was defined as any person with close familial, social or emotional relationship to the patient and was not restricted solely to next-of-kin. Up to four family members of patients who spent ≥24 hours in ICU were eligible to participate if they met the following criteria: aged ≥18 years; had physically visited the patient's bedside at least once after the first 24 hours; had a UK postal address; and had not already been recruited into the study.

Patients were followed-up to ICU discharge. Approximately three weeks after the patient had either been discharged from or died in the ICU, a questionnaire pack was mailed to their recruited and consented family member(s) direct from the ICNARC Clinical Trials Unit. Data from completed questionnaires were entered centrally onto a secure database. All identifiable information such as names (e.g. of patients, family members, and critical care staff members) were removed. Quality checking of entered data was conducted and, for a 20% random sample, accuracy was verified. All fields in the database with missing data were verified against the paper questionnaires.

Statistical analysis

Item responses were rescaled and, where relevant, reversed, according to the developer's rules, so that each response was on a scale from 0 (least satisfied) to 100 (most satisfied) (4). Recent work from our group (6) established the construct validity of the FS-ICU 24-item questionnaire (FS-ICU-24) was improved by using three domains (splitting the *satisfaction with decision making* domain into two – *satisfaction with information* and *satisfaction with decision making process*). Overall family satisfaction score and three domain scores were calculated by averaging the item responses for the relevant items.

Family member and patient characteristics were described by mean and standard deviation (SD), median and quartiles, or number and percentage stratified by the patient outcome (alive/dead).

For peer review only - http://bmjopen.bmj.com/site/about/guidelines.xhtml

Variation in family satisfaction was analysed across the following factors: patient; family member; ICU/hospital (hospital teaching status and number of beds in the ICU); and other contextual. These factors were then explored using univariable and multivariable multilevel linear regression models (7) with a primary outcome of the overall family satisfaction score. In secondary analyses, separate models were fitted for the three individual domains of family satisfaction. Separate models were fitted for family members of ICU survivors and non-survivors. After modelling, the normality of error assumption was assessed by measurements of skewness. Normal probability plots were also used to assess the distribution of residuals at each level. As a sensitivity analysis we ran a multilevel regression model on the square root of the score using the same set of variables to confirm inference. All analyses were conducted in Stata/SE Version 13.0 (StataCorp, College Station, TX).

Variation in family satisfaction across ICUs was assessed graphically using funnel plots, which plot the average family satisfaction score for each critical care unit against the number of family members returning questionnaires. Control limits placed at 2 and 3 SDs around the overall mean indicate the regions of the funnel within which we would expect 95% and 99.8% of points to lie if all variation was due to chance (8).

Due to the natural structure of the data and the planned analysis multilevel multiple imputation (MLMI) was used to complete non- and partial responses for outcomes and family member characteristics. Data were imputed using REALCOM-Impute, an MLwiN 2.15 macro that generates imputations for hierarchical data (9). To test whether our findings were influenced by using imputed data, we also conducted sensitivity analyses using a traditional approach to scoring the FS-ICU-24 by including only responders with ≥60% of items completed.

Results

Of the 210 adult, general ICUs participating in the CMP, 142 (67.6%) expressed an interest in participating and the 20 ICUs were selected using stratified, random sampling. The characteristics and outcomes of all admissions to the study ICUs were similar to admissions to all ICUs in the CMP during the same period (Supplementary Table S1).

Between 28 May 2013 and 30 June 2014, 18,757 patients were admitted to the 20 ICUs, of which 12,730 patients stayed at least 24 hours in the ICU. From these, 12,346 family members of 6380 patients were recruited. Fully or partially completed questionnaires were returned by 7173 family members of 4615 patients. Family members of patients for whom no CMP data were available were not included, so finally, 7019 were included in the final analysis (Supplementary Figure S1).

For peer review only - http://bmjopen.bmj.com/site/about/guidelines.xhtml

Response rates varied by family member characteristics, including; age, gender, ethnicity, level of deprivation (based on residential postcode), level of education, and relationship with the patient. Family members documented in ICU records as next-of-kin were more likely to complete the questionnaire than those who were not, whilst family members for whom English was their first language were more likely to complete the questionnaire than those for whom it was not (Table S2).

A detailed description of the inclusion process, response rates and responders' characteristics has been reported in Family Reported Experiences Evaluation (FREE) study (5). Comparisons of family member and patient characteristics for ICU survivors and non-survivors are presented in Table 1 and Table 2, respectively.

Table 1 Family member characteristics stratified by the patient's ICU outcome

Family member characteristics	All Family members [N=7,019]	Family members of ICU survivors[N=6,149]	Family members of ICU non-survivors [N=870]
Age, mean (SD)	54 (15.1)	54 (15.0)	52 (15.2)
Age group, n (%)			
<30	507 (7.5)	439 (7.4)	68 (8.0)
30-39	701 (10.3)	595 (10.0)	106 (12.5)
40-49	1,423 (21.0)	1,245 (21.0)	178 (21.0)
50-59	1,614 (23.8)	1,406 (23.7)	208 (24.6)
60-69	1,507 (22.2)	1,334 (22.5)	173 (20.4)
70-79	827 (12.2)	747 (12.6)	80 (9.5)
80+	204 (3.0)	171 (2.9)	33 (3.9)
Sex, n (%)			
Male	2,327 (33.5)	2,052 (33.7)	275 (31.9)
Female	4,622 (66.5)	4,034 (66.3)	588 (68.1)
Ethnicity, n (%)			
White	6,555 (94.0)	5,738 (93.9)	817 (94.6)
Asian	138 (2.0)	114 (1.9)	24 (2.8)
Black	54 (0.8)	50 (0.8)	4 (0.5)
Mixed ethnicity or other ethnic group	88 (1.3)	84 (1.4)	4 (0.5)
Not stated	139 (2.0)	124 (2.0)	15 (1.7)
Relationship to patient, n (%) ("I am the patient's…")			
Partner	2,096 (29.9)	1,891 (30.8)	205 (23.6)
Child	654 (9.3)	1,893 (30.8)	346 (39.8)
Parent	2,239 (31.9)	622 (10.1)	32 (3.7)
Sibling	704 (10.0)	624 (10.1)	80 (9.2)
Other relative	969 (13.8)	799 (13.0)	170 (19.5)
Other non-relative	356 (5.1)	319 (5.2)	37 (4.3)
Next-of-kin, n (%)	3,520 (50.2)	3,153 (51.4)	367 (42.3)
Lives with patient, n (%)	2,559 (36.5)	2,311 (37.6)	248 (28.5)
Highest level of education, n (%)			

For peer review only - http://bmjopen.bmj.com/site/about/guidelines.xhtml

NVQ level 1 or 2	1,683 (28.9)	1,465 (28.9)	218 (29.1)
NVQ level 3	1,123 (19.3)	989 (19.5)	134 (17.9)
NVQ level 4 or 5	1,769 (30.4)	1,537 (30.3)	232 (31.0)
Other	1,244 (21.4)	1,080 (21.3)	164 (21.9)
Quintile of deprivation, n (%)			
1 (least deprived)	1,190 (17.1)	1,164 (19.9)	159 (19.4)
2	1,405 (20.2)	1,281 (21.9)	181 (22.1)
3	1,488 (21.4)	1,238 (21.1)	181 (22.1)
4	1,488 (21.4)	1,189 (20.3)	169 (20.7)
5 (most deprived)	1,391 (20.0)	989 (16.9)	128 (15.6)
Distance (km) from home to hospital, median (IQR)	12.4 (5.4 33.6) [6,714]	12 (6, 34)	12 (5, 33)
Previous experience of ICU as a family member, n (%)	1,841 (26.6)	1,641 (27.1)	200 (23.3)
Frequent visitor, n (%)	5,403 (78.9)	4,713 (78.6)	690 (81.2)

NVQ, National Vocational Qualification level 1 or 2, equivalent to GCSE or O-level (school exams taken at age 16); NVQ level 3, equivalent to A-level, AS-level or High School Certificate (school exams taken at age 18); NVQ level 4 or 5, equivalent to degree, Higher degree, Higher National Certificate, Higher National Diploma.

Table 2 Patient characteristics stratified by ICU outcome

**Patient characteristics**	**All patients [N=4,506]**	**ICU survivors [N=4,007]**	**ICU non-survivors [N=499]**
Age, mean (SD)	63 (17.0)	63 (17.3)	68 (13.2)
Age group, n (%)			
<30	254 (5.6)	246 (6.1)	8 (1.6)
30-39	232 (5.1)	223 (5.6)	9 (1.8)
40-49	412 (9.1)	384 (9.6)	28 (5.6)
50-59	643 (14.3)	586 (14.6)	57 (11.4)
60-69	1,100 (24.4)	966 (24.1)	134 (26.9)
70-79	1,159 (25.7)	1,003 (25.0)	156 (31.3)
80+	706 (15.7)	599 (14.9)	107 (21.4)
Sex, n (%)			
Male	2,561 (56.8)	2,264 (56.5)	297 (59.5)
Female	1,945 (43.2)	1,743 (43.5)	202 (40.5)
Ethnicity, n (%)			
White	4,176 (92.7)	3,706 (92.5)	470 (94.2)
Asian or Asian British	81 (1.8)	69 (1.7)	12 (2.4)
Black or black British	42 (0.9)	39 (1.0)	3 (0.6)
Mixed ethnicity or other ethnic group	79 (1.8)	74 (1.8)	5 (1.0)
Not stated	128 (2.8)	119 (3.0)	9 (1.8)
Quintile of deprivation, n (%)			
1 (least deprived)	774 (17.3)	690 (17.4)	84 (17)
2	905 (20.3)	812 (20.4)	93 (18.8)
3	928 (20.8)	822 (20.7)	106 (21.4)
4	950 (21.3)	841 (21.2)	109 (22)

For peer review only

For peer review only - http://bmjopen.bmj.com/site/about/guidelines.xhtml

5 (most deprived)	912 (20.4)	809 (20.4)	103 (20.8)
Distance (km) from home to hospital, median (IQR)	33.1 (67.8) 9.3 (4.3 19.9) [4,475]	10 (4, 20)	8 (4, 16)
APACHE II severe co-morbidities, n (%)			
Liver	124 (2.8)	94 (2.3)	30 (6.0)
Renal	108 (2.4)	97 (2.4)	11 (2.2)
Respiratory	146 (3.2)	119 (3.0)	27 (5.4)
Cardiovascular	117 (2.6)	100 (2.5)	17 (3.4)
Metastatic cancer	121 (2.7)	110 (2.7)	11 (2.2)
Haematological malignancy	103 (2.3)	81 (2.0)	22 (4.4)
Immunocompromise	369 (8.2)	318 (7.9)	51 (10.2)
Prior dependency, n (%)			
Able to live without assistance	3,267 (72.5)	2,944 (73.5)	323 (64.7)
Minor or major assistance	1,171 (26.0)	1,004 (25.1)	167 (33.5)
Total assistance	47 (1.0)	42 (1.0)	5 (1.0)
Unknown	21 (0.5)	17 (0.4)	4 (0.8)
Surgical status *n* (%)			
Non-surgical	2,808 (62.3)	2,396 (59.8)	412 (82.6)
Planned admission following elective or scheduled surgery	702 (15.6)	686 (17.1)	16 (3.2)
Unplanned admission following surgery of any urgency	996 (22.1)	925 (23.1)	71 (14.2)
ICNARC Physiology Score, mean (SD)	18 (8.3)	18 (7.9)	26 (8.1)
APACHE II Score, mean (SD)	17 (6.3)	16 (6.1)	21 (6.2)
ICU length of stay (days), median (IQR)	4.9 (2.9 9.1)	4.8 (2.8, 9.0)	6.0 (3.6, 10.6)
Organ support received in the ICU, n (%)			
Advanced respiratory support	2,540 (56.4)	2,124 (53.0)	416 (83.4)
Advanced cardiovascular support	1,325 (29.4)	1,037 (25.9)	288 (57.7)
Renal support	691 (15.3)	510 (12.7)	181 (36.3)
Neurological support [a]	617 (13.7)	503 (12.6)	114 (22.8)
Duration (calendar days) of organ support among those receiving the support, median (IQR)			
Advanced respiratory support	5.0 (2.0 9.0)	4 (2, 9)	6 (4, 10)
Advanced cardiovascular support	3.0 (2.0 4.0)	2 (2, 4)	3 (2, 5)
Renal support	4.0 (3.0 8.0)	4 (3, 8)	4 (3, 8)
Neurological support	3.0 (2.0 7.0)	3 (2, 7)	3 (2, 5)
Death before acute hospital discharge, n (%)	852 (19.2)	353 (8.9)	N/A

[a] including admission receiving invasive neurological monitoring or treatment, continuous intravenous medication for seizures and/or cerebral monitoring, and therapeutic hypothermia using protocols and devices

For peer review only

Both overall and individual domain scores revealed generally high satisfaction (Table 3), however a long tail was present indicating some questionnaires were returned with very low scores (Figure 1). Family members of ICU non-survivors had higher scores for overall satisfaction and satisfaction with the decision-making process domain than family members of ICU survivors.

Table 3 Overall family satisfaction score for all family members and for family members by patient outcome

Summary measures	All family members [N=7,017[a]]	Family members of ICU survivors [N=6,147[a]]	Family members of ICU non-survivors [N=870]
Overall family satisfaction score			
**Median [IQR]**	83.3 [70.4, 93.0]	82.7 [69.9, 92.7]	87.1 [74.4, 94.8]
**Mean (SD)**	79.7 (16.7)	79.3 (16.5)	82.0 (17.5)
**[95% CI]**	[79.2 - 80.1]	[78.9 - 79.8]	[80.9 - 83.2]
Satisfaction with care domain score			
**Median [IQR]**	87.5 [74.3, 96.4]	87.5 [73.6, 96.4]	88.1 [76.8, 96.4]
**Mean (SD)**	83.1 (16.0)	83.0 (15.9)	83.8 (16.9)
**[95% CI]**	[82.7 - 83.4]	[82.6 - 83.4]	[82.7 - 84.9]
Satisfaction with information domain score			
**Median [IQR]**	79.2 [66.7, 95.8]	79.2 [62.5, 95.8]	83.3 [70.8, 100.0]
**Mean (SD)**	76.2 (22.0)	75.7 (22.0)	79.6 (22.9)
**[95% CI]**	[75.7 - 76.7]	[75.1 - 76.2]	[78.1 - 81.0]
Satisfaction with the decision-making process domain score			
**Median [IQR]**	75.6 [59.3, 93.1]	75.0 [57.5, 88.8]	87.5 [68.8, 100.0]
**Mean (SD)**	73.1 (22.3)	72.1 (22.0)	79.6 (22.9)
**[95% CI]**	[72.5 - 73.6]	[71.6 - 72.7]	[78.1 - 81.1]

[a] Two family members returned questionnaires but did not complete any of the 24 FS-ICU items – responses were not imputed for these family members.

For peer review only - http://bmjopen.bmj.com/site/about/guidelines.xhtml

Univariable analyses of the association between family satisfaction and family characteristics, patient characteristics, ICU/hospital characteristics and contextual factors are shown in the Supplementary Appendix (Table S3-S5). Family member level and patient level variables that were statistically significant along with the a priori key family member/patient variables (age, sex), were carried forward to the multivariable multilevel modelling process (5). There was no evidence of differences in family satisfaction according to hospital teaching status or the number of beds in the ICU, however, these variables were retained in the multilevel multivariable models due to their controlling effect on the other coefficients in the models. A summary of the candidate considered in the models and a justification of their inclusion/exclusion is detailed in Table S6.

Results of the multivariable multilevel models for overall family satisfaction are shown in

Table 4. Among family members of ICU survivors, there was evidence of an association with overall family satisfaction for: family member age group; family member ethnicity; next-of-kin/lives with patient; frequency of visits; ICNARC Physiology Score; and receipt of advanced respiratory support. Among family members of non-survivors, only the following patient factors were significant: patient age; ICNARC Physiology Score; and ICU length of stay. These associations were significant when controlling for other predictors in the model. A priori-specified interaction terms and random slopes did not improve the fit of the models and so these terms were not retained.

Table 4 Multivariable multilevel models for overall family satisfaction score

**Variables**	**Family members of ICU survivors [N=6,143[a]]**			**Family members of ICU non-survivors [N=869[a]]**		
	Coef.	95% CI	p-value	Coef.	95% CI	p-value
Fixed effects – family member level						
Constant	68.30	(63.42, 73.17)		55.70	(42.26, 69.14)	
Family member age, years (vs <30)			0.041			0.18
30-39	1.97	(0.11, 3.82)		2.01	(-2.64, 6.66)	
40-49	1.65	(0.02, 3.29)		3.37	(-1.01, 7.75)	
50-59	1.96	(0.35, 3.56)		4.12	(-0.09, 8.33)	
60-69	1.35	(-0.31, 3.01)		4.26	(-0.25, 8.79)	
70-79	1.32	(-0.52, 3.17)		5.92	(0.69, 11.14)	
80+	-1.34	(-4.06, 1.37)		-0.18	(-6.80, 6.43)	
Family member sex – female (vs male)	0.32	(-0.48, 1.12)	0.44	0.66	(-1.45, 2.77)	0.54
Family member ethnicity – white (vs non-white)	3.59	(1.38, 5.80)	0.001	7.12	(-0.00, 14.25)	0.050

For peer review only - http://bmjopen.bmj.com/site/about/guidelines.xhtml

Next-of-kin/lives with patient (vs lives with patient)			<0.001			0.26
Next-of-kin, does not live with patient	-1.39	(-2.56, -0.22)		1.08	(-2.39, 4.55)	
Not next-of-kin, does not live with patient	-2.33	(-3.26, -1.41)		-1.24	(-3.88, 1.40)	
Frequent visitor	2.83	(1.82, 3.84)	<0.001	1.53	(-1.34, 4.39)	0.30
Fixed effects – patient level						
Patient age (per 10 years)	0.01	(-0.28, 0.31)	0.93	1.18	(0.09, 2.27)	0.033
Patient sex – female (vs male)	0.26	(-0.73, 1.25)	0.61	1.92	(-0.85, 4.70)	0.17
Dependency (vs none)			0.15			0.74
Minor or major	-0.30	(-1.60, 1.00)		-0.22	(-3.36, 2.92)	
Total	-4.62	(-9.32, 0.07)		4.98	(-8.10, 18.07)	
Surgical status (vs non-surgical)			0.63			0.82
Planned elective/scheduled	-0.74	(-2.24, 0.77)		-2.61	(-10.77, 5.54)	
Unplanned	-0.26	(-1.46, 0.94)		-0.08	(-3.95, 3.80)	
ICNARC Physiology Score (per point)	0.16	(0.09, 0.24)	<0.001	0.17	(0.00, 0.34)	0.045
ICU length of stay (per day)	-0.02	(-0.07, 0.03)	0.44	-0.30	(-0.46, -0.15)	<0.001
Advanced respiratory support	2.96	(1.80, 4.11)	<0.001	---		
Fixed effects – ICU/hospital level						
Hospital type (vs non-university)			0.49			0.55
University	0.86	(-3.61, 5.32)		-1.51	(-7.51, 4.50)	
University affiliated	1.97	(-1.26, 5.20)		1.77	(-2.55, 6.09)	
Number of ICU beds (per bed)	-0.00	(-0.23, 0.23)	0.97	0.26	(-0.08, 0.61)	0.13
Random effects – SD (SE)						
Between ICUs	2.91	(0.60)		2.81	(1.10)	
Within ICUs between patients	10.94	(0.29)		11.16	(0.69)	
Within patients between family members	11.98	(0.21)		12.26	(0.44)	
Variance partition – percentage						
Between ICUs	3%			2%		
Between patients	44%			44%		

Coef, coefficient; SE, standard error.

[a]Five patients were missing age group on both the questionnaire and web portal – due to the very small amount of missing data in this key variable, these missing values were not imputed.

Variances at both the patient and ICU/hospital levels were statistically significant but the variance partition coefficients (VPCs) at the ICU/hospital level were small in both the null and final multilevel models (4% and 3% for ICU survivors and 2% and 2% for ICU non-survivors, respectively), which

For peer review only - http://bmjopen.bmj.com/site/about/guidelines.xhtml

means differences in overall family satisfaction scores were mainly at the patient and family member levels. Variance at the patient level represented 44% of the total variance in overall family satisfaction in the final models for family members of both ICU survivors and ICU non-survivors.

Full results of the multivariable multilevel models for the domain scores are reported in the Supplementary Appendix (Table S7-S9).

Figure 2 shows the funnel plots for the overall family satisfaction score, before and after adjustment for family member and patient characteristics from the multivariable multilevel models. Adjusting for family member and patient characteristics reduced the variability across ICUs, resulting in fewer ICUs outside the funnel plot control limits. Funnel plots for the individual domain scores before and after adjustment for can be found in the Supplementary Appendix (Figure S2).

Sensitivity analyses

Multivariable multilevel models using the square root transformation of the satisfaction scores gave consistent results. In the models using imputed data, the direction and order of magnitude of coefficients that were significant were similar to those estimated using the traditional approach to scoring partially completed questionnaires (Supplementary Appendix, Table S10 and Table S11). On average, the multiple imputation approach tended to identify larger numbers of potential outliers due to the larger sample sizes and therefore narrower funnels.

Discussion

Overall and domain specific family satisfaction measured with the UK FS-ICU-24 was high. However, we found that it varies significantly across adult general ICUs and that family members of patients who died in the ICU had higher levels of satisfaction. For family members of ICU survivors, characteristics of both family member and the patient were significant determinants of family satisfaction, whereas, for family members of ICU non-survivors, only patient characteristics were significant. Adjustment for these family member and patient characteristics reduced the variation in family satisfaction across ICUs, resulting in fewer ICUs being identified as outliers.

The overall satisfaction score was comparable with other published studies employing similar methods to administer the FS-ICU-24 (10-13). Our findings are also consistent with a study by Wall et al (14) which identified that families of ICU non-survivors were more satisfied than families of ICU survivors. Similarly, Stricker et al (15) found that increasing acute severity of illness of the patient (evaluated using the SAPS II score) was associated with increasing satisfaction on the overall family

For peer review only - http://bmjopen.bmj.com/site/about/guidelines.xhtml

satisfaction score, however, lower satisfaction was associated with ICU-level characteristics of a written admission/discharge policy and a higher patient:nurse ratio. Other considered patient characteristics were found not to be significant.

It is of note that one of largest magnitude associations in the FREE study was the finding that white family members of both ICU survivors and non-survivors had higher satisfaction, on average, than those of other ethnicities. Further investigation of this issue is warranted to understand whether this reflects, for example, either cultural variation in family members' expectations or a need to engage better and communicate with family members who may not have English as their first language (17% of family members of non-white ethnicity indicated that their first language was not English compared with less than 1% of those of white ethnicity).

Our work has several important strengths. To our knowledge, this is the largest study assessing family satisfaction with ICU care. Nesting our study within the national clinical audit programme was efficient and novel and allowed for unbiased selection and stratification of participating units and linkage of family members' to patient data. One important strength is that the same mode and timing of delivery of the FS-ICU-24 was employed for family members of ICU survivors and non-survivors, avoiding potential sampling bias and allowing for meaningful comparisons between these groups. Finally, the large sample size of family members allowed for robust multilevel multivariable modelling of factors associated with overall family satisfaction to inform important adjustment of any future assessment using this questionnaire. Despite our very large sample size, we achieved a modest response rate (58%), however this was similar to other studies with smaller sample sizes (10, 14).

Our study does, however, have limitations. Firstly, when assessing satisfaction, it is not uncommon for continuous measures to be skewed. Whilst the skewed nature of the satisfaction scores does not affect the parameter estimates in multilevel models (16, 17) it might cause problems when one is interested in the significance or in the confidence intervals of the variance terms at higher levels (17). In our analyses, we corrected the asymptotic standard errors using a robust (Huber/White) estimator to improve inference and performed a sensitivity analysis using a square root transformation which did not change our conclusions. Secondly, by excluding family members of patients who had spent less than 24 hours on ICU - to ensure that family members had spent long enough on ICU to feel able to respond to the questionnaire - we may have missed a small group of very sick patients who die soon after admission to ICU. Thirdly, there were differences in the case mix and outcome of patients between those who had at least one family member recruited and those who did not, leading to potential bias in the results. Fourthly, we found that younger family

For peer review only - http://bmjopen.bmj.com/site/about/guidelines.xhtml

members and those from ethnic minority groups were less likely to respond and important information may have been missed. Finally, 94% of patients were of white ethnicity, which is above that of the ethnic make-up of the UK (87%) and may make the overall family satisfaction scores less generalisable to other ethnicities.

In conclusion, this large, prospective, multicentre cohort study indicated that overall family satisfaction with adult general ICU care in the UK was high. However, adjustment for differences in family member/patient characteristics are important to avoid falsely identifying ICUs as outliers.

For peer review only

For peer review only - http://bmjopen.bmj.com/site/about/guidelines.xhtml

Acknowledgements

We wish to thank the NIHR Health Services and Delivery Research programme for funding this project. We also wish to thank all the patients, family members and staff from all the units that participated the study.

Research staff at sites

C Smalley and R Jacob (Arrowe Park Hospital); S Chau, S A Pearson, K Ellis and R Watmough (Barnsley Hospital); M Faulkner, L Evans and H Robertson (Countess of Chester Hospital); P Wakefield, R Abrahams, N Summers and H Wooldridge (Darent Valley Hospital); H McMillan, S Tyson, K Tantam, S Olver, C Brown and C Tippett (Derriford Hospital); S Moreton, S Jones, A Deeney, J Gibbins and A Oglesby (Dorset County Hospital); C Randell, M Allsop, K Harris, C Scott and C Boyd (Freeman Hospital); E Coughlan, A Jefferies and K Wylie (Manchester Royal Infirmary); C Plowright, C Pegg, L Cooper and T Hatton (Medway Maritime Hospital); P Doble, P Richards, D Bayford and K Adams (Musgrove Park Hospital); J Spimpolo, M Burt and R Pillai (Northampton General Hospital); K A Simeson and S Buckley (Pinderfields Hospital); A Jackson, M Nadolski and H Baker (Royal Devon & Exeter Hospital, Wonford); N Mason, U Gunter and L Roberts (Royal Gwent Hospital); T Evans, E Cooke, M Ogden and P Dark (Salford Royal Hospital); M Cody, F Hogg and D McCahery (South West Acute Hospital); D Dawson, J Mellinghoff, S Prudden, N Poonuth and C Ryan (St George's Hospital); G Mandersloot, A Smith (The Royal London Hospital); S Hagan, L Humphries and E Murphy (Ulster Hospital); E Walker, H Payne and X Zhao (Watford General Hospital); C Edmondson, S Anglesea and H Williams (Wrexham Maelor Hospital).

Study Steering Committee

Dr Kathleen Daly (independent chair); Andrina Colquoun (independent); Dr Maureen Dalziel; Kirsty Everingham (independent); Doreen Henry (independent); Joan Pearson (independent); Catherine Plowright; Dr Laura Price (independent); Professor Kathryn Rowan; Professor Mervyn Singer (independent); and Dr Stephen Wright.

Funding: This project was funded by the National Institute for Health Research (NIHR) Health Services and Delivery Research (HS&DR) Programme (11/2003/56). The funder had no involvement in study design; in collection, analysis and interpretation of data; in the writing of the report; or in the decision to submit the article for publication. The views and opinions expressed therein are those of the authors and do not necessarily reflect those of the HS&DR Programme, NIHR, NHS or the Department of Health.

For peer review only - http://bmjopen.bmj.com/site/about/guidelines.xhtml

Competing interests: Kathryn M Rowan is a member of the NIHR HS&DR Board. Elaine McColl was an editor for the NIHR Journals Library between 2013 and 2016 and received a fee for this work. The other authors declare no conflicts of interest. All authors have completed the Unified Competing Interest form (available on request from the corresponding author).

Data sharing: data can be obtained from the corresponding author on request

Authors contributions: KMR as Chief Investigator conceived the idea and designed the study with DAH, SHE, DKH, LH, EMc, MR, AR, and SEW. EW co-ordinated the study and contributed to data acquisition with ARB, RRC, SS, SHE, AR, and SEW. PFV, DWG, DAH, SHE, DKH, LH, EMc, MR, SEW, and KMR were involved in the analysis and interpretation of the results. All authors were involved in drafting, editing and have approved the final manuscript.

For peer review only

For peer review only - http://bmjopen.bmj.com/site/about/guidelines.xhtml

References

1. Black N, Jenkinson C. Measuring patients' experiences and outcomes. BMJ. 2009;339:b2495.
2. Heyland DK, Tranmer JE. Measuring family satisfaction with care in the intensive care unit: the development of a questionnaire and preliminary results. J Crit Care. 2001;16(4):142-9.
3. Rothen HU, Stricker KH, Heyland DK. Family satisfaction with critical care: measurements and messages. Curr Opin Crit Care. 2010;16(6):623-31.
4. Wall RJ, Engelberg RA, Downey L, et al. Refinement, scoring, and validation of the Family Satisfaction in the Intensive Care Unit (FS-ICU) survey. Crit Care Med. 2007;35(1):271-9.
5. Wright SE, Walmsley E, Harvey SE, et al. Family-Reported Experiences Evaluation (FREE) study: a mixed-methods study to evaluate families' satisfaction with adult critical care services in the NHS. Health Serv Deliv Res. 2015;3(45).
6. DA H, P F-V, SE W, et al. Psychometric assessment of the Family Satisfaction in the Intensive Care Unit questionnaire in the United Kingdom. J Crit Care. 2017;38:346-50.
7. Snijders TAB, Bosker RJ. Multilevel Analysis: An Introduction to Basic and Advanced Multilevel Modeling. Thousand Oaks, CA: SAGE Publications; 1999.
8. Spiegelhalter DJ. Funnel plots for comparing institutional performance. Stat Med. 2005;24(8):1185-202.
9. Carpenter JR, Goldstein H, Kenward MG. REALCOM-IMPUTE software for multilevel multiple imputation with mixed response types. Journal of Statistical Software. 2011;45:5.
10. Dodek PM, Wong H, Heyland DK, et al. The relationship between organizational culture and family satisfaction in critical care. Crit Care Med. 2012;40(5):1506-12.
11. Khalaila R. Patients' family satisfaction with needs met at the medical intensive care unit. Journal of Advanced Nursing. 2013(69):1172-82.
12. Schwarzkopf D, Behrend S, Skupin H, et al. Family satisfaction in the intensive care unit: a quantitative and qualitative analysis. Intensive Care Med. 2013;39(6):1071-9.
13. Tastan S, Iyigun E, Ayhan H, et al. Validity and reliability of Turkish version of family satisfaction in the intensive care unit. Int J Nurs Pract. 2014;20(3):320-6.
14. Wall RJ, Curtis JR, Cooke CR, et al. Family satisfaction in the ICU: differences between families of survivors and nonsurvivors. Chest. 2007;132(5):1425-33.
15. Stricker KH, Kimberger O, Schmidlin K, et al. Family satisfaction in the intensive care unit: what makes the difference? Intensive Care Med. 2009;35(12):2051-9.
16. Gelman A HJ. Data Analysis Using Regression and Multilevel/Hierarchical Models. Cambridge: Cambridge University Press; 2007.
17. Maas CJM, Hox JJ. The influence of violations of assumptions on multilevel parameter estimates and their standard errors. Computational Statistics & Data Analysis. 2004;46(3):427-40.

For peer review only

For peer review only - http://bmjopen.bmj.com/site/about/guidelines.xhtml

Figure legends

Figure 1 Distribution of overall family satisfaction score

Figure 2 Variation across ICUs in the mean overall family satisfaction score (A) before and (B) after adjustment for patient and family member characteristics

For peer review only

For peer review only - http://bmjopen.bmj.com/site/about/guidelines.xhtml

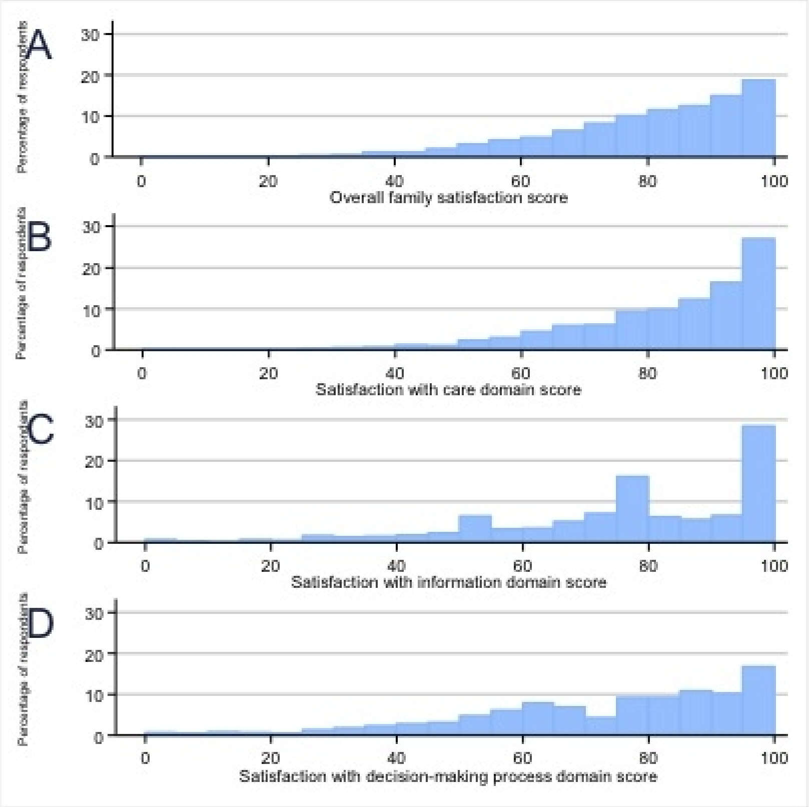

Figure 1 Distribution of overall family satisfaction score

145x145mm (72 x 72 DPI)

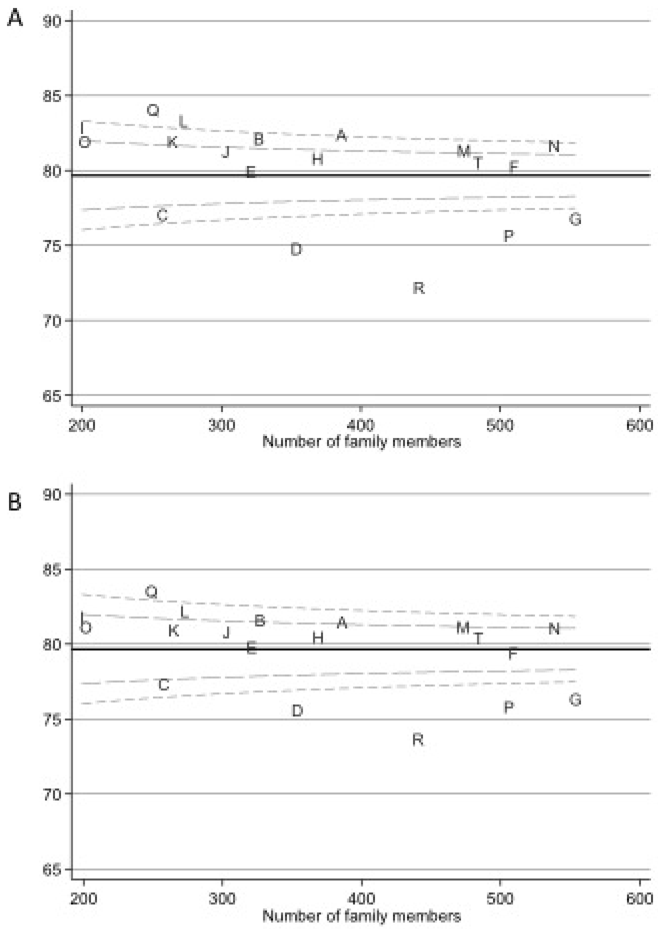

Figure 2 Variation across ICUs in the mean overall family satisfaction score (A) before and (B) after adjustment for patient and family member characteristics

120x172mm (72 x 72 DPI)

Supplementary material

Family satisfaction with critical care in the United Kingdom: a multi-centre cohort study

For peer review only

Table S1 Characteristics and outcomes for all admission to ICUs participating in the FREE study and ICNARC Case Mix Programme

	**CMP**	**FREE study**
Total number of ICUs [N]	[209][a]	[19][a]
Total number of admissions [N]	[149,779]	[18,270]
Age *mean* (SD)	61.5 (18.0)	61.5 (18.0)
Sex *male* (%)	82,444 (55.0)	10,316 (56.5)
Ethnicity *n* (%)		
White	135,767 (90.6)	16,439 (90.0)
Asian	4,815 (3.2)	439 (2.4)
Black	3,250 (2.2)	327 (1.8)
Other	2,434 (1.6)	445 (2.4)
Not stated	3,513 (2.3)	620 (3.4)
Distance (km) from patient home to hospital *median* (IQR) [N]	25.0 (54.2) 8.7 (3.9 19.3) [128,169]	31.7 (64.5) 9.2 (4.2 20.8) [18,090]
APACHE II severe co-morbidities *n* (%)		
0	123,437 (82.4)	14,742 (80.7)
1	20,906 (14.0)	2,648 (14.5)
2	5,053 (3.4)	793 (4.3)
3 or more	383 (0.3)	87 (0.5)
Admission type *n* (%) [N]	[149,765]	[18,270]
Medical	87,940 (58.7)	10,039 (54.9)
Elective surgery	34,284 (22.9)	4,761 (26.1)
Emergency surgery	27,541 (18.4)	3,470 (19.0)
Surgical status of surgical admissions *n* (%) [N]	[61,825]	[8,231]
Planned surgery	28,267 (45.7)	3,985 (48.4)
Unplanned surgery	33,558 (54.3)	4,246 (51.6)
ICNARC Physiology Score *mean* (SD)	16.9 (9.3)	16.5 (9.2)
ICNARC predicted risk of death *median* (IQR) [N]	0.10 (0.03 0.33) [142,654]	0.09 (0.03 0.30) [17,261]
APACHE II Acute Physiology Score *mean* (SD)	11.4 (6.1)	11.3 (5.9)
APACHE II Score *mean* (SD)	15.7 (7.0)	15.6 (6.9)
APACHE II predicted risk of death *median* (IQR) [N]	0.12 (0.04 0.29) [132,197]	0.11 (0.04 0.28) [16,193]
Mechanical ventilation during first 24 hrs *n* (%) [N]	58,687 (39.4) [148,975]	7,008 (38.5) [18,187]

For peer review only

ICU mortality *n* (%) [N]	21,505 (14.4) [149,779]	2,560 (14.0) [18,270]
Acute hospital mortality *n* (%) [N]	29,945 (21.0) [142,670]	3,550 (20.6) [17,266]

[a] excludes one ICU for which no CMP data were available

For peer review only

Figure S1 Overview of patients, family members and questionnaires (distributed/returned)

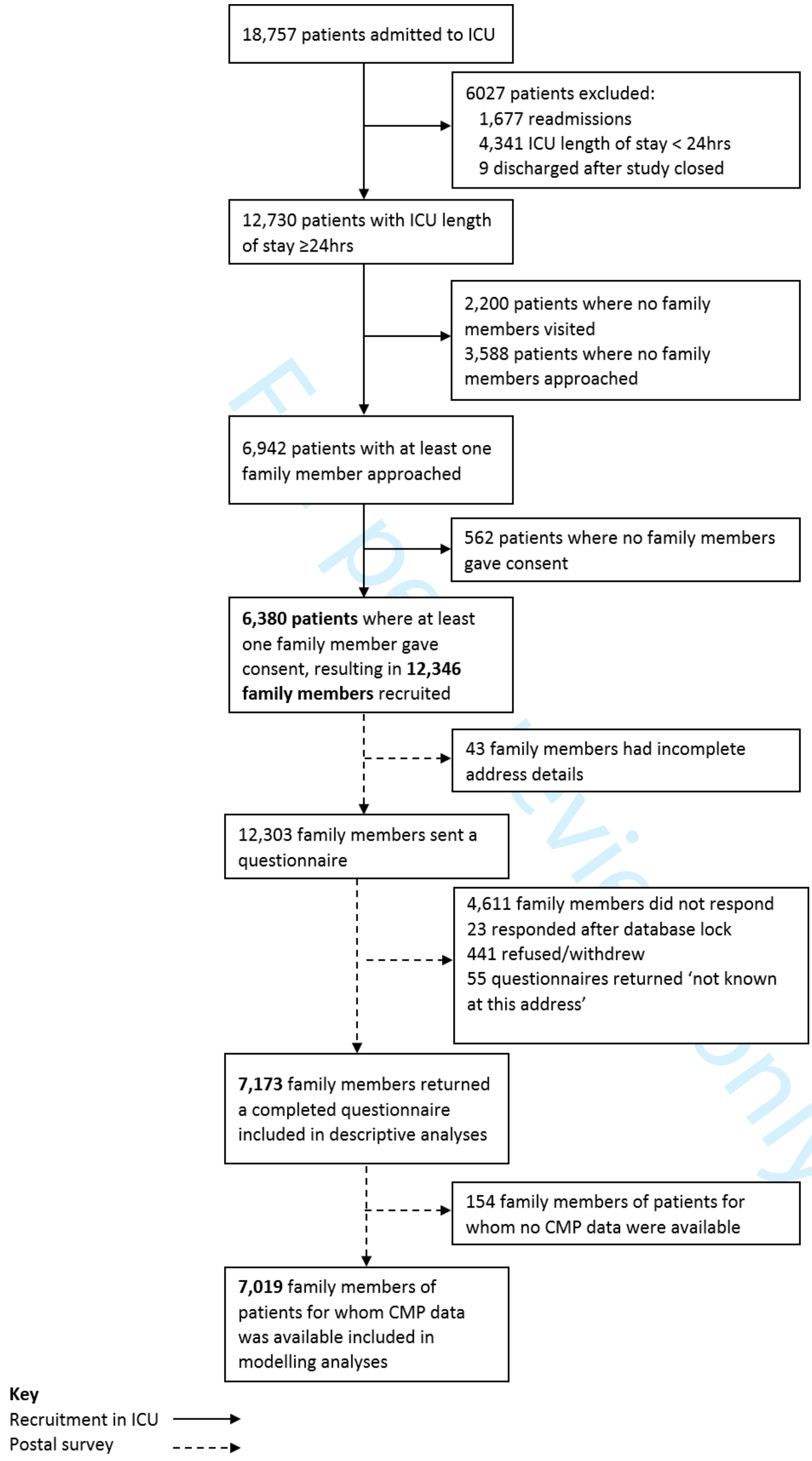

For peer review only

Table S2 Characteristics of all recruited family members and by response to questionnaire

	All recruited family members	Those returning questionnaires	Did not respond
Total number of family members, N	12 346	7173	4611
Age group, *n* (%) [N]	[12 068]	[7019]	[4500]
<30	1429 (11.8)	530 (7.6)	861 (19.1)
30-39	1590 (13.2)	721 (10.3)	827 (18.4)
40-49	2760 (22.9)	1465 (20.9)	1208 (26.9)
50-59	2646 (21.9)	1654 (23.6)	886 (19.7)
60-69	2131 (17.7)	1580 (22.5)	440 (9.8)
70-79	1211 (10.0)	862 (12.3)	220 (4.8)
80+	301 (2.5)	207 (2.9)	58 (1.3)
Sex, *n* (%) [N]	[12 145]	[7062]	[4529]
Female	7687 (63.3)	4689 (66.4)	2663 (58.8)
Male	4458 (36.7)	2373 (33.6)	1866 (41.2)
Ethnicity, *n* (%) [N]	[12 090]	[7033]	[4505]
White	11 379 (94.1)	6747 (95.9)	4111 (91.3)
Asian	355 (2.9)	142 (2.0)	196 (4.4)
Black	161 (1.3)	55 (0.8)	101 (2.2)
Other	195 (1.6)	89 (1.3)	97 (2.1)
Deprivation, *n* (%) [N]	[11 740]	[6832]	[4370]
1 [least deprived]	2113 (18.0)	1376 (20.1)	634 (14.5)
2	2406 (20.5)	1502 (22.0)	803 (18.4)
3	2415 (20.6)	1443 (21.1)	851 (19.5)
4	2545 (21.7)	1380 (20.2)	1045 (23.9)
5 [most deprived]	2261 (19.3)	1131 (16.6)	1037 (23.7)
Distance (km) from family member home to hospital, *median* (IQR) [N]	11.6 (5.1-30.7) [11 803]	12.3 (5.3-33.2) [6867]	10.7 (4.6-29.4) [4394]
Relationship, *n* (%) [N] "I am the patient's..."	[12 343]	[7173]	[4611]
Partner	3105 (25.2)	2151 (30.0)	786 (17.0)
Child	4186 (33.9)	2292 (32.0)	1780 (38.6)
Parent	1054 (8.5)	665 (9.3)	338 (7.3)
Sibling	1271 (10.3)	717 (10.0)	480 (10.4)
Other relative	1973 (16.0)	987 (13.8)	898 (19.5)
Other non-relative	754 (6.1)	361 (5.0)	329 (7.1)
Next-of-kin, *n* (%) [N]	[11 702]	[6770]	[4389]
No	7086 (60.6)	3747 (55.3)	3009 (68.6)
Yes	4616 (39.4)	3023 (44.7)	1380 (31.4)
Lives with patient, *n* (%) [N]	[12 343]	[7172]	[4609]
No	8255 (66.9)	4543 (63.3)	3357 (72.8)
Yes	4088 (33.1)	2629 (36.7)	1252 (27.2)
Education level, *n* (%) [N]	[10 293]	[5971]	[3888]
NVQ 1 or 2	3147 (30.6)	1731 (29.0)	1284 (33.0)
NVQ 3	2086 (20.3)	1149 (19.2)	870 (22.4)

For peer review only

NVQ 4 or 5	2936 (28.5)	1819 (30.5)	1032 (26.5)
Other	2124 (20.6)	1272 (21.3)	702 (18.1)
First language, *n* (%) [N]	[12 346]	[7 173]	[4611]
Not English	335 (2.7)	140 (2.0)	182 (3.9)
English	12 011 (97.3)	7 033 (98.0)	4429 (96.1)

For peer review only

Table S3 Univariable analyses of factors associated with overall family satisfaction score by ICU outcome – family member characteristics

Variables	Family members of ICU survivors [N=6,147[a]]			Family members of ICU non-survivors [N=870]		
	Coef.	95% CI	p-value	Coef.	95% CI	p-value
Age, years (vs < 30)			0.031			0.033
30-39	1.56	(-0.22, 3.33)		2.68	(-1.80, 7.17)	
40-49	0.42	(-0.10, 0.94)		1.61	(0.21, 3.01)	
50-59	2.12	(0.61, 3.64)		5.49	(1.49, 9.50)	
60-69	1.96	(0.39, 3.52)		6.01	(1.78, 10.25)	
70-79	1.98	(0.28, 3.68)		7.39	(2.58, 12.19)	
80+	-0.55	(-3.05, 1.95)		2.62	(-3.48, 8.73)	
Female (vs male)	0.40	(-0.34, 1.14)	0.29	0.44	(-1.59, 2.47)	0.67
White ethnicity (vs non-white)	3.60	(1.46, 5.75)	0.001	8.78	(1.85, 15.70)	0.013
Relationship (vs partner)			<0.001			0.28
Parent	0.00	(-1.39, 1.39)		0.08	(-5.73, 5.90)	
Child	-0.94	(-1.83, -0.05)		-1.274	(-3.69, 1.14)	
Sibling	-2.16	(-3.50, -0.82)		0.909	(-3.02, 4.84)	
Other-relative	-1.63	(-2.81, -0.44)		-0.619	(-3.60, 2.36)	
Other-non relative	-3.42	(-5.22, -1.62)		-6.134	(-11.69, -0.58)	
Next of kin	1.74	(1.05, 2.44)	<0.001	2.69	(0.78, 4.59)	0.006
Lives with patient	1.95	(1.20, 2.69)	<0.001	1.15	(-0.99, 3.29)	0.29
Education (vs NVQ 1 or 2)			<0.001			0.16
NVQ 3	-0.60	(-1.77, 0.57)		1.14	(-2.09, 4.37)	
NVQ 4 or 5	-2.43	(-3.49, -1.37)		-2.07	(-4.92, 0.77)	
Other	-0.18	(-1.35, 0.98)		-1.75	(-4.73, 1.24)	
Quintile of deprivation (vs 1, least deprived)			0.63			0.77
2	0.49	(-0.74, 1.72)		0.64	(-2.73, 4.01)	
3	0.96	(-0.29, 2.20)		0.84	(-2.59, 4.26)	
4	0.32	(-0.97, 1.60)		-1.07	(-4.59, 2.44)	
5 (most deprived)	0.67	(-0.70, 2.05)		0.79	(-3.10, 4.69)	
Distance from home to hospital (per 10 km)	-0.05	(-0.11, 0.01)	0.12	0.05	(-0.09, 0.18)	0.49
Previous experience of ICU as a family member	0.25	(-0.63, 1.14)	0.58	-0.68	(-3.22, 1.87)	0.60
Frequent visitor	2.52	(1.63, 3.41)	<0.001	2.91	(0.36, 5.47)	0.030

Coef., coefficient.

[a] Two family members returned questionnaires but did not complete any of the 24 FS-ICU items – responses were not imputed for these family members.

For peer review only

Table S4 Univariable analyses of factors associated with overall family satisfaction score by ICU outcome – patient characteristics

Variables	Family members of ICU survivors [N=6,147[a]]			Family members of ICU non-survivors [N=870]		
	Coef.	95% CI	p-value	Coef.	95% CI	p-value
Age (per 10 years)	-0.09	(-0.36, 0.17)	0.49	1.12	(0.11, 2.14)	0.030
Female (vs male)	0.67	(-0.25, 1.59)	0.16	2.04	(-0.66, 4.74)	0.14
White ethnicity (vs non-white)	2.39	(0.11, 4.68)	0.040	9.25	(2.38, 16.12)	0.008
Quintile of deprivation (vs 1, least deprived)			0.76			0.95
2	0.86	(-0.66, 2.38)		-1.28	(-5.85, 3.29)	
3	0.62	(-0.90, 2.13)		-0.68	(-5.12, 3.75)	
4	0.77	(-0.75, 2.28)		-1.62	(-6.03, 2.78)	
5 (most deprived)	1.00	(-0.57, 2.57)		-1.49	(-6.04, 3.06)	
Distance from home to hospital (per 10 km)	0.12	(0.00, 0.24)	0.047	0.18	(-0.05, 0.41)	0.12
Severe comorbidities						
Liver	3.18	(-0.01, 6.38)	0.050	1.25	(-4.67, 7.19)	0.68
Renal	-0.45	(-3.57, 2.66)	0.77	-8.87	(-18.35, 0.60)	0.067
Respiratory	0.01	(-2.84, 2.85)	1.00	-1.02	(-7.23, 5.19)	0.75
Cardiovascular	-0.14	(-3.23, 2.94)	0.93	1.40	(-6.46, 9.26)	0.73
Metastatic cancer	-2.81	(-5.78, 0.15)	0.063	3.26	(-6.38, 12.90)	0.51
Haematological malignancy	2.25	(-1.09, 5.61)	0.19	-7.88	(-14.62, -1.13)	0.022
Immunocompromise	-0.91	(-2.74, 0.90)	0.33	-3.90	(-8.55, 0.74)	0.10
Dependency (vs none)			0.30			0.85
Minor or major	-0.14	(-1.36, 1.08)		0.63	(-2.34, 3.60)	
Total	-3.63	(-8.21, 0.94)		2.73	(-10.21, 15.67)	
Surgical status (vs non-surgical)			0.005			0.78
Planned elective/scheduled	-2.17	(-3.51, -0.83)		-2.83	(-10.75, 5.10)	
Unplanned	-0.17	(-1.29, 0.96)		-0.06	(-3.89, 3.76)	
ICNARC Physiology Score (per point)	0.19	(0.13, 0.25)	<0.001	0.19	(0.02, 0.35)	0.026
ICU length of stay (per day)	0.02	(-0.03, 0.06)	0.44	-0.34	(-0.48, -0.20)	<0.001
Advanced respiratory support	3.62	(2.63, 4.61)	<0.001	1.96	(-1.84, 5.76)	0.31
Advanced cardiovascular support	2.06	(0.89, 3.22)	0.001	0.83	(-2.06, 3.72)	0.58
Renal support	1.52	(0.11, 2.93)	0.034	0.04	(-2.83, 2.91)	0.98
Neurological support	1.96	(0.39, 3.54)	0.014	2.95	(-0.42, 6.32)	0.086
Duration of adv. respiratory support (per day)	0.11	(0.05, 0.16)	<0.001	-0.16	(-0.32, 0.00)	0.051
Duration of adv. cardiovascular support (per day)	0.40	(0.15, 0.65)	0.002	0.11	(-0.33, 0.56)	0.62
Duration of renal support (per day)	0.16	(0.00, 0.32)	0.048	-0.15	(-0.43, 0.13)	0.28
Duration of neurological support (per day)	0.10	(-0.09, 0.29)	0.31	0.05	(-0.43, 0.53)	0.84
Death before acute hospital discharge	-0.49	(-1.52, 0.55)	0.36	N/A		

Coef., coefficient.

For peer review only

[a] Two family members returned questionnaires but did not complete any of the 24 FS-ICU items – responses were not imputed for these family members.

For peer review only

Table S5 Univariable analysis of factors associated with overall family satisfaction score by ICU outcome – ICU/hospital characteristics and contextual factors

Variables	Family members of ICU survivors [N=6,147[a]]			Family members of ICU non-survivors [N=870]		
	Coef.	95% CI	p-value	Coef.	95% CI	p-value
Hospital type (vs non-university)			0.51			0.62
University	0.06	(-3.63, 3.75)		-0.32	(-4.72, 4.07)	
University affiliated	1.93	(-1.56, 5.42)		1.68	(-2.29, 5.65)	
Number of ICU beds (per bed)	-0.05	(-0.23, 0.14)	0.63	0.02	(-0.22, 0.26)	0.85
Month of ICU admission (vs January)			0.95			0.85
February	-0.61	(-2.87, 1.65)		-0.03	(-6.90, 6.83)	
March	0.09	(-2.12, 2.30)		-0.06	(-6.73, 6.60)	
April	0.54	(-1.71, 2.79)		0.07	(-6.93, 7.07)	
May	-0.06	(-2.31, 2.18)		0.73	(-5.62, 7.08)	
June	-0.66	(-2.65, 1.34)		0.84	(-4.95, 6.64)	
July	0.85	(-1.41, 3.11)		3.91	(-2.71, 10.52)	
August	0.65	(-1.64, 2.93)		-0.70	(-6.87, 5.46)	
September	0.09	(-2.14, 2.31)		1.74	(-4.76, 8.25)	
October	0.44	(-1.76, 2.63)		1.15	(-5.69, 7.98)	
November	0.60	(-1.65, 2.85)		2.21	(-4.10, 8.53)	
December	0.69	(-1.57, 2.96)		5.16	(-1.13, 11.46)	
Questionnaire received while patient still in hospital	0.087	(-1.50, 1.67)	0.91	N/A		

Coef., coefficient.

[a] Two family members returned questionnaires but did not complete any of the 24 FS-ICU items – responses were not imputed for these family members.

For peer review only

Table S6 Sensitivity analyses –candidate determinants for the multivariable multilevel models for the family satisfaction in the intensive care unit

Candidate determinants	Justification inclusion/exclusion	Approach to modelling
**Family member level**		
Education level	It was not considered in the multivariable models due to higher than expected proportions of both "Not stated" (17%) and "Other" (21%) responses, suggesting a lack of comprehension of the categorisation used.	
Distance from home to hospital	No significant after adjusting for other variables in the model. It was dropped.	
Family member age, years	Controlling effect	Categorical (<30;30-39;40-49;50-59;60-69;70-79;80+)
Family member sex	Controlling effect	Categorical (male; female)
Family member ethnicity	Statistically significant in univariable	Categorical (white; non-white)
Next-of-kin/lives with patient	There was a strong multicollinearity between relationship to the patient and the other key variables of next-of-kin and lives with patient.	Categorical (lives with patient; Next-of-kin, does not live with patient; Not next-of-kin, does not live with patient)
Frequent visitor	Statistically significant in univariable	Binary (yes; no)
**Patient level**		
Patient ethnicity	It was not carried forward to the multivariable models due to collinearity with family member ethnicity.	
Patient age	Controlling effect	Continuous(linear)
Patient sex	Controlling effect	Categorical (male; female)
Dependency	Controlling effect	Categorical (none; minor or major; total)
Surgical status (vs non-surgical)	Controlling effect	Categorical (non-surgical; planned elective/scheduled; unplanned)
ICNARC Physiology Score	Statistically significant in univariable	Continuous(linear)
ICU length of stay (days)		Continuous(linear)
Organ support received in the ICU and duration (calendar days)	Once included in the multivariable model for	

For peer review only

of organ support among those receiving the support	survivors, only advanced respiratory support remained significant.	
Advanced respiratory support	It was found to be preferable to alternative variable of the duration of advanced respiratory support, which was correlated with ICU length of stay.	Binary (yes; no)
haematological malignancy	No significant after adjusting for other variables in the model. It was dropped.	
**ICU/hospital level**		
Hospital type	Controlling effect	Categorical (non-university; university; university affiliated)
Number of ICU beds	Controlling effect	Continuous(linear)

For peer review only

Table S7 Multivariable multilevel models for the satisfaction with care domain score

Variables	Family members of ICU survivors [N=6,143[a]]			Family members of ICU non-survivors [N=869[a]]		
	Coef.	95% CI	p-value	Coef.	95% CI	p-value
Fixed effects – family member level						
Constant	71.45	(66.67, 76.22)		55.29	(41.76, 68.82)	
Family member age, years (vs <30)			0.001			0.16
30-39	2.60	(0.81, 4.38)		2.50	(-1.97, 6.97)	
40-49	2.73	(1.16, 4.31)		4.31	(0.09, 8.54)	
50-59	2.91	(1.36, 4.44)		4.99	(0.93, 9.04)	
60-69	2.67	(1.08, 4.26)		4.89	(0.54, 9.23)	
70-79	2.66	(0.90, 4.41)		5.91	(0.88, 10.94)	
80+	-0.17	(-2.76, 2.41)		1.85	(-4.51, 8.21)	
Family member sex – female (vs male)	0.42	(-0.35, 1.20)	0.29	0.22	(-1.81, 2.25)	0.83
Family member ethnicity – white (vs non-white)	3.87	(1.77, 5.97)	<0.001	6.99	(0.19, 13.81)	0.044
Next-of-kin/lives with patient (vs lives with patient)			<0.001			0.15
Next-of-kin, does not live with patient	-1.14	(-2.26, -0.02)		0.95	(-2.39, 4.29)	
Not next-of-kin, does not live with patient	-2.44	(-3.32, -1.55)		-1.58	(-4.11, 0.94)	
Frequent visitor	2.49	(1.52, 3.46)	<0.001	1.49	(-1.27, 4.25)	0.29
Fixed effects – patient level						
Patient age (per 10 years)	0.03	(-0.25, 0.31)	0.83	1.21	(0.16, 2.26)	0.024
Patient sex – female (vs male)	0.06	(-0.85, 0.98)	0.87	1.85	(-0.79, 4.5)	0.17
Dependency (vs none)			0.006			0.68
Minor or major	-0.74	(-1.96, 0.46)		-0.94	(-3.98, 2.09)	
Total	-6.77	(-11.18, -2.36)		3.62	(-8.71, 15.95)	
Surgical status (vs non-surgical)			0.68			0.47
Planned elective/scheduled	-0.62	(-2.04, 0.78)		-4.85	(-12.71, 2.99)	
Unplanned	-0.15	(-1.27, 0.95)		-0.57	(-4.29, 3.13)	
ICNARC Physiology Score (per point)	0.14	(0.07, 0.21)	<0.001	0.14	(-0.03, 0.30)	0.10
ICU length of stay (per day)	-0.02	(-0.06, 0.02)	0.39	-0.30	(-0.45, -0.15)	<0.001
Advanced respiratory support	2.74	(1.66, 3.82)	<0.001			
Fixed effects – ICU/hospital level						
Hospital type (vs non-university)			0.51			0.58

For peer review only

University	0.94	(-3.58, 5.47)		-1.48	(-7.8 , 4.84)	
University affiliated	1.92	(-1.34, 5.19)		1.79	(-2.75, 6.34)	
Number of ICU beds (per bed)	-0.01	(-0.24, 0.23)	0.96	0.24	(-0.12, 0.59)	0.19
Random effects – SD (SE)						
Between ICUs	2.98	(0.60)		3.25	(1.11)	
Within ICUs between patients	9.76	(0.28)		10.47	(0.66)	
Within patients between family members	11.96	(0.19)		11.92	(0.42)	

Coef, coefficient; SE, standard error.

[a] Five patients were missing age group on both the questionnaire and web portal – due to the very small amount of missing data in this key variable, these missing values were not imputed.

For peer review only

Table S8 Multivariable multilevel models for the satisfaction with information domain score

**Variables**	**Family members of ICU survivors [N=6,143[a]]**			**Family members of ICU non-survivors [N=869[a]]**		
	Coef.	95% CI	p-value	Coef.	95% CI	p-value
Fixed effects – family member level						
Constant	66.07	(59.78, 72.21)		55.86	(39.34, 72.38)	
Family member age, years (vs <30)			0.63			0.28
30-39	0.28	(-2.22, 2.79)		1.23	(-4.92, 7.39)	
40-49	0.00	(-2.21, 2.21)		1.88	(-3.92, 7.68)	
50-59	0.55	(-1.62, 2.72)		2.88	(-2.70, 8.48)	
60-69	-0.1	(-2.35, 2.14)		4.24	(-1.71, 10.2)	
70-79	-0.41	(-2.89, 2.08)		6.43	(-0.45, 13.31)	
80+	-2.67	(-6.35, 1.01)		-1.96	(-10.71, 6.79)	
Family member sex – female (vs male)	0.20	(-0.89, 1.30)	0.72	1.01	(-1.81, 3.82)	0.49
Family member ethnicity – white (vs non-white)	4.73	(1.78, 7.68)	0.002	9.34	(0.47, 18.21)	0.039
Next-of-kin/lives with patient (vs lives with patient)			<0.001			0.38
Next-of-kin, does not live with patient	-2.39	(-3.97, 0.81)		1.43	(-3.09, 5.95)	
Not next-of-kin, does not live with patient	-2.57	(-3.83, 1.31)		-1.21	(-4.69, 2.28)	
Frequent visitor	2.11	(0.74, 3.48)	0.002	0.44	(-3.33, 4.22)	0.82
Fixed effects – patient level						
Patient age (per 10 years)	-0.22	(-0.61, 0.18)	0.28	0.92	(-0.43, 2.27)	0.18
Patient sex – female (vs male)	0.32	(-0.98, 1.62)	0.63	1.93	(-1.48, 5.35)	0.27
Dependency (vs none)			0.61			0.51
Minor or major	-0.49	(-2.2, 1.2)		-0.28	(-4.11, 3.53)	
Total	-2.69	(-8.92, 3.52)		9.15	(-6.57, 24.87)	
Surgical status (vs non-surgical)			0.88			0.84
Planned elective/scheduled	-0.32	(-2.32, 1.66)		-0.88	(-10.97, 9.21)	
Unplanned	0.23	(-1.33, 1.80)		-1.4	(-6.16, 3.36)	
ICNARC Physiology Score (per point)	0.23	(0.13, 0.33)	<0.001	0.15	(-0.04, 0.36)	0.13
ICU length of stay (per day)	-0.05	(-0.11, 0.01)	0.14	-0.43	(-0.62, -0.24)	<0.001
Advanced respiratory support	3.34	(1.83, 4.85)	<0.001	--		
Fixed effects – ICU/hospital level						
Hospital type (vs non-university)			0.45			0.58

For peer review only

University	1.69	(-3.71, 7.08)		0.35	(-6.42, 7.13)	
University affiliated	2.48	(-1.42, 6.40)		2.53	(-2.32, 7.39)	
Number of ICU beds (per bed)	-0.03	(-0.31, 0.24)	0.81	0.21	(-0.17, 0.61)	0.27
Random effects – SD (SE)						
Between ICUs	3.48	(0.73)		2.81	(1.37)	
Within ICUs between patients	13.64	(0.41)		12.38	(0.97)	
Within patients between family members	16.88	(0.27)		17.02	(0.60)	

Coef, coefficient; SE, standard error.

[a] Five patients were missing age group on both the questionnaire and web portal – due to the very small amount of missing data in this key variable, these missing values were not imputed.

For peer review only

Table S9 Multivariable multilevel models for the satisfaction with the decision-making process domain score

Variables	Family members of ICU survivors [N=6,143[a]]			Family members of ICU non-survivors [N=869[a]]		
	Coef.	95% CI	p-value	Coef.	95% CI	p-value
Fixed effects – family member level						
Constant	61.65	(55.17, 68.14)		39.62	(20.14, 59.09)	
Family member age, years (vs <30)			0.061			0.40
30-39	1.66	(-1.63, 4.95)		1.37	(-5.35, 8.10)	
40-49	0.02	(-2.76, 2.82)		2.73	(-3.47, 8.95)	
50-59	0.52	(-2.21, 3.25)		3.34	(-2.61, 9.31)	
60-69	-1.43	(-4.48, 1.61)		3.35	(-3.05, 9.77)	
70-79	-1.09	(-4.32, 2.13)		6.25	(-1.36, 13.88)	
80+	-3.87	(-8.43, 0.69)		-3.13	(-12.88, 6.61)	
Family member sex – female (vs male)	-0.18	(-1.42, 1.04)	0.77	1.66	(-1.37, 4.71)	0.28
Family member ethnicity – white (vs non-white)	0.81	(-2.67, 4.30)	0.65	6.46	(-4.24, 17.15)	0.24
Next-of-kin/lives with patient (vs lives with patient)			0.10			0.86
Next-of-kin, does not live with patient	-0.93	(-2.93, 1.05)		1.39	(-3.49, 6.28)	
Not next-of-kin, does not live with patient	-1.65	(-3.22, 0.07)		0.48	(-3.49 , 4.46)	
Frequent visitor	5.31	(3.38, 7.23)	<0.001	3.84	(-0.21, 7.91)	0.063
Fixed effects – patient level						
Patient age (per 10 years)	0.26	(-0.20, 0.73)	0.27	2.19	(0.61, 3.78)	0.007
Patient sex – female (vs male)	0.79	(-0.84, 2.43)	0.34	1.29	(-2.67, 5.26)	0.52
Dependency (vs none)			0.44			0.47
Minor or major	1.34	(-0.74, 3.43)		2.91	(-1.48, 7.29)	
Total	0.11	(-7.42, 7.64)		4.27	(-17.36, 25.91)	
Surgical status (vs non-surgical)			0.25			0.68
Planned elective/scheduled	-1.83	(-4.35, 0.68)		-1.09	(-12.59, 10.41)	
Unplanned	-1.35	(-3.41, 0.71)		2.35	(-3.20, 7.91)	
ICNARC Physiology Score (per point)	0.12	(0.01, 0.24)	0.040	0.19	(-0.04, 0.44)	0.12
ICU length of stay (per day)	0.03	(-0.04, 0.11)	0.39	-0.17	(-0.39, 0.03)	0.11
Advanced respiratory support	3.03	(1.08, 4.97)	0.002	--		
Fixed effects – ICU/hospital level						

For peer review only

Hospital type (vs non-university)			0.50			0.55
University	-0.41	(-4.27, 3.46)		-4.44	(-12.41, 3.53)	
University affiliated	1.51	(-1.37, 4.39)		-0.86	(-6.56, 4.83)	
Number of ICU beds (per bed)	0.02	(-0.19, 0.23)	0.85	0.47	(0.02, 0.93)	0.042
Random effects – SD (SE)						
Between ICUs	2.06	(0.66)		3.33	(1.50)	
Within ICUs between patients	17.24	(0.50)		15.84	(1.06)	
Within patients between family members	17.02	(0.40)		16.81	(0.66)	

Coef, coefficient; SE, standard error.

[a] Five patients were missing age group on both the questionnaire and web portal – due to the very small amount of missing data in this key variable, these missing values were not imputed.

For peer review only

Figure S2 Variation across ICUs in the mean: satisfaction with care domain score (A) before and (B) after adjustment; satisfaction with information domain score (C) before and (D) after adjustment; and satisfaction with the decision-making process domain score (E) before and (F) after adjustment

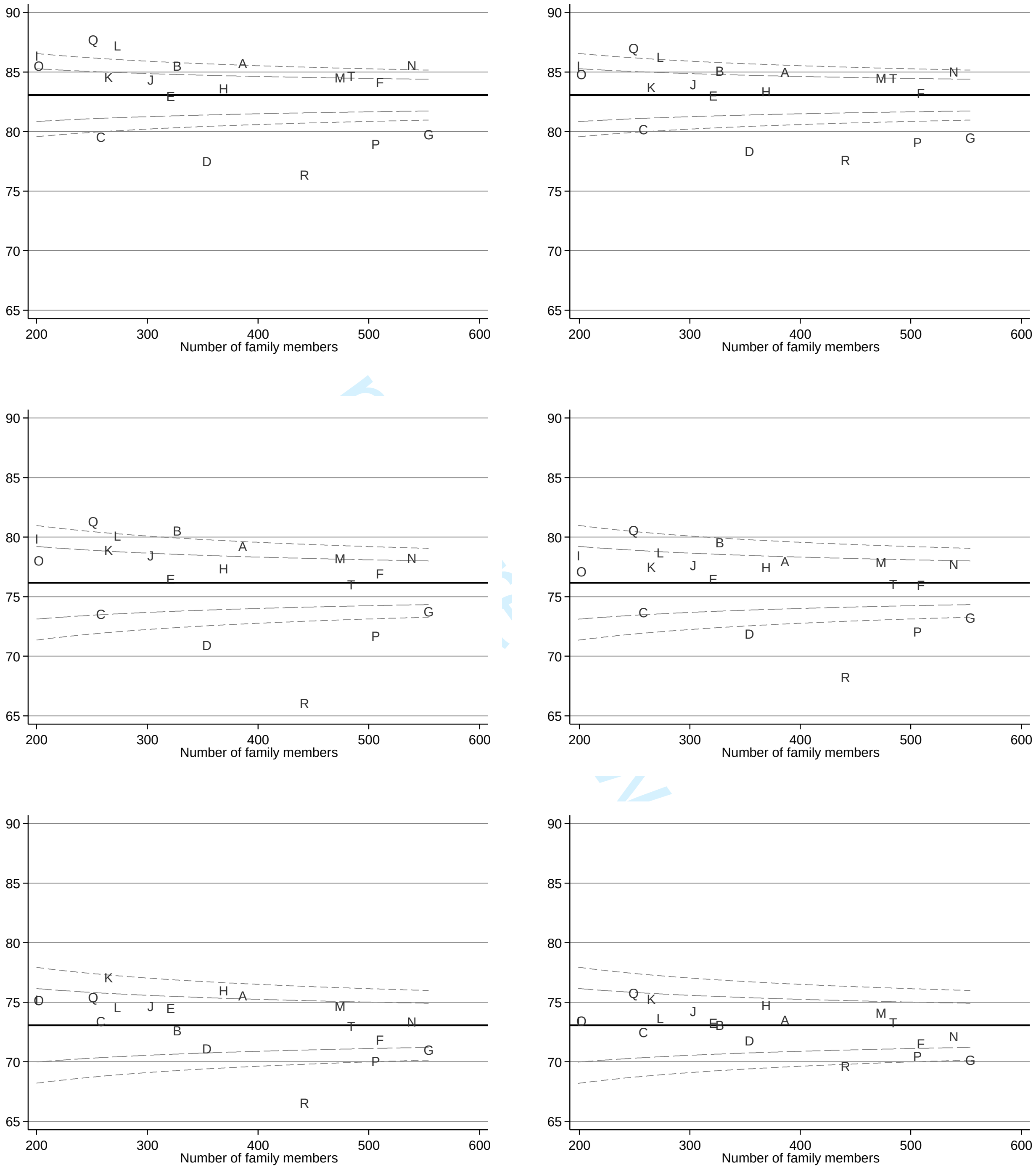

Table S10 Sensitivity analyses – alternative approach to handling missing data (family members of ICU survivors)

**Variables**	**Complete case [N=2,351]**			**Traditional approach [N=5,756]**		
	Coef.	SE	p-value	Coef.	SE	p-value
Constant	72.60	3.18		70.35	2.49	
Family member age, years (vs <30)			0.61			0.20
30-39	0.13	1.40		1.47	0.97	
40-49	0.85	1.22		1.41	0.86	
50-59	0.66	1.20		1.58	0.84	
60-69	0.65	1.30		1.47	0.88	
70-79	0.77	1.47		1.69	0.98	
80+	-3.06	2.26		-1.22	1.50	
Family member sex – female (vs male)	0.94	0.60	0.12	0.21	0.43	0.63
Family member ethnicity – white (vs non-white)	7.58	1.58	<0.001	3.99	1.16	0.001
Next-of-kin/lives with patient (vs lives with patient)			0.071			0.002
Next-of-kin, does not live with patient	-1.69	0.85		-1.36	0.61	
Not next-of-kin, does not live with patient	-1.42	0.72		-1.70	0.50	
Frequent visitor	1.18	0.82	0.15	2.21	0.55	<0.001
Patient age (per 10 years)	-0.09	0.22	0.67	-0.07	0.15	0.64
Patient sex – female (vs male)	-1.20	0.73	0.10	0.13	0.52	0.79
Dependency (vs none)			0.70			0.45
Minor or major	-0.44	0.92		-0.19	0.68	
Total	-2.19	2.98		-3.14	2.51	
Surgical status (vs non-surgical)			0.056			0.47
Planned elective/scheduled	-3.11	1.30		-0.93	0.80	
Unplanned	-0.44	0.88		0.02	0.62	
ICNARC Physiology Score (per point)	0.08	0.05	0.14	0.15	0.04	<0.001
ICU length of stay (per day)	-0.04	0.03	0.28	-0.04	0.03	0.17
Advanced respiratory support	1.39	0.87	0.11	2.40	0.60	<0.001
Hospital type (vs non-university)			0.42			0.34
University	0.56	2.36		1.45	2.22	

For peer review only

University affiliated	2.24	1.72		2.34	1.61	
Number of ICU beds (per bed)	0.07	0.12	0.59	-0.02	0.11	0.83

Coef., coefficient; SE, standard error.

For peer review only

Table S11 Sensitivity analyses – alternative approaches to handling missing data (family members of ICU non-survivors)

Variables	Complete case [N=547]			Traditional approach [N=851]		
	Coef.	SE	p-value	Coef.	SE	p-value
Constant	54.46	7.72		56.28	6.80	
Family member age, years (vs <30)			0.17			0.086
30-39	4.38	3.01		3.14	2.44	
40-49	7.51	2.75		4.87	2.31	
50-59	6.19	2.62		4.50	2.22	
60-69	7.41	2.85		5.94	2.37	
70-79	6.99	3.69		7.07	2.82	
80+	7.52	4.41		0.32	3.61	
Family member sex – female (vs male)	-0.02	1.43	0.99	0.40	1.11	0.72
Family member ethnicity – white (vs non-white)	9.64	4.21	0.022	7.47	3.58	0.037
Next-of-kin/lives with patient (vs lives with patient)			0.97			0.38
Next-of-kin, does not live with patient	0.13	2.20		1.27	1.82	
Not next-of-kin, does not live with patient	-0.32	1.81		-0.82	1.40	
Frequent visitor	1.32	1.96	0.50	0.99	1.51	0.51
Patient age (per 10 years)	0.69	0.66	0.29	1.09	0.55	0.048
Patient sex – female (vs male)	1.56	1.69	0.36	2.02	1.41	0.15
Dependency (vs none)			0.47			0.66
Minor or major	-0.61	1.86		-0.32	1.58	
Total	8.53	7.42		5.59	6.45	
Surgical status (vs non-surgical)			0.84			0.51
Planned elective/scheduled	-0.33	5.61		-4.86	4.22	
Unplanned	-1.38	2.33		-0.44	1.95	
ICNARC Physiology Score (per point)	0.24	0.10	0.022	0.18	0.09	0.041
ICU length of stay (per day)	-0.27	0.09	0.003	-0.33	0.08	<0.001
Hospital type (vs non-university)			0.83			0.77
University	-1.15	3.20		-0.11	3.01	
University affiliated	0.84	2.29		1.49	2.17	

For peer review only

Number of ICU beds (per bed)	0.25	0.19	0.17	0.21	0.17	0.23

Coef., coefficient; SE, standard error.

For peer review only

STROBE Statement—checklist of items that should be included in reports of observational studies

	Item No	Recommendation	Page No
**Title and abstract**	1	(*a*) Indicate the study's design with a commonly used term in the title or the abstract	1
		(*b*) Provide in the abstract an informative and balanced summary of what was done and what was found	2
**Introduction**			
Background/rationale	2	Explain the scientific background and rationale for the investigation being reported	4
Objectives	3	State specific objectives, including any prespecified hypotheses	4
**Methods**			
Study design	4	Present key elements of study design early in the paper	4
Setting	5	Describe the setting, locations, and relevant dates, including periods of recruitment, exposure, follow-up, and data collection	4
Participants	6	(*a*) *Cohort study*—Give the eligibility criteria, and the sources and methods of selection of participants. Describe methods of follow-up<br>*Case-control study*—Give the eligibility criteria, and the sources and methods of case ascertainment and control selection. Give the rationale for the choice of cases and controls<br>*Cross-sectional study*—Give the eligibility criteria, and the sources and methods of selection of participants	4-5
		(*b*) *Cohort study*—For matched studies, give matching criteria and number of exposed and unexposed<br>*Case-control study*—For matched studies, give matching criteria and the number of controls per case	N/A
Variables	7	Clearly define all outcomes, exposures, predictors, potential confounders, and effect modifiers. Give diagnostic criteria, if applicable	4-6
Data sources/ measurement	8*	For each variable of interest, give sources of data and details of methods of assessment (measurement). Describe comparability of assessment methods if there is more than one group	
Bias	9	Describe any efforts to address potential sources of bias	4
Study size	10	Explain how the study size was arrived at	N/A
Quantitative variables	11	Explain how quantitative variables were handled in the analyses. If applicable, describe which groupings were chosen and why	5-6
Statistical methods	12	(*a*) Describe all statistical methods, including those used to control for confounding	5-6
		(*b*) Describe any methods used to examine subgroups and interactions	N/A
		(*c*) Explain how missing data were addressed	5-6
		(*d*) *Cohort study*—If applicable, explain how loss to follow-up was addressed<br>*Case-control study*—If applicable, explain how matching of cases and controls was addressed<br>*Cross-sectional study*—If applicable, describe analytical methods taking account of sampling strategy	
		(*e*) Describe any sensitivity analyses	6

Continued on next page

For peer review only - http://bmjopen.bmj.com/site/about/guidelines.xhtml

Results

Participants	13*	(a) Report numbers of individuals at each stage of study—eg numbers potentially eligible, examined for eligibility, confirmed eligible, included in the study, completing follow-up, and analysed	6
		(b) Give reasons for non-participation at each stage	
		(c) Consider use of a flow diagram	Supplementary materials Figure S1
Descriptive data	14*	(a) Give characteristics of study participants (eg demographic, clinical, social) and information on exposures and potential confounders	Page 6-7 & Tables 1 & 2
		(b) Indicate number of participants with missing data for each variable of interest	Supplementary materials Tables S10 & S11
		(c) *Cohort study*—Summarise follow-up time (eg, average and total amount)	N/A
Outcome data	15*	*Cohort study*—Report numbers of outcome events or summary measures over time	7 & Table 3
		Case-control study—Report numbers in each exposure category, or summary measures of exposure	
		Cross-sectional study—Report numbers of outcome events or summary measures	
Main results	16	(*a*) Give unadjusted estimates and, if applicable, confounder-adjusted estimates and their precision (eg, 95% confidence interval). Make clear which confounders were adjusted for and why they were included	7 & 8, Table 4 & Supplement Tables S7-9 &
		(*b*) Report category boundaries when continuous variables were categorized	
		(*c*) If relevant, consider translating estimates of relative risk into absolute risk for a meaningful time period	
Other analyses	17	Report other analyses done—eg analyses of subgroups and interactions, and sensitivity analyses	10-13 & supplement

Discussion

Key results	18	Summarise key results with reference to study objectives	13-14
Limitations	19	Discuss limitations of the study, taking into account sources of potential bias or imprecision. Discuss both direction and magnitude of any potential bias	13-14
Interpretation	20	Give a cautious overall interpretation of results considering objectives, limitations, multiplicity of analyses, results from similar studies, and other relevant evidence	13-14
Generalisability	21	Discuss the generalisability (external validity) of the study results	13-14

Other information

Funding	22	Give the source of funding and the role of the funders for the present study and, if applicable, for the original study on which the present article is based	15

*Give information separately for cases and controls in case-control studies and, if applicable, for exposed and unexposed groups in cohort and cross-sectional studies.

Note: An Explanation and Elaboration article discusses each checklist item and gives methodological background and published examples of transparent reporting. The STROBE checklist is best used in conjunction with this article (freely

For peer review only - http://bmjopen.bmj.com/site/about/guidelines.xhtml
For peer review only

available on the Web sites of PLoS Medicine at http://www.plosmedicine.org/, Annals of Internal Medicine at http://www.annals.org/, and Epidemiology at http://www.epidem.com/). Information on the STROBE Initiative is available at www.strobe-statement.org.

For peer review only

For peer review only - http://bmjopen.bmj.com/site/about/guidelines.xhtml

BMJ Open

Family satisfaction with critical care in the United Kingdom: a multi-centre cohort study

Journal:	*BMJ Open*
Manuscript ID	bmjopen-2019-028956.R2
Article Type:	Research
Date Submitted by the Author:	05-Jul-2019
Complete List of Authors:	Ferrando, Paloma; Intensive Care National Audit and Research Centre,<br>Gould, Doug; Intensive Care National Audit and Research Centre<br>Walmsley, Emma; Intensive Care National Audit and Research Centre<br>Richards-Belle, Alvin; Intensive Care National Audit and Research Centre<br>Canter, Ruth; Intensive Care National Audit and Research Centre<br>Saunders, Steven; Intensive Care National Audit and Research Centre<br>Harrison, David; Intensive Care National Audit and Research Centre<br>Harvey, Sheila; London School of Hygiene & Tropical Medicine, Global Health and Development<br>Heyland, Daren; Kingston General Hospital, Clinical Evaluation Research Unit; Queens University, Department of Critical Care Medicine<br>Hinton, Lisa; University of Oxford, Health Experiences Research Group, Nuffield Department of Primary Care Health Sciencs<br>McColl, Elaine; Newcastle University Institute for Health and Society<br>Richardson, Annette; Freeman Hospital, Perioperative and Critical Care<br>Richardson, Michael<br>Wright, Stephen; Freeman Hospital, Perioperative and Critical Care<br>Rowan, Kathryn; Intensive Care National Audit and Research Centre
**Primary Subject Heading**:	Intensive care
Secondary Subject Heading:	Patient-centred medicine
Keywords:	Adult intensive & critical care < ANAESTHETICS, Quality in health care < HEALTH SERVICES ADMINISTRATION & MANAGEMENT, Family satisfaction

SCHOLARONE™
Manuscripts

Family satisfaction with critical care in the United Kingdom: a multi-centre cohort study

Authors

Paloma Ferrando-Vivas, *statistician*[1], Doug W Gould, *senior researcher*[1], Emma Walmsley, *study co-ordinator*[1], Alvin Richards-Belle, *trial manager*[1], Ruth R Canter, *research assistant*[1], Steven Saunders, *research administrator*[1], David A Harrison, *head statistician*[1], Sheila E Harvey, *associate professor*[2], Daren K Heyland, *director of the Kingston General Hospital Clinical Evaluation Research Unit and professor of medicine and epidemiology*[3], Lisa Hinton, *senior qualitative researcher*[4], Elaine McColl, *professor of health services research*[5], Annette Richardson, *nurse consultant in critical care*[6], Michael Richardson, *patient public representative*[7], Stephen E Wright, *consultant in anaesthesia and intensive care*[6], Kathryn M Rowan, *director of scientific & strategic development*[1].

[1]Intensive Care National Audit & Research Centre (ICNARC), London, United Kingdom, WC1V 6AZ

[2]London School of Hygiene & Tropical Medicine, Global Health and Development, London, United Kingdom

[3]Kingston General Hospital, Clinical Evaluation Research Unit, Kingston, Canada and Queen's University, Department of Critical Care Medicine, School of Medicine, Canada.

[4] University of Oxford, Health Experiences Research Group, Nuffield Department of Primary Care Health Sciences, Oxford, United Kingdom.

Institute of Health and Society, Newcastle University, Newcastle upon Tyne, United Kingdom.

[6]Freeman Hospital, Perioperative and Critical Care, Newcastle Upon Tyne Hospitals NHS Foundation Trust, Newcastle upon Tyne, United Kingdom.

[7]Newcastle Upon Tyne, United Kingdom.

Corresponding author:

Kathryn M. Rowan (ORCid iD: 0000 0001 8217 5602), Director of Scientific & Strategic Development, Intensive Care National Audit & Research Centre, Napier House, 24 High Holborn, London WC1V 6AZ

Tel: 020 7831 6878

Fax: 020 7831 6879

e-mail: Kathy.rowan@icnarc.org

For peer review only

For peer review only - http://bmjopen.bmj.com/site/about/guidelines.xhtml

Abstract

Objective: To assess family satisfaction with intensive care units (ICUs) in the United Kingdom using the Family Satisfaction in the Intensive Care Unit 24-item questionnaire (FS-ICU-24), and to investigate how characteristics of patients and their family members impact on family satisfaction.

Design: Prospective cohort study nested within a national clinical audit database.

Setting: Stratified, random sample of 20 adult general ICUs participating in the Intensive Care Audit & Research Centre (ICNARC) Case Mix Programme.

Participants: Family members of patients staying at least 24 hours in ICU were recruited between May 2013 and June 2014.

Interventions: Consenting family members were sent a postal questionnaire three weeks after the patient died or was discharged from ICU. Up to four family members were recruited per patient.

Main outcome measures: Family satisfaction measured using the FS-ICU-24 questionnaire.

Main Results: 12,346 family members of 6,380 patients were recruited and 7,173 (58%) family members of 4,615 patients returned a completed questionnaire. Overall and domain specific family satisfaction scores were high (mean overall family satisfaction 80, satisfaction with care 83, satisfaction with information 76, and satisfaction with decision-making 73 out of 100) but varied significantly across adult general ICUs studied and by whether the patient survived ICU. For family members of ICU survivors, characteristics of both family member (age, ethnicity, relationship to patient (next-of-kin and/or lived with patient) and visit frequency) and the patient (acute severity of illness and receipt of invasive mechanical ventilation) were significant determinants of family satisfaction, whereas, for family members of ICU non-survivors, only patient characteristics (age, acute severity of illness, and duration of stay) were significant.

Conclusions: Overall family satisfaction in UK adult general ICUs was high but varied significantly. Adjustment for differences in family member/patient characteristics is important to avoid falsely identifying ICUs as statistical outliers.

Study registration: ISRCTN 47363549

For peer review only

Keywords: critical care; intensive care units; personal satisfaction; family; quality of care; communication

Strengths and limitations of this study

- This is the largest study assessing family satisfaction with ICU care.
- Unbiased selection and stratification of participating units ensured geographical spread (north, south, east, and west England, Wales and Northern Ireland), hospital type (university or non-university) and ICUs of different sizes (large or small – based on number of beds) that recruited for one year to avoid bias from seasonal variation.
- Nesting our study within the Case Mix Programme national clinical audit was efficient and allowed for linkage of family members' to patient data.
- The same mode and timing of delivery of the FS-ICU-24 was employed for family members of ICU survivors and non-survivors, avoiding potential sampling bias and allowing for meaningful comparisons between these groups.
- Despite our very large sample size, we achieved a modest response rate (58%), which was in line with previous published studies.

For peer review only - http://bmjopen.bmj.com/site/about/guidelines.xhtml

Introduction

Humanity of health care, often measured as patient experience, is increasingly seen as one of the three pillars of quality, alongside effectiveness and equity. Eliciting the views and experiences of patients is now seen as essential in delivering a high quality service (1). However, given that approximately 20% of patients admitted to intensive care units (ICUs) die and survivors are often unable to recall their experiences, measuring patient experience in ICU has particular challenges. For this reason, measures of family experience have been developed to help understand the humanity of ICU care.

The most widely validated measure of family experience is the Family Satisfaction in the Intensive Care Unit questionnaire (FS-ICU)(2). This describes satisfaction, overall and in two domains – *satisfaction with care* and *satisfaction with decision making* (3-5). Family satisfaction reflects the extent to which perceived needs and expectations of family members are met by health-care professionals, and may be influenced by a number of factors including families' expectations, information and communication, family-related factors (such as attitudes towards life and death, social, cultural and religious backgrounds, etc.), patient-related factors (such as illness severity and whether the patient survives the ICU), hospital infrastructure, and process of care.(4, 6, 7)

This paper reports the results of a large, prospective, multicentre, cohort study describing family satisfaction with ICU care in the UK. The overall aim of the Family-Reported Experiences Evaluation (FREE) study was to inform the potential routine use of the FS-ICU-24 questionnaire for quality improvement in adult general ICUs in the UK. Specific aims were to investigate how characteristics of patients and their family members impact on family satisfaction, and to explore how family satisfaction varies across ICUs, before and after adjustment for family member and patient characteristics identified as being associated with family satisfaction.

For peer review only

Methods

This large, prospective, multicentre cohort study was nested in the Intensive Care National Audit & Research Centre (ICNARC) Case Mix Programme (CMP) – the national clinical audit of adult general ICUs in England, Wales and Northern Ireland. A stratified sample of 20 ICUs were selected to ensure geographical spread (north, south, east, and west England, Wales and Northern Ireland), hospital type (university or non-university) and ICUs of different sizes (large or small – based on number of beds) and recruited for one year to avoid bias from seasonal variation. In accordance with care standards for UK ICUs at the time of data collection, nurse/patient ratios were 1:1 and 1:2 for Level 3 (Intensive

For peer review only - http://bmjopen.bmj.com/site/about/guidelines.xhtml

Care) and Level 2 (High Dependency) patients, respectively. The study was reviewed and approved by the National Research Ethics Service Committee South Central - Berkshire B (reference 13/SC/0037) and was registered prospectively (ISRCTN47363549).

Patient and Public Involvement

Engagement with patient and their family members was vital to ensuring the successful delivery of the FREE study. A former ICU patient and a family member of a former ICU patient were co-investigators on the FREE study and contributed to all aspects of the study including: design; conduct; management; analysis; interpretation of results; and dissemination as members of the study management group. Additionally, the study steering committee included patient and family members.

Recruitment and follow-up

Recruitment and follow-up of family members have been described in detail elsewhere (8). Briefly, a 'family member' was defined as any person with close familial, social or emotional relationship to the patient and was not restricted solely to next-of-kin. Up to four family members of patients who spent >24 hours in ICU were eligible to participate if they met the following criteria: aged ≥18 years; had physically visited the patient's bedside at least once after the first 24 hours; had a UK postal address; and had not already been recruited into the study.

Patients were followed-up to ICU discharge. Approximately three weeks after the patient had either been discharged from or died in the ICU, a questionnaire pack was mailed to their recruited and consented family member(s) direct from the ICNARC Clinical Trials Unit. Data from completed questionnaires were entered centrally onto a secure database. All identifiable information such as names (e.g. of patients, family members, and ICU staff members) were removed. Quality checking of entered data was conducted and, for a 20% random sample, accuracy was verified. All fields in the database with missing data were verified against the paper questionnaires.

Statistical analysis

Item responses were rescaled and, where relevant, reversed, according to the developer's rules, so that each response was on a scale from 0 (least satisfied) to 100 (most satisfied) (5). Recent work from our group (9) established the construct validity of the FS-ICU 24-item questionnaire (FS-ICU-24) was improved by using three domains (splitting the *satisfaction with decision making* domain into two – *satisfaction with information* and *satisfaction with decision making process*). Overall family satisfaction score and three domain scores were calculated by averaging the item responses for the relevant items.

For peer review only - http://bmjopen.bmj.com/site/about/guidelines.xhtml

Family member and patient characteristics were described by mean and standard deviation (SD), median and quartiles, or number and percentage stratified by the patient outcome (alive/dead). Variation in family satisfaction was analysed across the following factors: patient; family member; ICU/hospital (hospital teaching status and number of beds in the ICU); and other contextual.

These factors were then explored using univariable and multivariable multilevel linear regression models (10) with a primary outcome of the overall family satisfaction score. Family member level and patient level variables that were statistically significant in the univariable models along with a priori key family member/patient variables (age, sex), were carried forward to the multivariable multilevel modelling process. (8) To reflect likely differences in the associations between factors and outcomes, separate models were fitted for family members of ICU survivors and non-survivors.

After modelling, the normality of error assumption was assessed by measurements of skewness. Normal probability plots were also used to assess the distribution of residuals at each level. As a sensitivity analysis we ran a multilevel regression model on the square root of the score using the same set of variables to confirm inference. In secondary analyses, separate models were fitted for the three individual domains of family satisfaction. All analyses were conducted in Stata/SE Version 13.0 (StataCorp, College Station, TX).

Variation in family satisfaction across ICUs was assessed graphically using funnel plots, which plot the average family satisfaction score for each critical care unit against the number of family members returning questionnaires. Control limits placed at 2 and 3 SDs around the overall mean indicate the regions of the funnel within which we would expect 95% and 99.8% of points to lie if all variation was due to chance (11).

Due to the natural structure of the data and the planned analysis multilevel multiple imputation (MLMI) was used to complete non- and partial responses for outcomes and family member characteristics. Data were imputed using REALCOM-Impute, an MLwiN 2.15 macro that generates imputations for hierarchical data (12). To test whether our findings were influenced by using imputed data, we also conducted sensitivity analyses using a traditional approach to scoring the FS-ICU-24 by including only responders with ≥60% of items completed. All analyses were conducted in accordance with a pre-defined statistical analysis plan and reported in line with the Strengthening the Reporting of Observational Studies in Epidemiology (STROBE) guidance on the analysis of observational studies.(13)

For peer review only - http://bmjopen.bmj.com/site/about/guidelines.xhtml

Results

Of the 210 adult, general ICUs participating in the CMP, 142 (67.6%) expressed an interest in participating and the 20 ICUs were selected using stratified, random sampling. The characteristics and outcomes of all admissions to the study ICUs were similar to admissions to all ICUs in the CMP during the same period (Supplementary Table S1).

Between 28 May 2013 and 30 June 2014, 18,757 patients were admitted to the 20 ICUs, of whom 12,730 patients stayed at least 24 hours in the ICU. From these, 12,346 family members of 6380 patients were recruited. Fully or partially completed questionnaires were returned by 7173 family members of 4615 patients. Family members of patients for whom no CMP data were available were not included, so finally, 7019 were included in the final analysis (Supplementary Figure S1).

Response rates varied by family member characteristics, including; age, gender, ethnicity, level of deprivation (based on residential postcode), level of education, and relationship with the patient. Family members documented in ICU records as next-of-kin were more likely to complete the questionnaire than those who were not, whilst family members for whom English was their first language were more likely to complete the questionnaire than those for whom it was not (Table S2).

A detailed description of the inclusion process, response rates and responders' characteristics has been reported in Family Reported Experiences Evaluation (FREE) study report (8). Comparisons of family member and patient characteristics for ICU survivors and non-survivors are presented in Table 1 and Table 2, respectively.

Table 1 Family member characteristics stratified by the patient's ICU outcome

Family member characteristics	All Family members [N=7,019]	Family members of ICU survivors[N=6,149]	Family members of ICU non-survivors [N=870]
Age, mean (SD)	54 (15.1)	54 (15.0)	52 (15.2)
Age group, n (%)			
<30	507 (7.5)	439 (7.4)	68 (8.0)
30-39	701 (10.3)	595 (10.0)	106 (12.5)
40-49	1,423 (21.0)	1,245 (21.0)	178 (21.0)
50-59	1,614 (23.8)	1,406 (23.7)	208 (24.6)
60-69	1,507 (22.2)	1,334 (22.5)	173 (20.4)
70-79	827 (12.2)	747 (12.6)	80 (9.5)
80+	204 (3.0)	171 (2.9)	33 (3.9)
Sex, n (%)			
Male	2,327 (33.5)	2,052 (33.7)	275 (31.9)
Female	4,622 (66.5)	4,034 (66.3)	588 (68.1)
Ethnicity, n (%)			

For peer review only - http://bmjopen.bmj.com/site/about/guidelines.xhtml

White	6,555 (94.0)	5,738 (93.9)	817 (94.6)
Asian	138 (2.0)	114 (1.9)	24 (2.8)
Black	54 (0.8)	50 (0.8)	4 (0.5)
Mixed ethnicity or other ethnic group	88 (1.3)	84 (1.4)	4 (0.5)
Not stated	139 (2.0)	124 (2.0)	15 (1.7)
Relationship to patient, n (%) ("I am the patient's…")			
Partner	2,096 (29.9)	1,891 (30.8)	205 (23.6)
Child	654 (9.3)	1,893 (30.8)	346 (39.8)
Parent	2,239 (31.9)	622 (10.1)	32 (3.7)
Sibling	704 (10.0)	624 (10.1)	80 (9.2)
Other relative	969 (13.8)	799 (13.0)	170 (19.5)
Other non-relative	356 (5.1)	319 (5.2)	37 (4.3)
Next-of-kin, n (%)	3,520 (50.2)	3,153 (51.4)	367 (42.3)
Lives with patient, n (%)	2,559 (36.5)	2,311 (37.6)	248 (28.5)
Highest level of education, n (%)			
NVQ level 1 or 2	1,683 (28.9)	1,465 (28.9)	218 (29.1)
NVQ level 3	1,123 (19.3)	989 (19.5)	134 (17.9)
NVQ level 4 or 5	1,769 (30.4)	1,537 (30.3)	232 (31.0)
Other	1,244 (21.4)	1,080 (21.3)	164 (21.9)
Quintile of deprivation, n (%)			
1 (least deprived)	1,190 (17.1)	1,164 (19.9)	159 (19.4)
2	1,405 (20.2)	1,281 (21.9)	181 (22.1)
3	1,488 (21.4)	1,238 (21.1)	181 (22.1)
4	1,488 (21.4)	1,189 (20.3)	169 (20.7)
5 (most deprived)	1,391 (20.0)	989 (16.9)	128 (15.6)
Distance (km) from home to hospital, median (IQR)	12.4 (5.4 33.6) [6,714]	12 (6, 34)	12 (5, 33)
Previous experience of ICU as a family member, n (%)	1,841 (26.6)	1,641 (27.1)	200 (23.3)
Frequent visitor, n (%)	5,403 (78.9)	4,713 (78.6)	690 (81.2)

NVQ, National Vocational Qualification level 1 or 2, equivalent to GCSE or O-level (school exams taken at age 16); NVQ level 3, equivalent to A-level, AS-level or High School Certificate (school exams taken at age 18); NVQ level 4 or 5, equivalent to degree, Higher degree, Higher National Certificate, Higher National Diploma.

Table 2 Patient characteristics stratified by ICU outcome

**Patient characteristics**	**All patients [N=4,506]**	**ICU survivors [N=4,007]**	**ICU non-survivors [N=499]**
Age, mean (SD)	63 (17.0)	63 (17.3)	68 (13.2)
Age group, n (%)			
<30	254 (5.6)	246 (6.1)	8 (1.6)
30-39	232 (5.1)	223 (5.6)	9 (1.8)
40-49	412 (9.1)	384 (9.6)	28 (5.6)
50-59	643 (14.3)	586 (14.6)	57 (11.4)
60-69	1,100 (24.4)	966 (24.1)	134 (26.9)
70-79	1,159 (25.7)	1,003 (25.0)	156 (31.3)

For peer review only

For peer review only - http://bmjopen.bmj.com/site/about/guidelines.xhtml

80+	706 (15.7)	599 (14.9)	107 (21.4)
Sex, n (%)			
Male	2,561 (56.8)	2,264 (56.5)	297 (59.5)
Female	1,945 (43.2)	1,743 (43.5)	202 (40.5)
Ethnicity, n (%)			
White	4,176 (92.7)	3,706 (92.5)	470 (94.2)
Asian or Asian British	81 (1.8)	69 (1.7)	12 (2.4)
Black or black British	42 (0.9)	39 (1.0)	3 (0.6)
Mixed ethnicity or other ethnic group	79 (1.8)	74 (1.8)	5 (1.0)
Not stated	128 (2.8)	119 (3.0)	9 (1.8)
Quintile of deprivation, n (%)			
1 (least deprived)	774 (17.3)	690 (17.4)	84 (17)
2	905 (20.3)	812 (20.4)	93 (18.8)
3	928 (20.8)	822 (20.7)	106 (21.4)
4	950 (21.3)	841 (21.2)	109 (22)
5 (most deprived)	912 (20.4)	809 (20.4)	103 (20.8)
Distance (km) from home to hospital, median (IQR)	33.1 (67.8) 9.3 (4.3 19.9) [4,475]	10 (4, 20)	8 (4, 16)
APACHE II severe co-morbidities, n (%)			
Liver	124 (2.8)	94 (2.3)	30 (6.0)
Renal	108 (2.4)	97 (2.4)	11 (2.2)
Respiratory	146 (3.2)	119 (3.0)	27 (5.4)
Cardiovascular	117 (2.6)	100 (2.5)	17 (3.4)
Metastatic cancer	121 (2.7)	110 (2.7)	11 (2.2)
Haematological malignancy	103 (2.3)	81 (2.0)	22 (4.4)
Immunocompromise	369 (8.2)	318 (7.9)	51 (10.2)
Prior dependency, n (%)			
Able to live without assistance	3,267 (72.5)	2,944 (73.5)	323 (64.7)
Minor or major assistance	1,171 (26.0)	1,004 (25.1)	167 (33.5)
Total assistance	47 (1.0)	42 (1.0)	5 (1.0)
Unknown	21 (0.5)	17 (0.4)	4 (0.8)
Surgical status *n* (%)			
Non-surgical	2,808 (62.3)	2,396 (59.8)	412 (82.6)
Planned admission following elective or scheduled surgery	702 (15.6)	686 (17.1)	16 (3.2)
Unplanned admission following surgery of any urgency	996 (22.1)	925 (23.1)	71 (14.2)
ICNARC Physiology Score, mean (SD)	18 (8.3)	18 (7.9)	26 (8.1)
APACHE II Score, mean (SD)	17 (6.3)	16 (6.1)	21 (6.2)
ICU length of stay (days), median (IQR)	4.9 (2.9 9.1)	4.8 (2.8, 9.0)	6.0 (3.6, 10.6)
Organ support received in the ICU, n (%)			
Advanced respiratory support	2,540 (56.4)	2,124 (53.0)	416 (83.4)
Advanced cardiovascular support	1,325 (29.4)	1,037 (25.9)	288 (57.7)

For peer review only

For peer review only - http://bmjopen.bmj.com/site/about/guidelines.xhtml

Renal support	691 (15.3)	510 (12.7)	181 (36.3)
Neurological support [a]	617 (13.7)	503 (12.6)	114 (22.8)
Duration (calendar days) of organ support among those receiving the support, median (IQR)			
Advanced respiratory support	5.0 (2.0 9.0)	4 (2, 9)	6 (4, 10)
Advanced cardiovascular support	3.0 (2.0 4.0)	2 (2, 4)	3 (2, 5)
Renal support	4.0 (3.0 8.0)	4 (3, 8)	4 (3, 8)
Neurological support	3.0 (2.0 7.0)	3 (2, 7)	3 (2, 5)
Death before acute hospital discharge, n (%)	852 (19.2)	353 (8.9)	N/A

[a] including admission receiving invasive neurological monitoring or treatment, continuous intravenous medication for seizures and/or cerebral monitoring, and therapeutic hypothermia using protocols and devices

Both overall and individual domain scores revealed generally high satisfaction (Table 3), however a long tail was present indicating some questionnaires were returned with very low scores (Figure 1). Family members of ICU non-survivors had higher scores for overall satisfaction and satisfaction with the decision-making process domain than family members of ICU survivors.

Table 3 Overall family satisfaction score for all family members and for family members by patient outcome

**Summary measures**	**All family members [N=7,017[a]]**	**Family members of ICU survivors [N=6,147[a]]**	**Family members of ICU non-survivors [N=870]**
Overall family satisfaction score			
**Median [IQR]**	83.3 [70.4, 93.0]	82.7 [69.9, 92.7]	87.1 [74.4, 94.8]
**Mean (SD)**	79.7 (16.7)	79.3 (16.5)	82.0 (17.5)
**[95% CI]**	[79.2 - 80.1]	[78.9 - 79.8]	[80.9 - 83.2]
Satisfaction with care domain score			
**Median [IQR]**	87.5 [74.3, 96.4]	87.5 [73.6, 96.4]	88.1 [76.8, 96.4]
**Mean (SD)**	83.1 (16.0)	83.0 (15.9)	83.8 (16.9)
**[95% CI]**	[82.7 - 83.4]	[82.6 - 83.4]	[82.7 - 84.9]
Satisfaction with information domain score			
**Median [IQR]**	79.2 [66.7, 95.8]	79.2 [62.5, 95.8]	83.3 [70.8, 100.0]
**Mean (SD)**	76.2 (22.0)	75.7 (22.0)	79.6 (22.9)
**[95% CI]**	[75.7 - 76.7]	[75.1 - 76.2]	[78.1 - 81.0]

For peer review only - http://bmjopen.bmj.com/site/about/guidelines.xhtml

For peer review only

Satisfaction with the decision-making process domain score			
**Median [IQR]**	75.6 [59.3, 93.1]	75.0 [57.5, 88.8]	87.5 [68.8, 100.0]
**Mean (SD)**	73.1 (22.3)	72.1 (22.0)	79.6 (22.9)
**[95% CI]**	[72.5 - 73.6]	[71.6 - 72.7]	[78.1 - 81.1]

[a] Two family members returned questionnaires but did not complete any of the 24 FS-ICU items – responses were not imputed for these family members.

Univariable analyses of the association between family satisfaction and family characteristics, patient characteristics, ICU/hospital characteristics and contextual factors are shown in the Supplementary Appendix (Table S3-S5). There was no evidence of differences in family satisfaction according to hospital teaching status or the number of beds in the ICU, however, these variables were retained in the multilevel multivariable models due to their controlling effect on the other coefficients in the models. A summary of the candidate variables considered in the models and a justification of their inclusion/exclusion is detailed in Table S6.

Results of the multivariable multilevel models for overall family satisfaction are shown in

Table 4. Among family members of ICU survivors, there was evidence of an association with overall family satisfaction for: family member age group; family member ethnicity; next-of-kin/lives with patient; frequency of visits; ICNARC Physiology Score; and receipt of advanced respiratory support. Among family members of non-survivors, only the following patient factors were significant: patient age; ICNARC Physiology Score; and ICU length of stay. These associations were significant when controlling for other predictors in the model. A priori-specified interaction terms and random slopes did not improve the fit of the models and so these terms were not retained.

Table 4 Multivariable multilevel models for overall family satisfaction score

**Variables**	**Family members of ICU survivors [N=6,143[a]]**			**Family members of ICU non-survivors [N=869[a]]**		
	Coef.	95% CI	p-value	Coef.	95% CI	p-value
Fixed effects – family member level						
Constant	68.30	(63.42, 73.17)		55.70	(42.26, 69.14)	
Family member age, years (vs <30)			0.041			0.18
30-39	1.97	(0.11, 3.82)		2.01	(-2.64, 6.66)	
40-49	1.65	(0.02, 3.29)		3.37	(-1.01, 7.75)	

For peer review only - http://bmjopen.bmj.com/site/about/guidelines.xhtml

50-59	1.96	(0.35, 3.56)		4.12	(-0.09, 8.33)	
60-69	1.35	(-0.31, 3.01)		4.26	(-0.25, 8.79)	
70-79	1.32	(-0.52, 3.17)		5.92	(0.69, 11.14)	
80+	-1.34	(-4.06, 1.37)		-0.18	(-6.80, 6.43)	
Family member sex – female (vs male)	0.32	(-0.48, 1.12)	0.44	0.66	(-1.45, 2.77)	0.54
Family member ethnicity – white (vs non-white)	3.59	(1.38, 5.80)	0.001	7.12	(-0.00, 14.25)	0.050
Next-of-kin/lives with patient (vs lives with patient)			<0.001			0.26
Next-of-kin, does not live with patient	-1.39	(-2.56, -0.22)		1.08	(-2.39, 4.55)	
Not next-of-kin, does not live with patient	-2.33	(-3.26, -1.41)		-1.24	(-3.88, 1.40)	
Frequent visitor	2.83	(1.82, 3.84)	<0.001	1.53	(-1.34, 4.39)	0.30
Fixed effects – patient level						
Patient age (per 10 years)	0.01	(-0.28, 0.31)	0.93	1.18	(0.09, 2.27)	0.033
Patient sex – female (vs male)	0.26	(-0.73, 1.25)	0.61	1.92	(-0.85, 4.70)	0.17
Dependency (vs none)			0.15			0.74
Minor or major	-0.30	(-1.60, 1.00)		-0.22	(-3.36, 2.92)	
Total	-4.62	(-9.32, 0.07)		4.98	(-8.10, 18.07)	
Surgical status (vs non-surgical)			0.63			0.82
Planned elective/scheduled	-0.74	(-2.24, 0.77)		-2.61	(-10.77, 5.54)	
Unplanned	-0.26	(-1.46, 0.94)		-0.08	(-3.95, 3.80)	
ICNARC Physiology Score (per point)	0.16	(0.09, 0.24)	<0.001	0.17	(0.00, 0.34)	0.045
ICU length of stay (per day)	-0.02	(-0.07, 0.03)	0.44	-0.30	(-0.46, -0.15)	<0.001
Advanced respiratory support	2.96	(1.80, 4.11)	<0.001	---		
Fixed effects – ICU/hospital level						
Hospital type (vs non-university)			0.49			0.55
University	0.86	(-3.61, 5.32)		-1.51	(-7.51, 4.50)	
University affiliated	1.97	(-1.26, 5.20)		1.77	(-2.55, 6.09)	
Number of ICU beds (per bed)	-0.00	(-0.23, 0.23)	0.97	0.26	(-0.08, 0.61)	0.13
Random effects – SD (SE)						
Between ICUs	2.91	(0.60)		2.81	(1.10)	
Within ICUs between patients	10.94	(0.29)		11.16	(0.69)	
Within patients between family members	11.98	(0.21)		12.26	(0.44)	
Variance partition – percentage						
Between ICUs	3%			2%		
Between patients	44%			44%		

Coef, coefficient; SE, standard error.

[a]Five patients had missing data on age group on both the questionnaire and web portal – due to the very small amount of missing data in this key variable, these missing values were not imputed.

For peer review only

For peer review only - http://bmjopen.bmj.com/site/about/guidelines.xhtml

Variances at both the patient and ICU/hospital levels were statistically significant but the variance partition coefficients (VPCs) at the ICU/hospital level were small in both the null and final multilevel models (4% and 3% for ICU survivors and 2% and 2% for ICU non-survivors, respectively), which means differences in overall family satisfaction scores were mainly at the patient and family member levels. Variance at the patient level represented 44% of the total variance in overall family satisfaction in the final models for family members of both ICU survivors and ICU non-survivors.

Full results of the multivariable multilevel models for the domain scores are reported in the Supplementary Appendix (Table S7-S9).

Figure 2 shows the funnel plots for the overall family satisfaction score, before and after adjustment for family member and patient characteristics from the multivariable multilevel models. Adjusting for family member and patient characteristics reduced the variability across ICUs, resulting in fewer ICUs outside the funnel plot control limits but the relative position of ICUs remained the same. Funnel plots for the individual domain scores before and after adjustment can be found in the Supplementary Appendix (Figure S2).

Sensitivity analyses

Multivariable multilevel models using the square root transformation of the satisfaction scores gave consistent results. In the models using imputed data, the direction and order of magnitude of coefficients that were significant were similar to those estimated using the traditional approach to scoring partially completed questionnaires (Supplementary Appendix, Table S10 and Table S11). On average, the multiple imputation approach tended to identify larger numbers of potential outliers due to the larger sample sizes and therefore narrower funnels.

Discussion

Overall and domain specific family satisfaction measured with the FS-ICU-24 was high. However, we found that scores vary significantly across adult general ICUs and that family members of patients who died in the ICU had higher levels of satisfaction. For family members of ICU survivors, characteristics of both the family member and the patient were significant determinants of family satisfaction, whereas, for family members of ICU non-survivors, only patient characteristics were significant. Adjustment for these family member and patient characteristics reduced the variation in family satisfaction across ICUs, resulting in fewer ICUs being identified as statistical outliers.

For peer review only - http://bmjopen.bmj.com/site/about/guidelines.xhtml

While the observational design of the FREE study precludes any causative inferences being made, we speculate that the higher levels of family satisfaction amongst family members of ICU non-survivors may be due to a number of factors, either singly or combined, including: greater involvement of the family in end-of-life decision making; family members of survivors having on-going issues to cope with following their family member's discharge from ICU; and/or other unknown factors. In order to fully identify and understand why family members of ICU non-survivors have higher family satisfaction, a detailed qualitative study is required.

The overall satisfaction score was comparable with other published studies employing similar methods to administer the FS-ICU-24 (14-17). Our findings are also consistent with a study by Wall et al (6) which identified that families of ICU non-survivors were more satisfied than families of ICU survivors. Similarly, Stricker et al (7), among a number of patient and ICU level factors studied, found that increasing acute severity of illness of the patient (evaluated using the SAPS II score) was associated with increasing satisfaction on the overall family satisfaction score, however, lower satisfaction was associated with ICU-level characteristics of a written admission/discharge policy and a higher patient:nurse ratio.

It is of note that one of largest magnitude associations in the FREE study was the finding that family members of white ethnicity, of both ICU survivors and non-survivors, had higher satisfaction than family members of other ethnicities. Further investigation of this issue is warranted to understand whether this reflects, for example, either cultural variation in family members' expectations or a need to engage better and communicate with family members who may not have English as their first language (17% of family members of other ethnicities indicated that their first language was not English compared with less than 1% of white ethnicity).

Our work has several important strengths. To our knowledge, this is the largest study assessing family satisfaction with ICU care. Nesting our study within the national clinical audit programme was efficient and novel and allowed for unbiased selection and stratification of participating units and linkage of family members' to patient data. One important strength is that the same mode and timing of delivery of the FS-ICU-24 was employed for family members of ICU survivors and non-survivors, avoiding potential sampling bias and allowing for meaningful comparisons between these groups. Finally, the large sample size of family members allowed for robust multilevel multivariable modelling of factors associated with overall family satisfaction to inform important adjustment of any future assessment using this questionnaire. Despite our very large sample size, we achieved a modest response rate (58%), however this was similar to other studies with smaller sample sizes (6, 14).

For peer review only - http://bmjopen.bmj.com/site/about/guidelines.xhtml

Our study does, however, have limitations. First, when assessing satisfaction, it is not uncommon for continuous measures to be skewed. Whilst the skewed nature of the satisfaction scores does not affect the parameter estimates in multilevel models (18, 19) it might cause problems when one is interested in the significance or in the confidence intervals of the variance terms at higher levels (19). In our analyses, we corrected the asymptotic standard errors using a robust (Huber/White) estimator to improve inference and performed a sensitivity analysis using a square root transformation which did not change our conclusions. Second, by excluding family members of patients who had spent less than 24 hours on ICU - to ensure that family members had spent long enough on ICU to feel able to respond to the questionnaire - we may have missed a small group of family members of very sick patients who died soon after admission to ICU. Third, there were differences in the case mix and outcome of patients between those who had at least one family member recruited and those who did not, leading to potential bias in the results. Fourth, we found that younger family members and those from non-white ethnicities were less likely to respond and important information may have been missed. Finally, 94% of patients were of white ethnicity, which is above that of the ethnic makeup of the UK (87%) and may make the overall family satisfaction scores less generalisable to other ethnicities.

In conclusion, this large, prospective, multicentre cohort study indicated that overall family satisfaction with adult general ICU care in the UK was high. However, our findings indicate that there is scope for some UK adult general ICUs to improve. Our results suggest that the FS-ICU-24 questionnaire could be used to audit family satisfaction but adjustment for differences in family member/patient characteristics is important to avoid falsely identifying ICUs as statistical outliers.

For peer review only

Acknowledgements

We wish to thank the NIHR Health Services and Delivery Research programme for funding this project. We also wish to thank all the patients, family members and staff from all the units that participated the study.

Research staff at sites

C Smalley and R Jacob (Arrowe Park Hospital); S Chau, S A Pearson, K Ellis and R Watmough (Barnsley Hospital); M Faulkner, L Evans and H Robertson (Countess of Chester Hospital); P Wakefield, R Abrahams, N Summers and H Wooldridge (Darent Valley Hospital); H McMillan, S Tyson, K Tantam, S Olver, C Brown and C Tippett (Derriford Hospital); S Moreton, S Jones, A Deeney, J Gibbins and A Oglesby (Dorset County Hospital); C Randell, M Allsop, K Harris, C Scott and C Boyd (Freeman Hospital); E Coughlan, A Jefferies and K Wylie (Manchester Royal Infirmary); C Plowright, C Pegg, L Cooper and T Hatton (Medway Maritime Hospital); P Doble, P Richards, D Bayford and K Adams (Musgrove Park Hospital); J Spimpolo, M Burt and R Pillai (Northampton General Hospital); K A Simeson and S Buckley (Pinderfields Hospital); A Jackson, M Nadolski and H Baker (Royal Devon & Exeter Hospital, Wonford); N Mason, U Gunter and L Roberts (Royal Gwent Hospital); T Evans, E Cooke, M Ogden and P Dark (Salford Royal Hospital); M Cody, F Hogg and D McCahery (South West Acute Hospital); D Dawson, J Mellinghoff, S Prudden, N Poonuth and C Ryan (St George's Hospital); G Mandersloot, A Smith (The Royal London Hospital); S Hagan, L Humphries and E Murphy (Ulster Hospital); E Walker, H Payne and X Zhao (Watford General Hospital); C Edmondson, S Anglesea and H Williams (Wrexham Maelor Hospital).

Study Steering Committee

Dr Kathleen Daly (independent chair); Andrina Colquoun (independent); Dr Maureen Dalziel; Kirsty Everingham (independent); Doreen Henry (independent); Joan Pearson (independent); Catherine Plowright; Dr Laura Price (independent); Professor Kathryn Rowan; Professor Mervyn Singer (independent); and Dr Stephen Wright.

Funding: This project was funded by the National Institute for Health Research (NIHR) Health Services and Delivery Research (HS&DR) Programme (11/2003/56). The funder had no involvement in study design; in collection, analysis and interpretation of data; in the writing of the report; or in the decision to submit the article for publication. The views and opinions expressed therein are those of the authors and do not necessarily reflect those of the HS&DR Programme, NIHR, NHS or the Department of Health.

For peer review only - http://bmjopen.bmj.com/site/about/guidelines.xhtml

Competing interests: Kathryn M Rowan is a member of the NIHR HS&DR Board. Elaine McColl was an editor for the NIHR Journals Library between 2013 and 2016 and her employers received a fee for this work. The other authors declare no conflicts of interest. All authors have completed the Unified Competing Interest form (available on request from the corresponding author).

Data sharing: data can be obtained from the corresponding author on request

Authors contributions: KMR as Chief Investigator conceived the idea and designed the study with DAH, SHE, DKH, LH, EMc, MR, AR, and SEW. EW co-ordinated the study and contributed to data acquisition with ARB, RRC, SS, SHE, AR, and SEW. PFV, DWG, DAH, SHE, DKH, LH, EMc, MR, SEW, and KMR were involved in the analysis and interpretation of the results. All authors were involved in drafting, editing and have approved the final manuscript.

For peer review only

For peer review only - http://bmjopen.bmj.com/site/about/guidelines.xhtml

References

1. Black N, Jenkinson C. Measuring patients' experiences and outcomes. BMJ. 2009;339:b2495.
2. van den Broek JM, Brunsveld-Reinders AH, Zedlitz AM, et al. Questionnaires on Family Satisfaction in the Adult ICU: A Systematic Review Including Psychometric Properties. Crit Care Med. 2015;43(8):1731-44.
3. Heyland DK, Tranmer JE. Measuring family satisfaction with care in the intensive care unit: the development of a questionnaire and preliminary results. J Crit Care. 2001;16(4):142-9.
4. Rothen HU, Stricker KH, Heyland DK. Family satisfaction with critical care: measurements and messages. Curr Opin Crit Care. 2010;16(6):623-31.
5. Wall RJ, Engelberg RA, Downey L, et al. Refinement, scoring, and validation of the Family Satisfaction in the Intensive Care Unit (FS-ICU) survey. Crit Care Med. 2007;35(1):271-9.
6. Wall RJ, Curtis JR, Cooke CR, et al. Family satisfaction in the ICU: differences between families of survivors and nonsurvivors. Chest. 2007;132(5):1425-33.
7. Stricker KH, Kimberger O, Schmidlin K, et al. Family satisfaction in the intensive care unit: what makes the difference? Intensive Care Med. 2009;35(12):2051-9.
8. Wright SE, Walmsley E, Harvey SE, et al. Family-Reported Experiences Evaluation (FREE) study: a mixed-methods study to evaluate families' satisfaction with adult critical care services in the NHS. Health Serv Deliv Res. 2015;3(45).
9. DA H, P F-V, SE W, et al. Psychometric assessment of the Family Satisfaction in the Intensive Care Unit questionnaire in the United Kingdom. J Crit Care. 2017;38:346-50.
10. Snijders TAB, Bosker RJ. Multilevel Analysis: An Introduction to Basic and Advanced Multilevel Modeling. Thousand Oaks, CA: SAGE Publications; 1999.
11. Spiegelhalter DJ. Funnel plots for comparing institutional performance. Stat Med. 2005;24(8):1185-202.
12. Carpenter JR, Goldstein H, Kenward MG. REALCOM-IMPUTE software for multilevel multiple imputation with mixed response types. Journal of Statistical Software. 2011;45:5.
13. von Elm E, Altman DG, Egger M, et al. Strengthening the Reporting of Observational Studies in Epidemiology (STROBE) statement: guidelines for reporting observational studies. BMJ. 2007;335(7624):806-8.
14. Dodek PM, Wong H, Heyland DK, et al. The relationship between organizational culture and family satisfaction in critical care. Crit Care Med. 2012;40(5):1506-12.
15. Khalaila R. Patients' family satisfaction with needs met at the medical intensive care unit. Journal of Advanced Nursing. 2013(69):1172-82.
16. Schwarzkopf D, Behrend S, Skupin H, et al. Family satisfaction in the intensive care unit: a quantitative and qualitative analysis. Intensive Care Med. 2013;39(6):1071-9.
17. Tastan S, Iyigun E, Ayhan H, et al. Validity and reliability of Turkish version of family satisfaction in the intensive care unit. Int J Nurs Pract. 2014;20(3):320-6.
18. Gelman A HJ. Data Analysis Using Regression and Multilevel/Hierarchical Models. Cambridge: Cambridge University Press; 2007.
19. Maas CJM, Hox JJ. The influence of violations of assumptions on multilevel parameter estimates and their standard errors. Computational Statistics & Data Analysis. 2004;46(3):427-40.

For peer review only - http://bmjopen.bmj.com/site/about/guidelines.xhtml

Figure legends

Figure 1 Distribution of overall family satisfaction score

Figure 2 Variation across ICUs in the mean overall family satisfaction score (A) before and (B) after adjustment for patient and family member characteristics

For peer review only

For peer review only - http://bmjopen.bmj.com/site/about/guidelines.xhtml

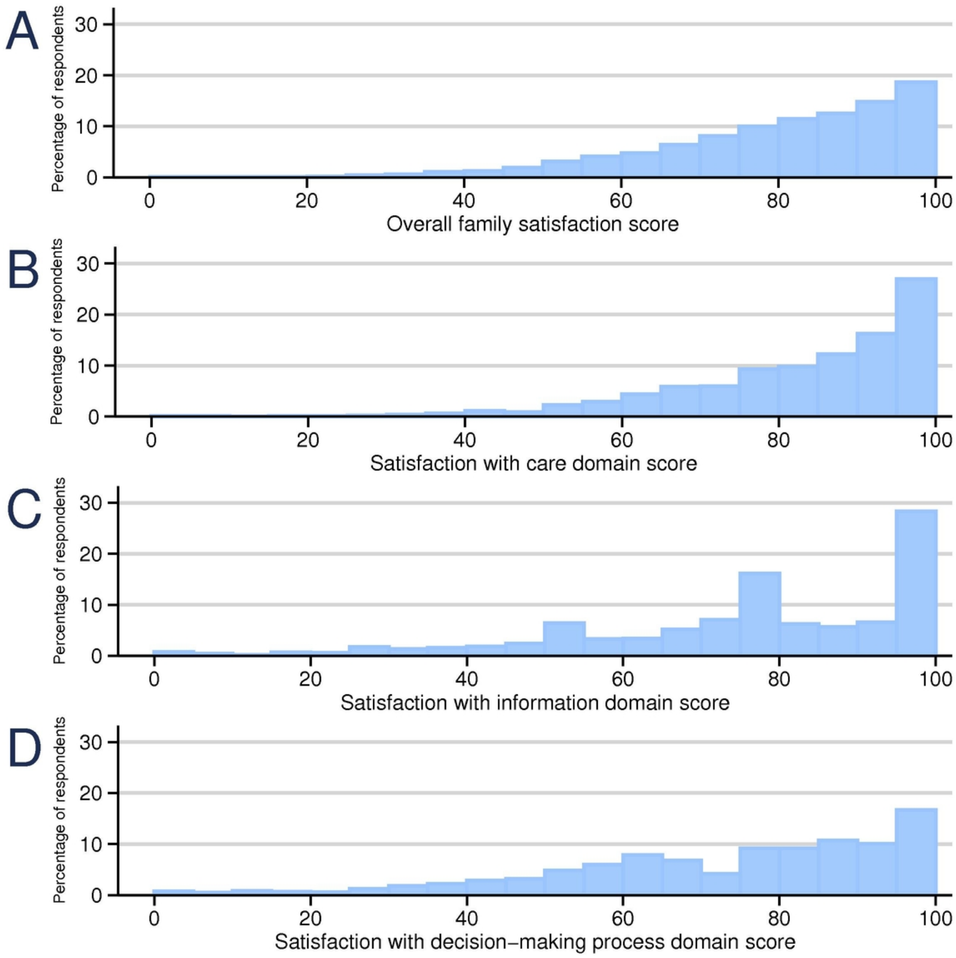

101x101mm (300 x 300 DPI)

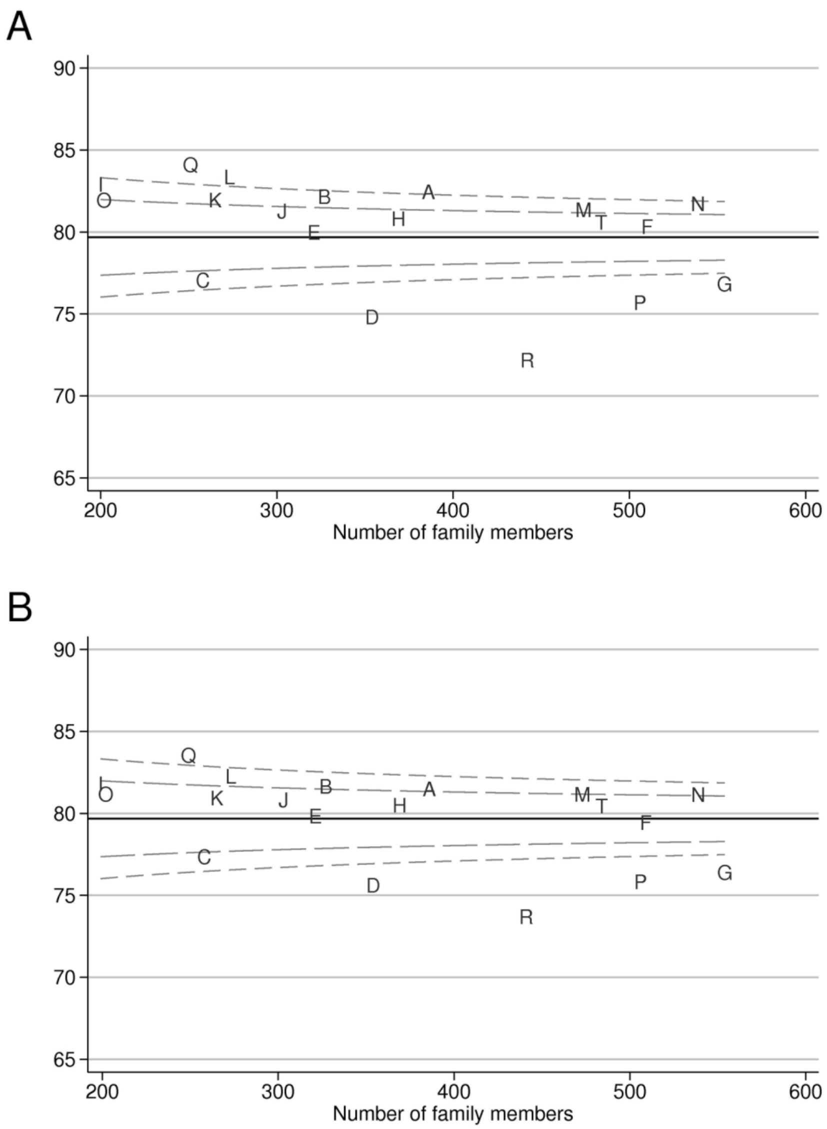

127x177mm (300 x 300 DPI)

Supplementary material

Family satisfaction with critical care in the United Kingdom: a multi-centre cohort study

For peer review only

Table S1 Characteristics and outcomes for all admission to ICUs participating in the FREE study and ICNARC Case Mix Programme

	**CMP**	**FREE study**
Total number of ICUs [N]	[209][a]	[19][a]
Total number of admissions [N]	[149,779]	[18,270]
Age *mean* (SD)	61.5 (18.0)	61.5 (18.0)
Sex *male* (%)	82,444 (55.0)	10,316 (56.5)
Ethnicity *n* (%)		
White	135,767 (90.6)	16,439 (90.0)
Asian	4,815 (3.2)	439 (2.4)
Black	3,250 (2.2)	327 (1.8)
Other	2,434 (1.6)	445 (2.4)
Not stated	3,513 (2.3)	620 (3.4)
Distance (km) from patient home to hospital *median* (IQR) [N]	25.0 (54.2) 8.7 (3.9 19.3) [128,169]	31.7 (64.5) 9.2 (4.2 20.8) [18,090]
APACHE II severe co-morbidities *n* (%)		
0	123,437 (82.4)	14,742 (80.7)
1	20,906 (14.0)	2,648 (14.5)
2	5,053 (3.4)	793 (4.3)
3 or more	383 (0.3)	87 (0.5)
Admission type *n* (%) [N]	[149,765]	[18,270]
Medical	87,940 (58.7)	10,039 (54.9)
Elective surgery	34,284 (22.9)	4,761 (26.1)
Emergency surgery	27,541 (18.4)	3,470 (19.0)
Surgical status of surgical admissions *n* (%) [N]	[61,825]	[8,231]
Planned surgery	28,267 (45.7)	3,985 (48.4)
Unplanned surgery	33,558 (54.3)	4,246 (51.6)
ICNARC Physiology Score *mean* (SD)	16.9 (9.3)	16.5 (9.2)
ICNARC predicted risk of death *median* (IQR) [N]	0.10 (0.03 0.33) [142,654]	0.09 (0.03 0.30) [17,261]
APACHE II Acute Physiology Score *mean* (SD)	11.4 (6.1)	11.3 (5.9)
APACHE II Score *mean* (SD)	15.7 (7.0)	15.6 (6.9)
APACHE II predicted risk of death *median* (IQR) [N]	0.12 (0.04 0.29) [132,197]	0.11 (0.04 0.28) [16,193]
Mechanical ventilation during first 24 hrs *n* (%) [N]	58,687 (39.4) [148,975]	7,008 (38.5) [18,187]

For peer review only

ICU mortality *n* (%) [N]	21,505 (14.4) [149,779]	2,560 (14.0) [18,270]
Acute hospital mortality *n* (%) [N]	29,945 (21.0) [142,670]	3,550 (20.6) [17,266]

[a] excludes one ICU for which no CMP data were available

For peer review only

Figure S1 Overview of patients, family members and questionnaires (distributed/returned)

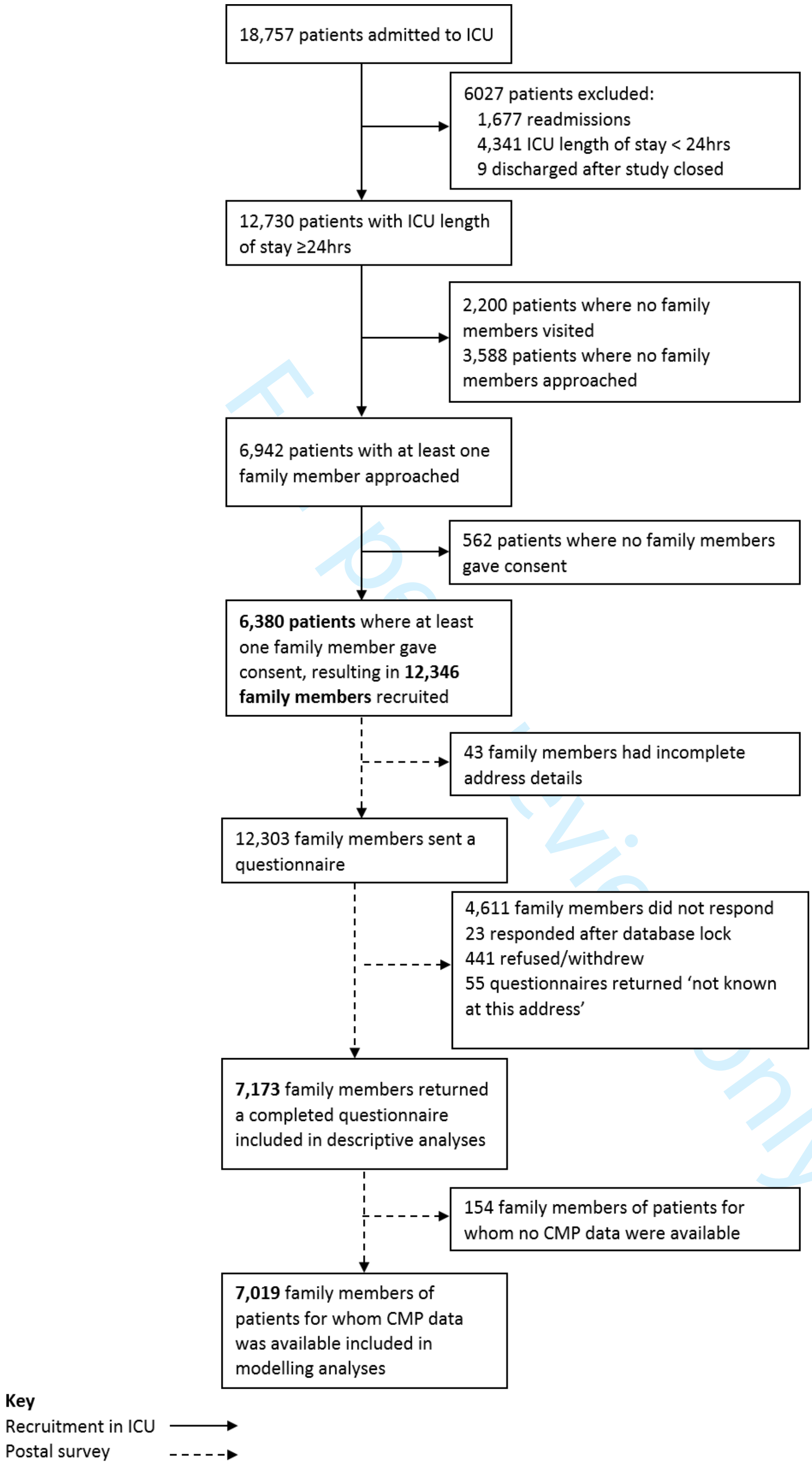

Table S2 Characteristics of all recruited family members and by response to questionnaire

	All recruited family members	Those returning questionnaires	Did not respond
Total number of family members, N	12 346	7173	4611
Age group, *n* (%) [N]	[12 068]	[7019]	[4500]
<30	1429 (11.8)	530 (7.6)	861 (19.1)
30-39	1590 (13.2)	721 (10.3)	827 (18.4)
40-49	2760 (22.9)	1465 (20.9)	1208 (26.9)
50-59	2646 (21.9)	1654 (23.6)	886 (19.7)
60-69	2131 (17.7)	1580 (22.5)	440 (9.8)
70-79	1211 (10.0)	862 (12.3)	220 (4.8)
80+	301 (2.5)	207 (2.9)	58 (1.3)
Sex, *n* (%) [N]	[12 145]	[7062]	[4529]
Female	7687 (63.3)	4689 (66.4)	2663 (58.8)
Male	4458 (36.7)	2373 (33.6)	1866 (41.2)
Ethnicity, *n* (%) [N]	[12 090]	[7033]	[4505]
White	11 379 (94.1)	6747 (95.9)	4111 (91.3)
Asian	355 (2.9)	142 (2.0)	196 (4.4)
Black	161 (1.3)	55 (0.8)	101 (2.2)
Other	195 (1.6)	89 (1.3)	97 (2.1)
Deprivation, *n* (%) [N]	[11 740]	[6832]	[4370]
1 [least deprived]	2113 (18.0)	1376 (20.1)	634 (14.5)
2	2406 (20.5)	1502 (22.0)	803 (18.4)
3	2415 (20.6)	1443 (21.1)	851 (19.5)
4	2545 (21.7)	1380 (20.2)	1045 (23.9)
5 [most deprived]	2261 (19.3)	1131 (16.6)	1037 (23.7)
Distance (km) from family member home to hospital, *median* (IQR) [N]	11.6 (5.1-30.7) [11 803]	12.3 (5.3-33.2) [6867]	10.7 (4.6-29.4) [4394]
Relationship, *n* (%) [N] "I am the patient's..."	[12 343]	[7173]	[4611]
Partner	3105 (25.2)	2151 (30.0)	786 (17.0)
Child	4186 (33.9)	2292 (32.0)	1780 (38.6)
Parent	1054 (8.5)	665 (9.3)	338 (7.3)
Sibling	1271 (10.3)	717 (10.0)	480 (10.4)
Other relative	1973 (16.0)	987 (13.8)	898 (19.5)
Other non-relative	754 (6.1)	361 (5.0)	329 (7.1)
Next-of-kin, *n* (%) [N]	[11 702]	[6770]	[4389]
No	7086 (60.6)	3747 (55.3)	3009 (68.6)
Yes	4616 (39.4)	3023 (44.7)	1380 (31.4)
Lives with patient, *n* (%) [N]	[12 343]	[7172]	[4609]
No	8255 (66.9)	4543 (63.3)	3357 (72.8)
Yes	4088 (33.1)	2629 (36.7)	1252 (27.2)
Education level, *n* (%) [N]	[10 293]	[5971]	[3888]
NVQ 1 or 2	3147 (30.6)	1731 (29.0)	1284 (33.0)
NVQ 3	2086 (20.3)	1149 (19.2)	870 (22.4)

For peer review only

NVQ 4 or 5	2936 (28.5)	1819 (30.5)	1032 (26.5)
Other	2124 (20.6)	1272 (21.3)	702 (18.1)
First language, *n* (%) [N]	[12 346]	[7 173]	[4611]
Not English	335 (2.7)	140 (2.0)	182 (3.9)
English	12 011 (97.3)	7 033 (98.0)	4429 (96.1)

For peer review only

Table S3 Univariable analyses of factors associated with overall family satisfaction score by ICU outcome – family member characteristics

Variables	Family members of ICU survivors [N=6,147[a]]			Family members of ICU non-survivors [N=870]		
	Coef.	95% CI	p-value	Coef.	95% CI	p-value
Age, years (vs < 30)			0.031			0.033
30-39	1.56	(-0.22, 3.33)		2.68	(-1.80, 7.17)	
40-49	0.42	(-0.10, 0.94)		1.61	(0.21, 3.01)	
50-59	2.12	(0.61, 3.64)		5.49	(1.49, 9.50)	
60-69	1.96	(0.39, 3.52)		6.01	(1.78, 10.25)	
70-79	1.98	(0.28, 3.68)		7.39	(2.58, 12.19)	
80+	-0.55	(-3.05, 1.95)		2.62	(-3.48, 8.73)	
Female (vs male)	0.40	(-0.34, 1.14)	0.29	0.44	(-1.59, 2.47)	0.67
White ethnicity (vs non-white)	3.60	(1.46, 5.75)	0.001	8.78	(1.85, 15.70)	0.013
Relationship (vs partner)			<0.001			0.28
Parent	0.00	(-1.39, 1.39)		0.08	(-5.73, 5.90)	
Child	-0.94	(-1.83, -0.05)		-1.274	(-3.69, 1.14)	
Sibling	-2.16	(-3.50, -0.82)		0.909	(-3.02, 4.84)	
Other-relative	-1.63	(-2.81, -0.44)		-0.619	(-3.60, 2.36)	
Other-non relative	-3.42	(-5.22, -1.62)		-6.134	(-11.69, -0.58)	
Next of kin	1.74	(1.05, 2.44)	<0.001	2.69	(0.78, 4.59)	0.006
Lives with patient	1.95	(1.20, 2.69)	<0.001	1.15	(-0.99, 3.29)	0.29
Education (vs NVQ 1 or 2)			<0.001			0.16
NVQ 3	-0.60	(-1.77, 0.57)		1.14	(-2.09, 4.37)	
NVQ 4 or 5	-2.43	(-3.49, -1.37)		-2.07	(-4.92, 0.77)	
Other	-0.18	(-1.35, 0.98)		-1.75	(-4.73, 1.24)	
Quintile of deprivation (vs 1, least deprived)			0.63			0.77
2	0.49	(-0.74, 1.72)		0.64	(-2.73, 4.01)	
3	0.96	(-0.29, 2.20)		0.84	(-2.59, 4.26)	
4	0.32	(-0.97, 1.60)		-1.07	(-4.59, 2.44)	
5 (most deprived)	0.67	(-0.70, 2.05)		0.79	(-3.10, 4.69)	
Distance from home to hospital (per 10 km)	-0.05	(-0.11, 0.01)	0.12	0.05	(-0.09, 0.18)	0.49
Previous experience of ICU as a family member	0.25	(-0.63, 1.14)	0.58	-0.68	(-3.22, 1.87)	0.60
Frequent visitor	2.52	(1.63, 3.41)	<0.001	2.91	(0.36, 5.47)	0.030

Coef., coefficient.

[a] Two family members returned questionnaires but did not complete any of the 24 FS-ICU items – responses were not imputed for these family members.

For peer review only

Table S4 Univariable analyses of factors associated with overall family satisfaction score by ICU outcome – patient characteristics

Variables	Family members of ICU survivors [N=6,147[a]]			Family members of ICU non-survivors [N=870]		
	Coef.	95% CI	p-value	Coef.	95% CI	p-value
Age (per 10 years)	-0.09	(-0.36, 0.17)	0.49	1.12	(0.11, 2.14)	0.030
Female (vs male)	0.67	(-0.25, 1.59)	0.16	2.04	(-0.66, 4.74)	0.14
White ethnicity (vs non-white)	2.39	(0.11, 4.68)	0.040	9.25	(2.38, 16.12)	0.008
Quintile of deprivation (vs 1, least deprived)			0.76			0.95
2	0.86	(-0.66, 2.38)		-1.28	(-5.85, 3.29)	
3	0.62	(-0.90, 2.13)		-0.68	(-5.12, 3.75)	
4	0.77	(-0.75, 2.28)		-1.62	(-6.03, 2.78)	
5 (most deprived)	1.00	(-0.57, 2.57)		-1.49	(-6.04, 3.06)	
Distance from home to hospital (per 10 km)	0.12	(0.00, 0.24)	0.047	0.18	(-0.05, 0.41)	0.12
Severe comorbidities						
Liver	3.18	(-0.01, 6.38)	0.050	1.25	(-4.67, 7.19)	0.68
Renal	-0.45	(-3.57, 2.66)	0.77	-8.87	(-18.35, 0.60)	0.067
Respiratory	0.01	(-2.84, 2.85)	1.00	-1.02	(-7.23, 5.19)	0.75
Cardiovascular	-0.14	(-3.23, 2.94)	0.93	1.40	(-6.46, 9.26)	0.73
Metastatic cancer	-2.81	(-5.78, 0.15)	0.063	3.26	(-6.38, 12.90)	0.51
Haematological malignancy	2.25	(-1.09, 5.61)	0.19	-7.88	(-14.62, -1.13)	0.022
Immunocompromise	-0.91	(-2.74, 0.90)	0.33	-3.90	(-8.55, 0.74)	0.10
Dependency (vs none)			0.30			0.85
Minor or major	-0.14	(-1.36, 1.08)		0.63	(-2.34, 3.60)	
Total	-3.63	(-8.21, 0.94)		2.73	(-10.21, 15.67)	
Surgical status (vs non-surgical)			0.005			0.78
Planned elective/scheduled	-2.17	(-3.51, -0.83)		-2.83	(-10.75, 5.10)	
Unplanned	-0.17	(-1.29, 0.96)		-0.06	(-3.89, 3.76)	
ICNARC Physiology Score (per point)	0.19	(0.13, 0.25)	<0.001	0.19	(0.02, 0.35)	0.026
ICU length of stay (per day)	0.02	(-0.03, 0.06)	0.44	-0.34	(-0.48, -0.20)	<0.001
Advanced respiratory support	3.62	(2.63, 4.61)	<0.001	1.96	(-1.84, 5.76)	0.31
Advanced cardiovascular support	2.06	(0.89, 3.22)	0.001	0.83	(-2.06, 3.72)	0.58
Renal support	1.52	(0.11, 2.93)	0.034	0.04	(-2.83, 2.91)	0.98
Neurological support	1.96	(0.39, 3.54)	0.014	2.95	(-0.42, 6.32)	0.086
Duration of adv. respiratory support (per day)	0.11	(0.05, 0.16)	<0.001	-0.16	(-0.32, 0.00)	0.051
Duration of adv. cardiovascular support (per day)	0.40	(0.15, 0.65)	0.002	0.11	(-0.33, 0.56)	0.62
Duration of renal support (per day)	0.16	(0.00, 0.32)	0.048	-0.15	(-0.43, 0.13)	0.28
Duration of neurological support (per day)	0.10	(-0.09, 0.29)	0.31	0.05	(-0.43, 0.53)	0.84
Death before acute hospital discharge	-0.49	(-1.52, 0.55)	0.36	N/A		

Coef., coefficient.

For peer review only

[a] Two family members returned questionnaires but did not complete any of the 24 FS-ICU items – responses were not imputed for these family members.

For peer review only

Table S5 Univariable analysis of factors associated with overall family satisfaction score by ICU outcome – ICU/hospital characteristics and contextual factors

**Variables**	**Family members of ICU survivors [N=6,147[a]]**			**Family members of ICU non-survivors [N=870]**		
	Coef.	95% CI	p-value	Coef.	95% CI	p-value
Hospital type (vs non-university)			0.51			0.62
University	0.06	(-3.63, 3.75)		-0.32	(-4.72, 4.07)	
University affiliated	1.93	(-1.56, 5.42)		1.68	(-2.29, 5.65)	
Number of ICU beds (per bed)	-0.05	(-0.23, 0.14)	0.63	0.02	(-0.22, 0.26)	0.85
Month of ICU admission (vs January)			0.95			0.85
February	-0.61	(-2.87, 1.65)		-0.03	(-6.90, 6.83)	
March	0.09	(-2.12, 2.30)		-0.06	(-6.73, 6.60)	
April	0.54	(-1.71, 2.79)		0.07	(-6.93, 7.07)	
May	-0.06	(-2.31, 2.18)		0.73	(-5.62, 7.08)	
June	-0.66	(-2.65, 1.34)		0.84	(-4.95, 6.64)	
July	0.85	(-1.41, 3.11)		3.91	(-2.71, 10.52)	
August	0.65	(-1.64, 2.93)		-0.70	(-6.87, 5.46)	
September	0.09	(-2.14, 2.31)		1.74	(-4.76, 8.25)	
October	0.44	(-1.76, 2.63)		1.15	(-5.69, 7.98)	
November	0.60	(-1.65, 2.85)		2.21	(-4.10, 8.53)	
December	0.69	(-1.57, 2.96)		5.16	(-1.13, 11.46)	
Questionnaire received while patient still in hospital	0.087	(-1.50, 1.67)	0.91	N/A		

Coef., coefficient.

[a] Two family members returned questionnaires but did not complete any of the 24 FS-ICU items – responses were not imputed for these family members.

For peer review only

Table S6 Sensitivity analyses –candidate determinants for the multivariable multilevel models for the family satisfaction in the intensive care unit

Candidate determinants	Justification inclusion/exclusion	Approach to modelling
**Family member level**		
Education level	It was not considered in the multivariable models due to higher than expected proportions of both "Not stated" (17%) and "Other" (21%) responses, suggesting a lack of comprehension of the categorisation used.	
Distance from home to hospital	No significant after adjusting for other variables in the model. It was dropped.	
Family member age, years	Controlling effect	Categorical (<30;30-39;40-49;50-59;60-69;70-79;80+)
Family member sex	Controlling effect	Categorical (male; female)
Family member ethnicity	Statistically significant in univariable	Categorical (white; non-white)
Next-of-kin/lives with patient	There was a strong multicollinearity between relationship to the patient and the other key variables of next-of-kin and lives with patient.	Categorical (lives with patient; Next-of-kin, does not live with patient; Not next-of-kin, does not live with patient)
Frequent visitor	Statistically significant in univariable	Binary (yes; no)
**Patient level**		
Patient ethnicity	It was not carried forward to the multivariable models due to collinearity with family member ethnicity.	
Patient age	Controlling effect	Continuous(linear)
Patient sex	Controlling effect	Categorical (male; female)
Dependency	Controlling effect	Categorical (none; minor or major; total)
Surgical status (vs non-surgical)	Controlling effect	Categorical (non-surgical; planned elective/scheduled; unplanned)
ICNARC Physiology Score	Statistically significant in univariable	Continuous(linear)
ICU length of stay (days)		Continuous(linear)
Organ support received in the ICU and duration (calendar days)	Once included in the multivariable model for	

For peer review only

of organ support among those receiving the support	survivors, only advanced respiratory support remained significant.	
Advanced respiratory support	It was found to be preferable to alternative variable of the duration of advanced respiratory support, which was correlated with ICU length of stay.	Binary (yes; no)
haematological malignancy	No significant after adjusting for other variables in the model. It was dropped.	
**ICU/hospital level**		
Hospital type	Controlling effect	Categorical (non-university; university; university affiliated)
Number of ICU beds	Controlling effect	Continuous(linear)

For peer review only

Table S7 Multivariable multilevel models for the satisfaction with care domain score

**Variables**	**Family members of ICU survivors [N=6,143[a]]**			**Family members of ICU non-survivors [N=869[a]]**		
	Coef.	95% CI	p-value	Coef.	95% CI	p-value
Fixed effects – family member level						
Constant	71.45	(66.67, 76.22)		55.29	(41.76, 68.82)	
Family member age, years (vs <30)			0.001			0.16
30-39	2.60	(0.81, 4.38)		2.50	(-1.97, 6.97)	
40-49	2.73	(1.16, 4.31)		4.31	(0.09, 8.54)	
50-59	2.91	(1.36, 4.44)		4.99	(0.93, 9.04)	
60-69	2.67	(1.08, 4.26)		4.89	(0.54, 9.23)	
70-79	2.66	(0.90, 4.41)		5.91	(0.88, 10.94)	
80+	-0.17	(-2.76, 2.41)		1.85	(-4.51, 8.21)	
Family member sex – female (vs male)	0.42	(-0.35, 1.20)	0.29	0.22	(-1.81, 2.25)	0.83
Family member ethnicity – white (vs non-white)	3.87	(1.77, 5.97)	<0.001	6.99	(0.19, 13.81)	0.044
Next-of-kin/lives with patient (vs lives with patient)			<0.001			0.15
Next-of-kin, does not live with patient	-1.14	(-2.26, -0.02)		0.95	(-2.39, 4.29)	
Not next-of-kin, does not live with patient	-2.44	(-3.32, -1.55)		-1.58	(-4.11, 0.94)	
Frequent visitor	2.49	(1.52, 3.46)	<0.001	1.49	(-1.27, 4.25)	0.29
Fixed effects – patient level						
Patient age (per 10 years)	0.03	(-0.25, 0.31)	0.83	1.21	(0.16, 2.26)	0.024
Patient sex – female (vs male)	0.06	(-0.85, 0.98)	0.87	1.85	(-0.79, 4.5)	0.17
Dependency (vs none)			0.006			0.68
Minor or major	-0.74	(-1.96, 0.46)		-0.94	(-3.98, 2.09)	
Total	-6.77	(-11.18, -2.36)		3.62	(-8.71, 15.95)	
Surgical status (vs non-surgical)			0.68			0.47
Planned elective/scheduled	-0.62	(-2.04, 0.78)		-4.85	(-12.71, 2.99)	
Unplanned	-0.15	(-1.27, 0.95)		-0.57	(-4.29, 3.13)	
ICNARC Physiology Score (per point)	0.14	(0.07, 0.21)	<0.001	0.14	(-0.03, 0.30)	0.10
ICU length of stay (per day)	-0.02	(-0.06, 0.02)	0.39	-0.30	(-0.45, -0.15)	<0.001
Advanced respiratory support	2.74	(1.66, 3.82)	<0.001			
Fixed effects – ICU/hospital level						
Hospital type (vs non-university)			0.51			0.58

For peer review only

University	0.94	(-3.58, 5.47)		-1.48	(-7.8 , 4.84)	
University affiliated	1.92	(-1.34, 5.19)		1.79	(-2.75, 6.34)	
Number of ICU beds (per bed)	-0.01	(-0.24, 0.23)	0.96	0.24	(-0.12, 0.59)	0.19
Random effects – SD (SE)						
Between ICUs	2.98	(0.60)		3.25	(1.11)	
Within ICUs between patients	9.76	(0.28)		10.47	(0.66)	
Within patients between family members	11.96	(0.19)		11.92	(0.42)	

Coef, coefficient; SE, standard error.

[a] Five patients were missing age group on both the questionnaire and web portal – due to the very small amount of missing data in this key variable, these missing values were not imputed.

For peer review only

Table S8 Multivariable multilevel models for the satisfaction with information domain score

**Variables**	**Family members of ICU survivors [N=6,143[a]]**			**Family members of ICU non-survivors [N=869[a]]**		
	Coef.	95% CI	p-value	Coef.	95% CI	p-value
Fixed effects – family member level						
Constant	66.07	(59.78, 72.21)		55.86	(39.34, 72.38)	
Family member age, years (vs <30)			0.63			0.28
30-39	0.28	(-2.22, 2.79)		1.23	(-4.92, 7.39)	
40-49	0.00	(-2.21, 2.21)		1.88	(-3.92, 7.68)	
50-59	0.55	(-1.62, 2.72)		2.88	(-2.70, 8.48)	
60-69	-0.1	(-2.35, 2.14)		4.24	(-1.71, 10.2)	
70-79	-0.41	(-2.89, 2.08)		6.43	(-0.45, 13.31)	
80+	-2.67	(-6.35, 1.01)		-1.96	(-10.71, 6.79)	
Family member sex – female (vs male)	0.20	(-0.89, 1.30)	0.72	1.01	(-1.81, 3.82)	0.49
Family member ethnicity – white (vs non-white)	4.73	(1.78, 7.68)	0.002	9.34	(0.47, 18.21)	0.039
Next-of-kin/lives with patient (vs lives with patient)			<0.001			0.38
Next-of-kin, does not live with patient	-2.39	(-3.97, 0.81)		1.43	(-3.09, 5.95)	
Not next-of-kin, does not live with patient	-2.57	(-3.83, 1.31)		-1.21	(-4.69, 2.28)	
Frequent visitor	2.11	(0.74, 3.48)	0.002	0.44	(-3.33, 4.22)	0.82
Fixed effects – patient level						
Patient age (per 10 years)	-0.22	(-0.61, 0.18)	0.28	0.92	(-0.43, 2.27)	0.18
Patient sex – female (vs male)	0.32	(-0.98, 1.62)	0.63	1.93	(-1.48, 5.35)	0.27
Dependency (vs none)			0.61			0.51
Minor or major	-0.49	(-2.2, 1.2)		-0.28	(-4.11, 3.53)	
Total	-2.69	(-8.92, 3.52)		9.15	(-6.57, 24.87)	
Surgical status (vs non-surgical)			0.88			0.84
Planned elective/scheduled	-0.32	(-2.32, 1.66)		-0.88	(-10.97, 9.21)	
Unplanned	0.23	(-1.33, 1.80)		-1.4	(-6.16, 3.36)	
ICNARC Physiology Score (per point)	0.23	(0.13, 0.33)	<0.001	0.15	(-0.04, 0.36)	0.13
ICU length of stay (per day)	-0.05	(-0.11, 0.01)	0.14	-0.43	(-0.62, -0.24)	<0.001
Advanced respiratory support	3.34	(1.83, 4.85)	<0.001	--		
Fixed effects – ICU/hospital level						
Hospital type (vs non-university)			0.45			0.58

For peer review only

University	1.69	(-3.71, 7.08)		0.35	(-6.42, 7.13)	
University affiliated	2.48	(-1.42, 6.40)		2.53	(-2.32, 7.39)	
Number of ICU beds (per bed)	-0.03	(-0.31, 0.24)	0.81	0.21	(-0.17, 0.61)	0.27
Random effects – SD (SE)						
Between ICUs	3.48	(0.73)		2.81	(1.37)	
Within ICUs between patients	13.64	(0.41)		12.38	(0.97)	
Within patients between family members	16.88	(0.27)		17.02	(0.60)	

Coef, coefficient; SE, standard error.

[a] Five patients were missing age group on both the questionnaire and web portal – due to the very small amount of missing data in this key variable, these missing values were not imputed.

For peer review only

Table S9 Multivariable multilevel models for the satisfaction with the decision-making process domain score

Variables	Family members of ICU survivors [N=6,143[a]]			Family members of ICU non-survivors [N=869[a]]		
	Coef.	95% CI	p-value	Coef.	95% CI	p-value
Fixed effects – family member level						
Constant	61.65	(55.17, 68.14)		39.62	(20.14, 59.09)	
Family member age, years (vs <30)			0.061			0.40
30-39	1.66	(-1.63, 4.95)		1.37	(-5.35, 8.10)	
40-49	0.02	(-2.76, 2.82)		2.73	(-3.47, 8.95)	
50-59	0.52	(-2.21, 3.25)		3.34	(-2.61, 9.31)	
60-69	-1.43	(-4.48, 1.61)		3.35	(-3.05, 9.77)	
70-79	-1.09	(-4.32, 2.13)		6.25	(-1.36, 13.88)	
80+	-3.87	(-8.43, 0.69)		-3.13	(-12.88, 6.61)	
Family member sex – female (vs male)	-0.18	(-1.42, 1.04)	0.77	1.66	(-1.37, 4.71)	0.28
Family member ethnicity – white (vs non-white)	0.81	(-2.67, 4.30)	0.65	6.46	(-4.24, 17.15)	0.24
Next-of-kin/lives with patient (vs lives with patient)			0.10			0.86
Next-of-kin, does not live with patient	-0.93	(-2.93, 1.05)		1.39	(-3.49, 6.28)	
Not next-of-kin, does not live with patient	-1.65	(-3.22, 0.07)		0.48	(-3.49 , 4.46)	
Frequent visitor	5.31	(3.38, 7.23)	<0.001	3.84	(-0.21, 7.91)	0.063
Fixed effects – patient level						
Patient age (per 10 years)	0.26	(-0.20, 0.73)	0.27	2.19	(0.61, 3.78)	0.007
Patient sex – female (vs male)	0.79	(-0.84, 2.43)	0.34	1.29	(-2.67, 5.26)	0.52
Dependency (vs none)			0.44			0.47
Minor or major	1.34	(-0.74, 3.43)		2.91	(-1.48, 7.29)	
Total	0.11	(-7.42, 7.64)		4.27	(-17.36, 25.91)	
Surgical status (vs non-surgical)			0.25			0.68
Planned elective/scheduled	-1.83	(-4.35, 0.68)		-1.09	(-12.59, 10.41)	
Unplanned	-1.35	(-3.41, 0.71)		2.35	(-3.20, 7.91)	
ICNARC Physiology Score (per point)	0.12	(0.01, 0.24)	0.040	0.19	(-0.04, 0.44)	0.12
ICU length of stay (per day)	0.03	(-0.04, 0.11)	0.39	-0.17	(-0.39, 0.03)	0.11
Advanced respiratory support	3.03	(1.08, 4.97)	0.002	--		
Fixed effects – ICU/hospital level						

For peer review only

Hospital type (vs non-university)			0.50			0.55
University	-0.41	(-4.27, 3.46)		-4.44	(-12.41, 3.53)	
University affiliated	1.51	(-1.37, 4.39)		-0.86	(-6.56, 4.83)	
Number of ICU beds (per bed)	0.02	(-0.19, 0.23)	0.85	0.47	(0.02, 0.93)	0.042
Random effects – SD (SE)						
Between ICUs	2.06	(0.66)		3.33	(1.50)	
Within ICUs between patients	17.24	(0.50)		15.84	(1.06)	
Within patients between family members	17.02	(0.40)		16.81	(0.66)	

Coef, coefficient; SE, standard error.

[a] Five patients were missing age group on both the questionnaire and web portal – due to the very small amount of missing data in this key variable, these missing values were not imputed.

For peer review only

Figure S2 Variation across ICUs in the mean: satisfaction with care domain score (A) before and (B) after adjustment; satisfaction with information domain score (C) before and (D) after adjustment; and satisfaction with the decision-making process domain score (E) before and (F) after adjustment

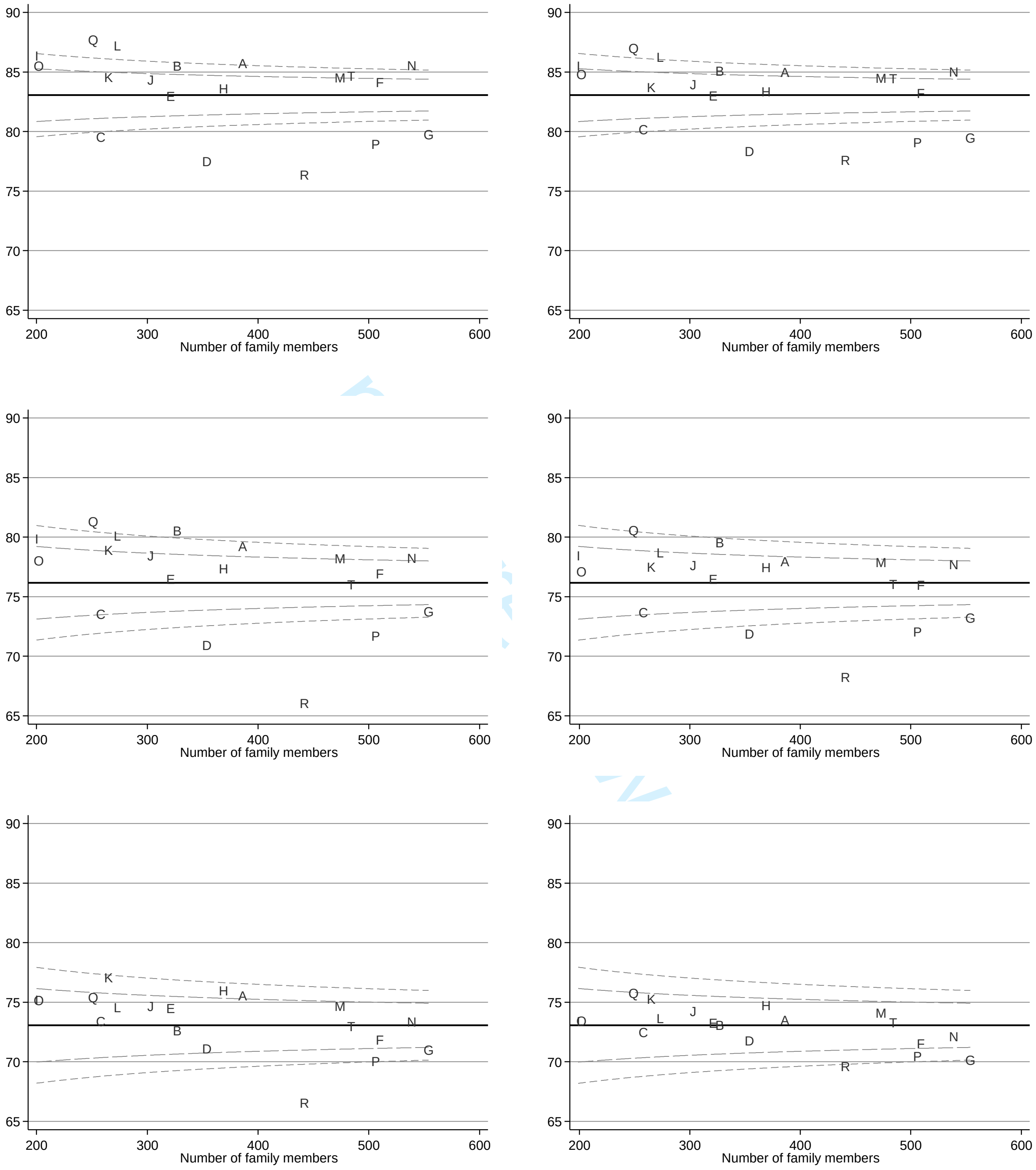

Table S10 Sensitivity analyses – alternative approach to handling missing data (family members of ICU survivors)

Variables	Complete case [N=2,351]			Traditional approach [N=5,756]		
	Coef.	SE	p-value	Coef.	SE	p-value
Constant	72.60	3.18		70.35	2.49	
Family member age, years (vs <30)			0.61			0.20
30-39	0.13	1.40		1.47	0.97	
40-49	0.85	1.22		1.41	0.86	
50-59	0.66	1.20		1.58	0.84	
60-69	0.65	1.30		1.47	0.88	
70-79	0.77	1.47		1.69	0.98	
80+	-3.06	2.26		-1.22	1.50	
Family member sex – female (vs male)	0.94	0.60	0.12	0.21	0.43	0.63
Family member ethnicity – white (vs non-white)	7.58	1.58	<0.001	3.99	1.16	0.001
Next-of-kin/lives with patient (vs lives with patient)			0.071			0.002
Next-of-kin, does not live with patient	-1.69	0.85		-1.36	0.61	
Not next-of-kin, does not live with patient	-1.42	0.72		-1.70	0.50	
Frequent visitor	1.18	0.82	0.15	2.21	0.55	<0.001
Patient age (per 10 years)	-0.09	0.22	0.67	-0.07	0.15	0.64
Patient sex – female (vs male)	-1.20	0.73	0.10	0.13	0.52	0.79
Dependency (vs none)			0.70			0.45
Minor or major	-0.44	0.92		-0.19	0.68	
Total	-2.19	2.98		-3.14	2.51	
Surgical status (vs non-surgical)			0.056			0.47
Planned elective/scheduled	-3.11	1.30		-0.93	0.80	
Unplanned	-0.44	0.88		0.02	0.62	
ICNARC Physiology Score (per point)	0.08	0.05	0.14	0.15	0.04	<0.001
ICU length of stay (per day)	-0.04	0.03	0.28	-0.04	0.03	0.17
Advanced respiratory support	1.39	0.87	0.11	2.40	0.60	<0.001
Hospital type (vs non-university)			0.42			0.34
University	0.56	2.36		1.45	2.22	

For peer review only

University affiliated	2.24	1.72		2.34	1.61	
Number of ICU beds (per bed)	0.07	0.12	0.59	-0.02	0.11	0.83

Coef., coefficient; SE, standard error.

For peer review only

Table S11 Sensitivity analyses – alternative approaches to handling missing data (family members of ICU non-survivors)

Variables	Complete case [N=547]			Traditional approach [N=851]		
	Coef.	SE	p-value	Coef.	SE	p-value
Constant	54.46	7.72		56.28	6.80	
Family member age, years (vs <30)			0.17			0.086
30-39	4.38	3.01		3.14	2.44	
40-49	7.51	2.75		4.87	2.31	
50-59	6.19	2.62		4.50	2.22	
60-69	7.41	2.85		5.94	2.37	
70-79	6.99	3.69		7.07	2.82	
80+	7.52	4.41		0.32	3.61	
Family member sex – female (vs male)	-0.02	1.43	0.99	0.40	1.11	0.72
Family member ethnicity – white (vs non-white)	9.64	4.21	0.022	7.47	3.58	0.037
Next-of-kin/lives with patient (vs lives with patient)			0.97			0.38
Next-of-kin, does not live with patient	0.13	2.20		1.27	1.82	
Not next-of-kin, does not live with patient	-0.32	1.81		-0.82	1.40	
Frequent visitor	1.32	1.96	0.50	0.99	1.51	0.51
Patient age (per 10 years)	0.69	0.66	0.29	1.09	0.55	0.048
Patient sex – female (vs male)	1.56	1.69	0.36	2.02	1.41	0.15
Dependency (vs none)			0.47			0.66
Minor or major	-0.61	1.86		-0.32	1.58	
Total	8.53	7.42		5.59	6.45	
Surgical status (vs non-surgical)			0.84			0.51
Planned elective/scheduled	-0.33	5.61		-4.86	4.22	
Unplanned	-1.38	2.33		-0.44	1.95	
ICNARC Physiology Score (per point)	0.24	0.10	0.022	0.18	0.09	0.041
ICU length of stay (per day)	-0.27	0.09	0.003	-0.33	0.08	<0.001
Hospital type (vs non-university)			0.83			0.77
University	-1.15	3.20		-0.11	3.01	
University affiliated	0.84	2.29		1.49	2.17	

For peer review only

Number of ICU beds (per bed)	0.25	0.19	0.17	0.21	0.17	0.23

Coef., coefficient; SE, standard error.

For peer review only

STROBE Statement—checklist of items that should be included in reports of observational studies

	Item No	Recommendation	Page No
**Title and abstract**	1	(*a*) Indicate the study's design with a commonly used term in the title or the abstract	1
		(*b*) Provide in the abstract an informative and balanced summary of what was done and what was found	2
**Introduction**			
Background/rationale	2	Explain the scientific background and rationale for the investigation being reported	4
Objectives	3	State specific objectives, including any prespecified hypotheses	4
**Methods**			
Study design	4	Present key elements of study design early in the paper	4
Setting	5	Describe the setting, locations, and relevant dates, including periods of recruitment, exposure, follow-up, and data collection	4
Participants	6	(*a*) *Cohort study*—Give the eligibility criteria, and the sources and methods of selection of participants. Describe methods of follow-up<br>*Case-control study*—Give the eligibility criteria, and the sources and methods of case ascertainment and control selection. Give the rationale for the choice of cases and controls<br>*Cross-sectional study*—Give the eligibility criteria, and the sources and methods of selection of participants	4-5
		(*b*) *Cohort study*—For matched studies, give matching criteria and number of exposed and unexposed<br>*Case-control study*—For matched studies, give matching criteria and the number of controls per case	N/A
Variables	7	Clearly define all outcomes, exposures, predictors, potential confounders, and effect modifiers. Give diagnostic criteria, if applicable	4-6
Data sources/ measurement	8*	For each variable of interest, give sources of data and details of methods of assessment (measurement). Describe comparability of assessment methods if there is more than one group	
Bias	9	Describe any efforts to address potential sources of bias	4
Study size	10	Explain how the study size was arrived at	N/A
Quantitative variables	11	Explain how quantitative variables were handled in the analyses. If applicable, describe which groupings were chosen and why	5-6
Statistical methods	12	(*a*) Describe all statistical methods, including those used to control for confounding	5-6
		(*b*) Describe any methods used to examine subgroups and interactions	N/A
		(*c*) Explain how missing data were addressed	5-6
		(*d*) *Cohort study*—If applicable, explain how loss to follow-up was addressed<br>*Case-control study*—If applicable, explain how matching of cases and controls was addressed<br>*Cross-sectional study*—If applicable, describe analytical methods taking account of sampling strategy	
		(*e*) Describe any sensitivity analyses	6

Continued on next page

For peer review only

For peer review only - http://bmjopen.bmj.com/site/about/guidelines.xhtml

Results

Participants	13*	(a) Report numbers of individuals at each stage of study—eg numbers potentially eligible, examined for eligibility, confirmed eligible, included in the study, completing follow-up, and analysed	6
		(b) Give reasons for non-participation at each stage	
		(c) Consider use of a flow diagram	Supplementary materials Figure S1
Descriptive data	14*	(a) Give characteristics of study participants (eg demographic, clinical, social) and information on exposures and potential confounders	Page 6-7 & Tables 1 & 2
		(b) Indicate number of participants with missing data for each variable of interest	Supplementary materials Tables S10 & S11
		(c) *Cohort study*—Summarise follow-up time (eg, average and total amount)	N/A
Outcome data	15*	*Cohort study*—Report numbers of outcome events or summary measures over time	7 & Table 3
		Case-control study—Report numbers in each exposure category, or summary measures of exposure	
		Cross-sectional study—Report numbers of outcome events or summary measures	
Main results	16	(*a*) Give unadjusted estimates and, if applicable, confounder-adjusted estimates and their precision (eg, 95% confidence interval). Make clear which confounders were adjusted for and why they were included	7 & 8, Table 4 & Supplement Tables S7-9 &
		(*b*) Report category boundaries when continuous variables were categorized	
		(*c*) If relevant, consider translating estimates of relative risk into absolute risk for a meaningful time period	
Other analyses	17	Report other analyses done—eg analyses of subgroups and interactions, and sensitivity analyses	10-13 & supplement

Discussion

Key results	18	Summarise key results with reference to study objectives	13-14
Limitations	19	Discuss limitations of the study, taking into account sources of potential bias or imprecision. Discuss both direction and magnitude of any potential bias	13-14
Interpretation	20	Give a cautious overall interpretation of results considering objectives, limitations, multiplicity of analyses, results from similar studies, and other relevant evidence	13-14
Generalisability	21	Discuss the generalisability (external validity) of the study results	13-14

Other information

Funding	22	Give the source of funding and the role of the funders for the present study and, if applicable, for the original study on which the present article is based	15

*Give information separately for cases and controls in case-control studies and, if applicable, for exposed and unexposed groups in cohort and cross-sectional studies.

Note: An Explanation and Elaboration article discusses each checklist item and gives methodological background and published examples of transparent reporting. The STROBE checklist is best used in conjunction with this article (freely

For peer review only - http://bmjopen.bmj.com/site/about/guidelines.xhtml

available on the Web sites of PLoS Medicine at http://www.plosmedicine.org/, Annals of Internal Medicine at http://www.annals.org/, and Epidemiology at http://www.epidem.com/). Information on the STROBE Initiative is available at www.strobe-statement.org.

For peer review only

For peer review only - http://bmjopen.bmj.com/site/about/guidelines.xhtml
